# Zwitterionic Character and Lipid Composition Determine the Behaviour of Glycosylphosphatidylinositol Fragments in Monolayers

**DOI:** 10.1002/cphc.202100002

**Published:** 2021-03-15

**Authors:** Ankita Malik, Peter H. Seeberger, Gerald Brezesinski, Daniel Varón Silva

**Affiliations:** ^1^ Department of Biomolecular Systems Max Planck Institute of Colloids and Interfaces Am Muehlenberg 1 14476 Potsdam Germany; ^2^ Department of Chemistry and Biochemistry Freie Universität Berlin Arnimallee 22 14195 Berlin Germany

**Keywords:** glycosylphosphatidylinositol, monolayers, glycolipids, GPI modifications, lipids

## Abstract

Glycosylphosphatidylinositols (GPIs) are complex glycolipids found in free form or anchoring proteins to the outer leaflet of the cell membrane in eukaryotes. GPIs have been associated with the formation of lipid rafts and protein sorting on membranes. The presence of a conserved glycan core with cell‐specific modifications together with lipid remodelling during biosynthesis suggest that the properties of the glycolipids are being fine‐tuned. We synthesized a series of GPI fragments and evaluated the interactions and arrangement of these glycolipids in monolayers as a 2‐D membrane model. GIXD and IRRAS analyses showed the need of *N*‐acetylglucosamine deacetylation for the formation of hydrogen bonds to obtain highly structured domains in the monolayers and an effect of the unsaturated lipids in formation and localization of the glycolipids within or between membrane microdomains. These results contribute to understand the role of these glycolipids and their modifications in the organization of membranes.

## Graphical abstract

**Role of glycosylphosphatidylinositols**: The glycan and lipid of glycosylphosphatidylinositols (GPIs) contribute to the biophysical properties and interactions of these glycolipids. Using synthetic molecules, we show the role of *N*‐acetylglucosamine deacetylation and lipid remodelling in the formation of domains in monolayers as a 2‐D membrane model. These results contribute to disclose the function of GPIs and their modifications in the activity of GPI glycolipids on the cell membrane.

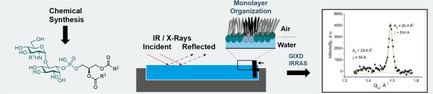

## 1 Introduction

Glycosylphosphatidylinositol (GPI) are glycolipids that are attached as posttranslational modification to the C‐terminus of proteins or are displayed as free glycolipids (free GPIs) on the cell surface of eukaryotes.^[1]^ The complexity of these glycolipids together with the presence of a glycan pseudopentasaccharide core structure and multiple cell‐ and tissue‐specific modifications suggest biological roles of GPIs beyond the anchoring of proteins and glycans to the cell membrane. GPI‐anchored proteins (GPI‐APs) participate in many cellular processes on the membrane such as protein sorting and trafficking,^[2]^ parasitic infections,^[3]^ adhesion and nutrient uptake.^[4]^ GPI‐APs have been associated as components of lipid rafts.^[5]^ However, recent reports describe the presence of GPI‐APs mainly outside of lipid rafts and as obstacles for the diffusion of other membrane proteins.^[6]^

**Figure 1:**
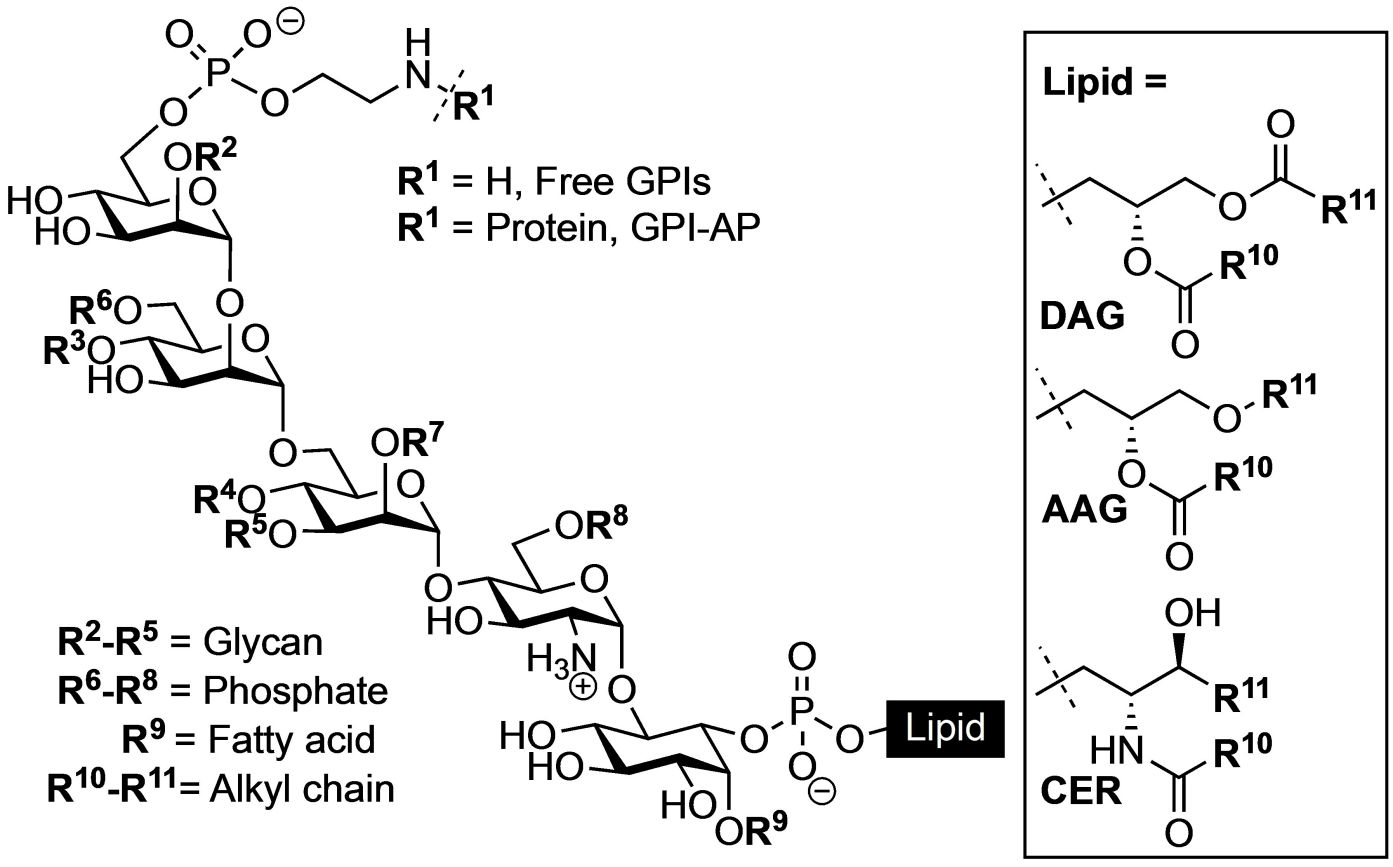
GPI core structure showing the typical modifications. Lipid can be a DAG=diacylglycerol, AAG=alkylacylglycerol, or CER=ceramide.

The lipid part of GPIs can be highly heterogeneous and contain lipid chains of variable length and degree of saturation, influencing the interactions with the lipid bilayer and other molecules in the membrane.^[7]^ During the biosynthesis and trafficking through the Golgi, GPI‐APs are associated to detergent‐resistant domains and exported to the membrane (Figure 1).^[8]^ Studies of GPI‐APs in liposomes and lipid monolayers as cell mimics showed a clustering of GPI‐APs that was primarily attributed to protein‐protein interactions.^[9]^ However, protein aggregation and sorting is also affected by interactions involving the glycan and lipid parts, suggesting interactions between GPI components and other membrane molecules to be responsible for the heterogeneous distribution of GPIs and GPI‐anchored molecules on the cell membrane.^[10]^

The structural requirement and underlying reasons for the participation of GPIs in lipid rafts is unclear.^[11]^ The lipid part of GPIs contains mostly a phospholipid attached to a diacylglycerol (DAG), an alkylacylglycerol (AAG) or a ceramide (CER) with alkyl chains that interact with membranes through van der Waals forces. GPIs possess bulky and flexible head groups that can occupy a larger in‐plane area compared to the lipid part and interact through strong hydrogen bonds between glycans. Interactions in the head group can induce conformational changes leading to temporary membrane domains with highly ordered structure.^[12]^ Therefore, the elucidation of the effect of structural and conformational changes and the interaction of GPIs in membrane models is an important approach to get better insights into the role of GPIs in the organization and phase separation observed in the membrane of eukaryotic cells.

We investigated recently the role of the glycan part and the behaviour of GPIs fragments in model membranes and reported the presence of molecular ordering of these fragments in two‐dimensional monolayer at the air/liquid interface.[^11^, ^12^, ^13^] The analysis showed domains of the lipidated GPI pseudodisaccharide glucosamine‐α‐(1‐6)‐*myo*‐inositol (GlcN‐Ino) with lipid chains having a large tilt angle at the uncompressed state, which changes only slightly upon compression. This ordering was attributed to strong hydrogen bonds between the head groups that resulted in formation of a superlattice with an increased structural rigidity of the zwitterionic fragment. The highly ordered monolayer structure of the GPI fragment **1** was characterized by presence of an alkyl chain lattice and a head group molecular lattice.^[13]^

**Figure 2:**
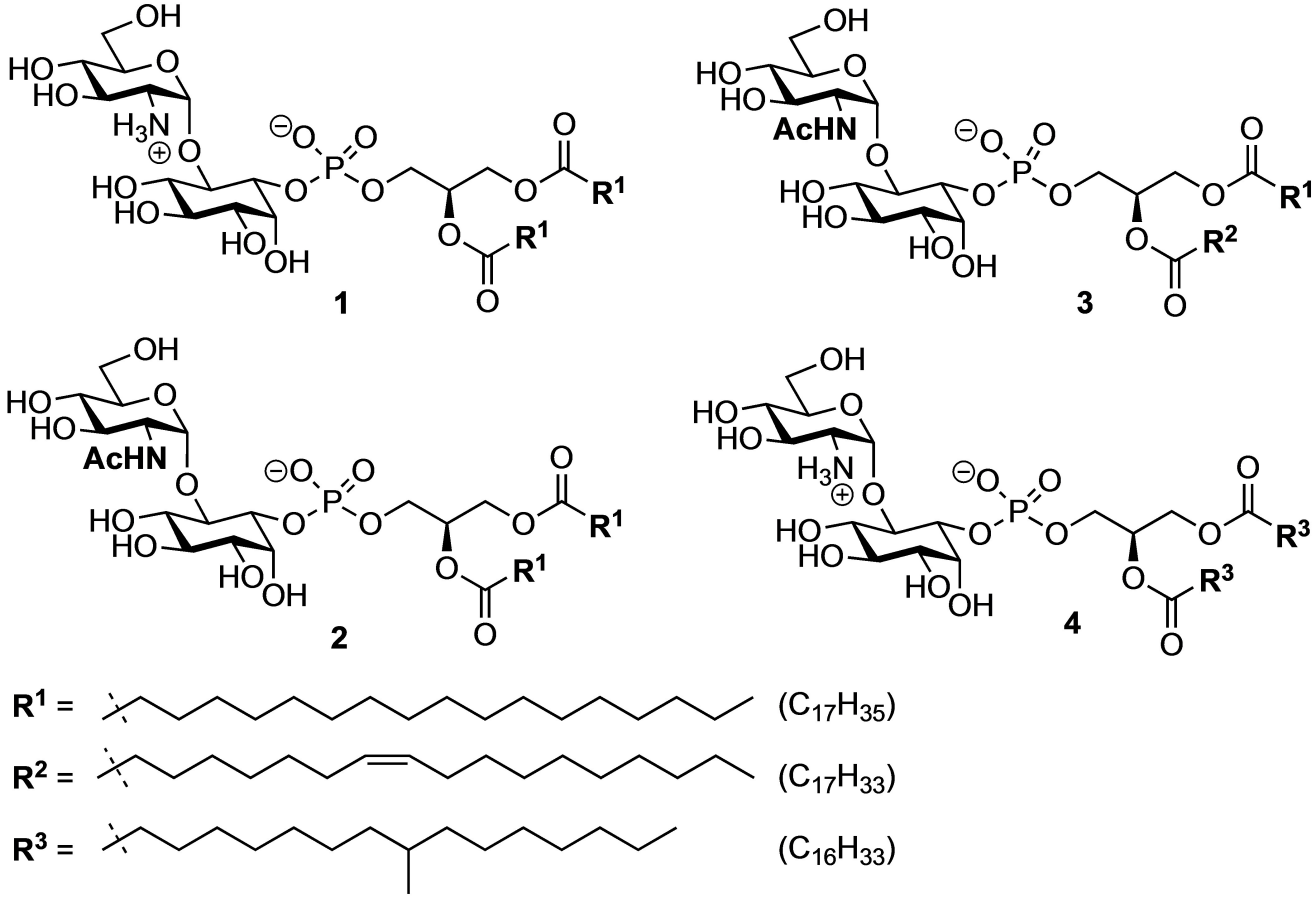
Structure of GPI fragments investigated in this study.

Here, we investigated the role of glucosamine hydrogen bonding between glycan head groups and the role of the lipid chains for the formation of domains in monolayers. We designed and synthesized a set of structures having either glucosamine (GlcN‐Ino) or *N*‐acetylglucosamine (GlcNAc‐Ino) pseudodisaccharide head group and a lipid with saturated, unsaturated or branched alkyl chains (Figure 2). We evaluated the arrangement of these glycolipids in monolayers using grazing incidence X‐ray diffraction (GIXD) and infrared reflection absorption spectroscopy (IRRAS). We show the need of glucosamine for the biophysical behaviour of GPIs and provide further understanding about the importance of lipid remodelling during the biosynthesis and intracellular transport of GPI‐APs.

## 2 Results

### 2.1 Design and Synthesis of the GPI Fragments

Four molecules were designed to investigate the role of glucosamine and lipid composition on the biophysical properties of GPI fragments in monolayers. Glycolipids **2** and **3** containing *N*‐acetylglucosamine (Figure 2) were used to suppress the participation of the amino group in the hydrogen bond formation between the head groups leading to the formation of highly‐structured phases.^[13]^ Fragment **3** containing 1‐stearoyl‐2‐oleyl‐*sn*‐glycerol, a lipid composition commonly found in mammalian GPIs, and glycolipid **4** having branched alkyl chains (Figure 2) were designed to evaluate the role of the alkyl chains modification in the lateral organization of the lipid monolayer.

**Scheme 1:**
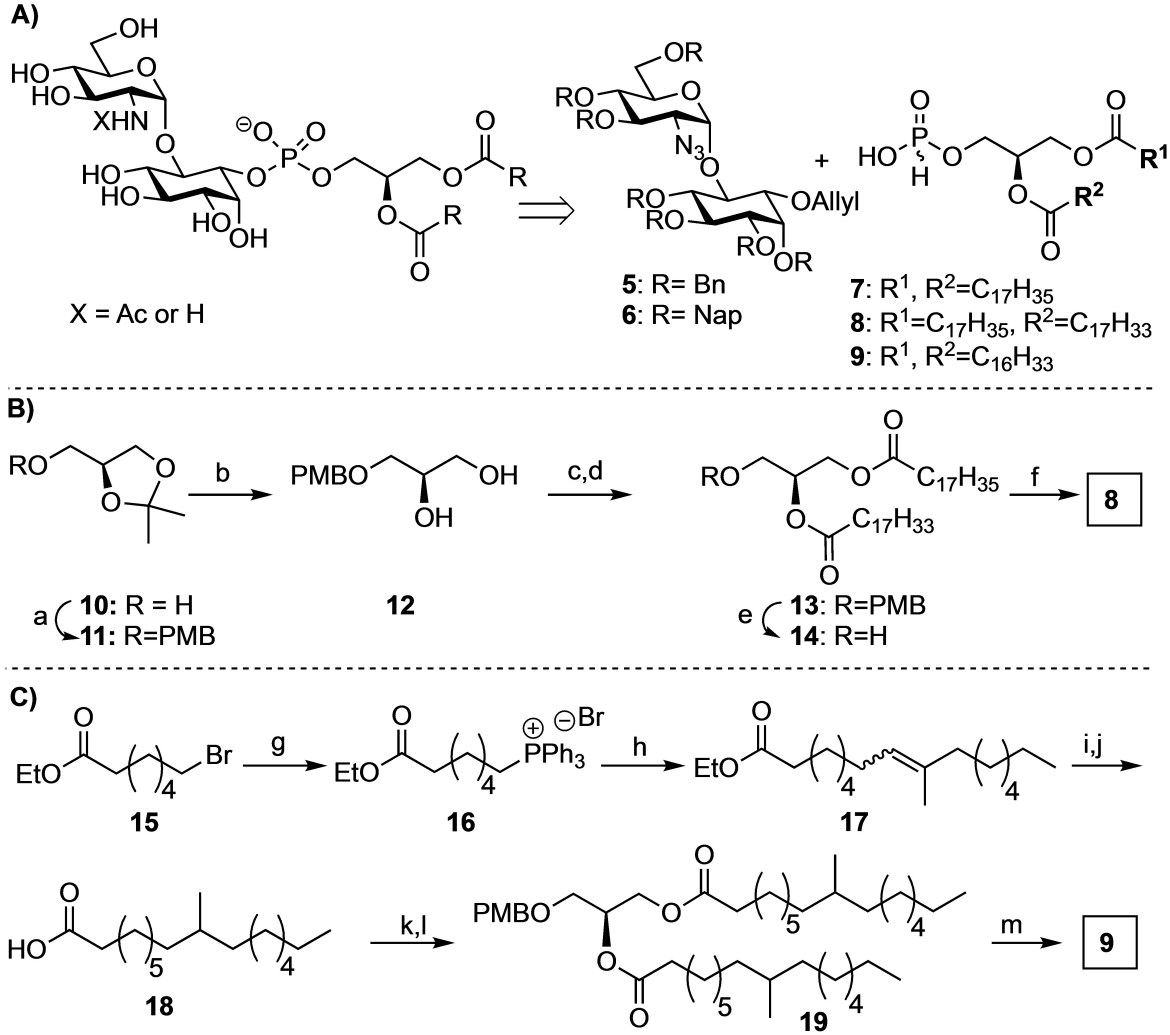
Synthesis of lipidated GPI‐pseudodisaccharides. A) Retrosynthetic analysis for the GPI fragments **1–4**. B) and C) Synthesis of H‐phosphonates **8** and **9**. *Reagents and conditions*: a) PMB−Cl, NaH, DMF, rt, 2 h, 83 %; b) pTSA, H_**2**_O, CH_**2**_Cl_**2**_, rt, 92 %, c) stearic acid, DIC, DMAP CH_**2**_Cl_**2**_, rt, 84 %; d) oleic acid, DIC, DMAP, CH_**2**_Cl_**2**_, rt, 75 %; e) DDQ, H_**2**_O, CH_**2**_Cl_**2**_, rt, 60 %; f) H_**3**_PO_**3**_, PivCl, pyridine, rt, 70 %; g) PPh_**3**_, CH_**3**_CN, 98 %; h) tBuOK, 2‐decanone, −78 °C to −20 °C, THF, 69 % i) Pd/C, H_**2**_, MeOH; 96 %; j) NaOH, THF/MeOH, 83 %, k) **12**, DCC, DMAP, CH_2_Cl_2_ 92 %; l) Pd/C, H_2_, 99 %; m) PivCl, H_3_PO_3_, pyridine, 93 %. PMB=para‐methoxybenzyl, NAP=2‐Naphthylmethyl, pTSA=p‐toluenesulfonic acid, DCC=dicyclohexylcarbodiimide, PivCl=pivaloyl chloride.

The synthesis of GPI fragments **1–4** required two glycans (**5** and **6**) and the H‐phosphonates **7**,^[14]^ **8**, and **9** (Scheme 1). Fragments **1**, **2**, and **4** having saturated alkyl chains were accessible from the benzyl protected pseudodisaccharide **5**.^[15]^ Fragment **3**, containing an unsaturated chain, required the use of the 2‐naphthylmethyl (Nap) protected pseudodisaccharide **6** ^[16]^ and H‐phosphonate **8** (Scheme 1).

The synthesis of the GPI‐fragments started with the preparation of H‐phosphonates **8** and **9**. Protection of commercially available 1,2‐isopropylideneglycerol using PBM chloride under basic conditions and following hydrolysis of the acetal **11** using para‐toluenesulfonic acid (pTsOH) provided the diol **12** for acylation with the fatty acids. First, the primary alcohol was acylated using stearic acid and DIC/DMAP activation. Then, the remaining free alcohol was reacted with oleic acid and the PMB group was removed with DDQ and water to deliver the diacylglycerol **14** in 60 % isolated yield. Phosphitylation of glycerol **14** using phosphonic acid and pivaloyl chloride (PivCl) for activation provided the H‐phosphonate **8** in 70 % yield.

To obtain H‐phosphonate **9**, the branched fatty acid was prepared starting with the synthesis of the phosphonium salt **16** from bromide **15** and triphenylphosphine (PPh_3_).^[17]^ Deprotonation of the phosphonium salt **16** with potassium tert‐butoxide delivered an ylide for the following Wittig olefination of 2‐decanone to obtain the ethyl ester **17** as a mixture of the E‐/Z‐ isomers. A palladium catalyzed hydrogenolysis and saponification of the ethyl ester with potassium hydroxide provided the branched fatty acid **18**. Acylation of the diol **12** with the acid **18** using DIC/DMAP for activation provided the glycerol **19**. Removal of the PMB group by hydrogenolysis and phosphitylation of the released alcohol using phosphonic acid and pivaloyl chloride provided the desired H‐phosphonate **9** in 93 % yield (Scheme 1).

**Scheme 2:**
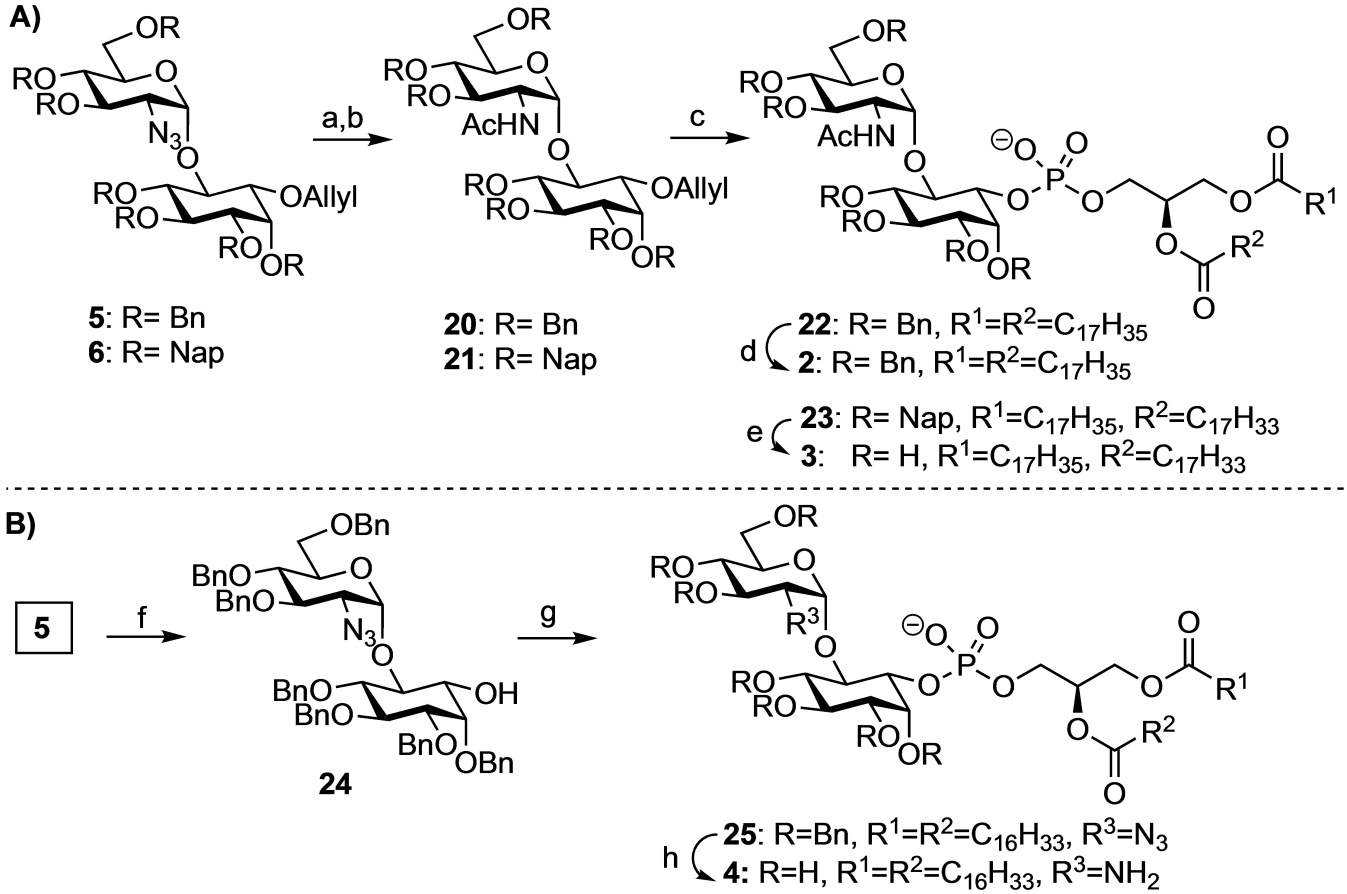
Synthesis of fragments **2**, **3**, and **4**. *Reagents and conditions*: a) Zn, Ac_2_O, AcOH, THF, rt; b) PdCl_2_, MeOH/CH_2_Cl2, rt, 59 % for **20**, 49 % for **21** (over two steps); c) *i*. **7** ^[18]^ or **8**, PivCl, pyridine, rt; *ii*. I_2_, H_2_O, rt, 60 % for **22**, 61 % for **23**; d) Pd(OH)_2_, H_2_, CH_2_Cl_2_:MeOH:H_2_O (3 : 3 : 1), rt, 50 %; e) TFA‐anisole (10 : 1), rt, 80 %; f) PdCl_2_, MeOH/CH_2_Cl_2_, rt, 82 %; g) *i*. **9**, PivCl, pyridine, rt; *ii*. I_2_, H_2_O, rt, 60 %, h) Pd(OH)_2_, H_2_, CH_2_Cl_2_:MeOH:H_2_O (3 : 3 : 1), rt, 52 %. PivCl=pivaloyl chloride, TFA= trifluoroacetic acid.

To assemble the glycolipids **2** and **3** containing *N*‐acetylglucosamine, the azide in the glycans **5** and **6** was reduced to amine using zinc in acetic acid and the resulting amine was acetylated using acetic anhydride and base. Removal of the allyl group from the pseudodisaccharides **20** and **21** using palladium chloride in methanol released the alcohol on inositol to install the phospholipids. Phosphitylation with the corresponding H‐phosphonates **8** and **9** using PivCl activation and following oxidation with iodine/water delivered the lipidated and protected pseudodisaccharides **22** and **23** (Scheme 2). Final deprotection of the hydroxyl groups of the fully protected pseudodisaccharide **22** by hydrogenolysis with palladium delivered the glycolipid **2**. A treatment of the propected pseudodisaccharide **23** with trifluoracetic acid to remove the 2‐naphthylmethyl groups provided glycolipid **3** having an unsaturated lipid chain.

Glycolipid fragment **4** was synthesized using a strategy similar for that to fragment **2**. The allyl group in *pseudo*disaccharide **5** was removed using palladium chloride in methanol to provide alcohol **24**. Following phosphitylation of this alcohol with activated H‐phosphonate **9** and oxidation with iodine/water delivered protected glycolipid **25**. A global deprotection by hydrogenolysis with palladium provided **4** having glucosamine and branched alkyl chains in 52 % yield (Scheme 2 and SI).

### 2.2 Evaluation of Glycolipids in Monolayers

**Figure 3:**
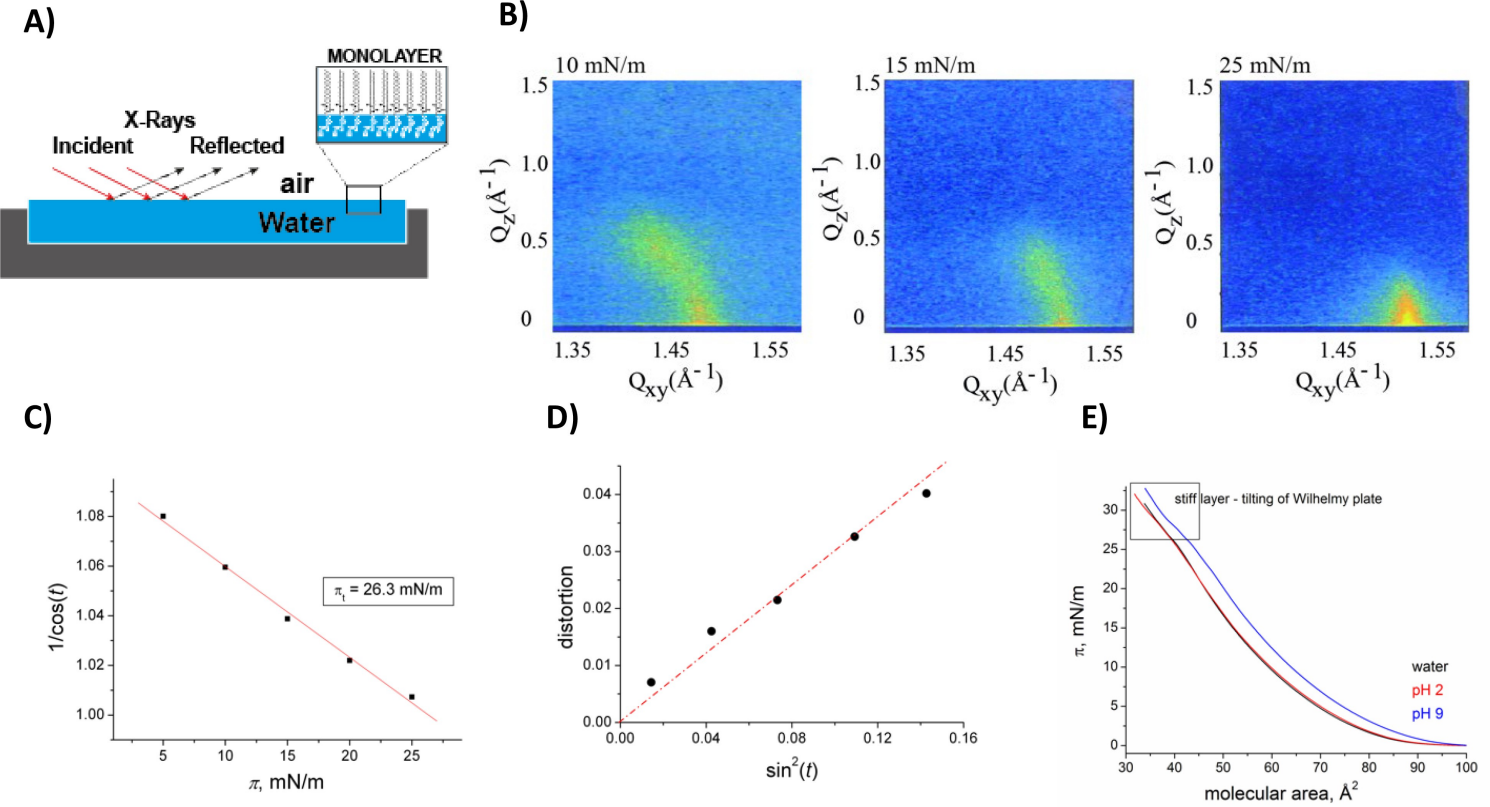
Monolayers of GPI fragment **2** at 20 °C. A) Representation of the analysis system. B) GIXD contour plots (intensity as a function of the out‐of‐plane component Q_z_ and the in‐plane component Q_xy_ of the scattering vector) characterizing the monolayer LC phases on water. C) Variation of the tilt angle t of the alkyl chain versus lateral pressure π. D) Lattice distortion d versus sin^2^(t). E) Molecular area versus lateral pressure isotherms on different subphases.

To investigate the effect of the modifications on the biophysical properties of the glycolipids **1–4**, we evaluated first the monolayer of fragment **2** by GIXD at different lateral pressures (Figure 3A). The GIXD data (Figure 3B, Table S1) revealed that monolayers of **2** were characterized by an orthorhombic lattice of tilted chains transforming to a hexagonal packing of non‐tilted chains at the lateral pressure of ∼26 mN/m (Figure 3C). No head group ordering leading to the highly ordered monolayer structure of the GPI fragment **1** has been observed. The correspondence of zero tilt angle with zero distortion indicates that the lattice distortion in this monolayer is only caused by the tilt of the molecule to optimize their van‐der‐Waals interactions (cross‐sectional chain area of 19.7 Å^2^) and not because of any other molecular interactions as formation of H‐bond networks (Figure 3D).

Surface pressure/molecular area isotherms recorded on subphases at different pH for fragment **2**, containing *N*‐acetylglucosamine to disrupt the zwitterionic character, showed only the phase transition from gaseous to LC phase (re‐sublimation). The isotherm of **2** at pH 9 is shifted to slightly larger molecular areas that was attributed to a stronger electrostatic repulsion by the fully deprotonated phosphate groups. A tilting of the Wilhelmy plate was observed at high surface pressures due to the stiffness of the monolayer (Figure 3E). Noteworthy, the isotherm alone does not explain the observed phase behavior. The molecular areas seen in the isotherm are much larger compared to the ones determined by GIXD. Thus, the only reasonable explanation is a large number of packing defects. This problem is still under investigation but does not influence the general tendency observed for the compounds in this study.

**Figure 4:**
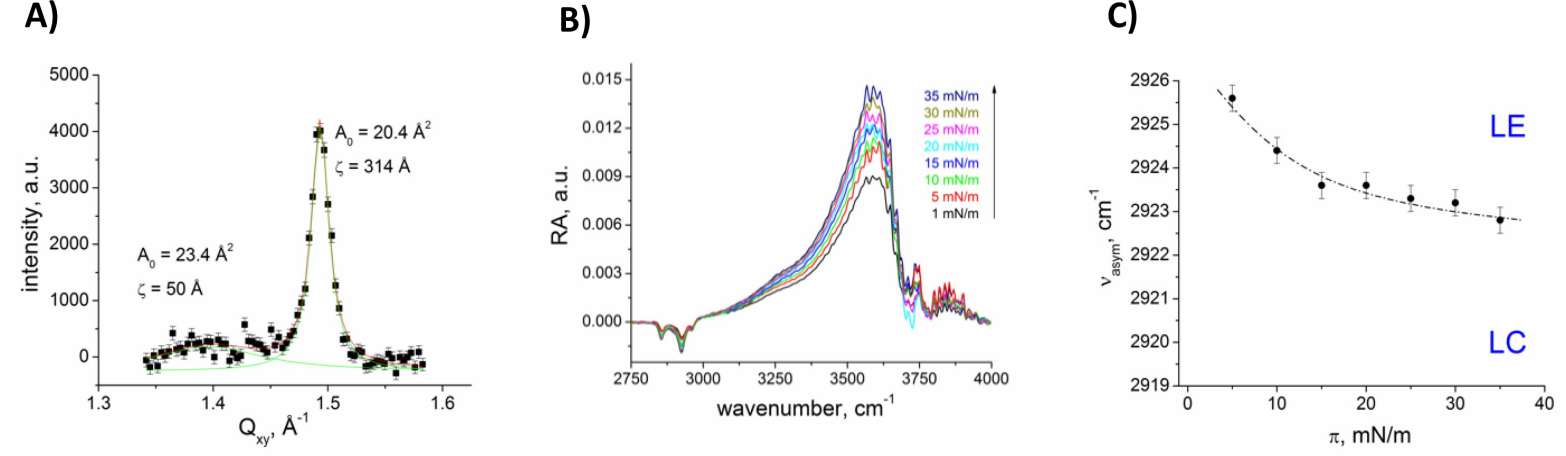
Monolayers of GPI fragment **3** on water at 20 °C. A) Integrated scattering intensity as a function of the in‐plane component Q_xy_ of the scattering vector characterizing the co‐existence of fluid (cross‐sectional area of 23.4 Å^2^) and ordered (cross‐sectional area of 20.4 Å^2^) phases. B) Selected part of IRRA spectra along the isotherm showing the CH2 stretching bands and the OH‐band. C) Wavenumber of the asymmetric CH_2_ stretching vibration versus the lateral pressure π.

The GIXD pattern for the fragment **3**, containing one unsaturated fatty acid chain at the C2‐position and one saturated at the C1‐position of the glycerol backbone as well as an *N*‐acetyl glucosamine, exhibits only one characteristic Bragg peak for a hexagonal ordered structure and a broad peak corresponding to an in‐plane area of about 23 Å^2^ (Figure 4A). In addition, the IRRA spectra demonstrate an increase in effective layer thickness (increasing intensity of the OH‐band) and packing density (shift of the CH_2_‐stretching vibrations to lower wavenumbers) (Figures 4B, 4 C) during compression. These results confirm the idea of a co‐existence of disordered and partially ordered (cross‐sectional chain area of 20.4 Å^2^) phases. The saturated fatty acid is most probably involved in the formation of such an ordered inner phase in the 2‐D membrane model whereas the unsaturated fatty acid is mostly present in the less ordered outer phase.

The isotherms of fragment **4** with two branched fatty acid chains and a zwitterionic head group are very similar at different pH values (Figure S1) in contrast to the results obtained with fragment **2**. The IRRA spectra (Figure S2) prove that the monolayer is in a liquid‐expanded state (high wavenumbers of the CH_2_ stretching vibrations, Figure S4). Therefore, the only first‐order phase transition close to zero pressure seen in the isotherm indicates the transition from gaseous to LE phase. The possible strong head‐group interactions due to hydrogen bonding as observed for GPI fragment **1** cannot overcome the packing problems of alkyl chains with a methyl branch in the middle of the chain.^[19]^

## 3 Discussion

GPI‐anchored proteins are commonly associated with the formation of protein complexes and detergent‐resistant membrane microdomains in eukaryotes.^[10]^ However, recent studies showed that some GPI‐APs do not form domains in the membrane of living cells.^[6]^ Considering the variability of GPI structures, it is not clear if the different behaviour observed depends on the composition of the glycolipid. Thus, it is necessary to investigate the contribution of the GPI components to the formation and organization of membrane domains and interactions in protein complexes. The structure of GPIs contains a conserved core glycan and some specific modifications that may contribute to the interactions between head groups and with the lipids within the membrane.^[20]^ *N*‐acetylglucosamine deacetylation and lipid remodeling are two essential steps in the biosynthesis of GPIs establishing key functionalities for the interactions of GPIs.[^7a^, ^21^] The deacetylation of glucosamine contributes to the zwitterionic character of the glycolipid and polar interactions outside the membrane. The remodeling of lipid chains affects the interactions between alkyl chains and may contribute to the fluidity of the lipid bilayer.^[22]^ However, there is no clear understanding of the mechanisms involving these interactions and how they affect the biological and biophysical properties of GPIs.

Previous studies showed the formation of hydrogen bonds between the head groups of GPI fragments and the formation of highly‐organized phases in monolayers.[^11^, ^12^, ^13^] We assigned the amino group of glucosamine to be the major player in the hydrogen bonding, and consequently, the need for the deacetylation of *N*‐acetylglucosamine to strengthen interactions between the glycans of GPIs. We designed and synthesized four GPI‐fragments (**1**–**4**), two with glucosamine and two with *N*‐acetylglucosamine, and three different alkyl chain compositions. We carried out a comparative analysis of the arrangement of these fragments in monolayers as a 2‐D membrane model and evaluated the role of deacetylation and inter‐lipid chain interactions in the formation of microdomains.

Saturated, unsaturated, and branched lipid chains were incorporated into the fragments to obtain different packing and effects on the fluidity of the monolayers. Thus, the synthesis of the glycolipids required a pseudodisaccharide having either a benzyl (**5)** or a 2‐naphthylmethyl (**6)** ethers as permanent protecting groups, and the corresponding H‐phosphonate (**7, 8 or 9**. H‐Phosphonates **7** and **8** were available by acylation of glycerol **12** using commercial stearic and oleic acid in either a one‐step or two‐step process. In contrast, the preparation of H‐phosphonate **9** needed first the synthesis of the branched fatty acid **18**. The synthesis of this acid involved four steps and a Wittig olefination of 2‐decanone as the key step of the process (Scheme 1c). All diacylglycerols were phosphitylated and used to install the lipids into the glycans using pivaloyl chloride activation.^[23]^ The reduction of the azide and installation of the *N*‐acetyl group were more efficient for all compounds before the removal of the allyl group and lipidation. The removal of the benzyl groups from the intermediates **22** and **25** using palladium‐catalyzed hydrogenolysis delivered the products with linear and branched saturated alkyl chains in good yields. The removal of the naphthyl ethers demanded optimization to avoid the reduction of the double bond and decomposition of the product. The desired glycolipid **3** was accessible by treatment of **23** with anhydrous trifluoroacetic acid and anisole.^[16]^

To study the biophysical properties of glycolipids, we analyzed monolayers of these compounds at the water‐air interface by GIXD and IRRAS.^[24]^ GIXD analysis showed pronounced effects due to the *N*‐acetylglucosamine deacetylation and hence the loss of the zwitterionic character of the glycolipid. The head group of the GPI fragment became more flexible and organized into a non‐distorted hexagonal unit cell. Monolayers of glycolipid **1** formed a highly ordered structure characterized by two lattices, a lattice of alkyl chains and a molecular lattice based on strong hydrogen bonding interactions between the head groups as observed for some glycolipid monolayers.^[25]^ In contrast, monolayers of fragment **2** formed only a lattice of alkyl chains due to the disruption of interactions between the head groups by hydrogen bonding and salt bridges with the amino group. These observations show the importance of hydrogen bonding and electrostatic interactions in the formation of ordered microdomains in the cell membrane. These results confirm the significance of the deacetylation of *N*‐acetylglucosamine in GPIs, and its conserved nature in these glycolipids.^[26]^

The degree of saturation of fatty acid chains in the bilayer membrane structure is known to control membrane fluidity and packing density. The presence of alkyl branches and unsaturation in the lipid chains showed a similar effect in contributing to the fluidity of the membrane model.[^19^, ^27^] Monolayers of fragment **3**, having one saturated and one unsaturated chain, were characterized by the presence of partially ordered and partially disordered domains (Figure 4). Thus, such monolayers showed structures characterized by a lattice of the saturated alkyl chains in the center of an ordered domain (LC) and a more fluid‐like outer part corresponding to a liquid‐expanded domain (LE) with the unsaturated chains projected outwards. So‐called hybrid lipids with one fully saturated and one partially unsaturated chain have been only investigated in lipid mixtures. The hybrid lipid is line active at saturated/unsaturated interfaces and reduces the line tension to stabilize finite‐sized domains.^[28]^ In the present case, we found for the first time that such a hybrid GPI fragment can form ordered microdomains surrounded by fluid‐like boundary layers in a single‐component system. This is a fascinating example for micro‐phase separation in a single‐component system known to occur in liquid‐crystalline polymers.^[29]^ The observed microphases are the result of the competition of the incompatibilities of unsaturated and saturated chains chemically linked to the same backbone. Therefore, such GPI fragment might have outstanding significance in biological multi‐component systems.

The monolayer of GPI fragment **4**, containing two methyl‐branched chains, is fluid and without any ordered structures at all lateral pressures along the isotherm. IRRAS analysis confirmed these results showing high wavenumbers for the −CH_2_‐stretching vibrations. In comparison, the monolayer of fragment **3** shows co‐existing domains (partial liquid‐expanded and liquid‐condensed phases). These findings emphasise the role of the GPI lipid remodeling as a fine‐tuning parameter for the localization of the GPIs on membranes and the introduction of specific glycans and lipid modifications.^[7a]^

The lipid composition in GPIs is highly variable and includes lipids with alkyl‐acyl glycerol or ceramides and fatty acid chains of different lengths. The significance of these lipid variants and the interaction within them and with other molecules on membranes is also unknown. Some GPIs also possess an additional lipid at the inositol and phosphoethanolamine units. Thus, further studies with GPIs having these modifications are the next step to understand the role of lipid chains in glycolipids and the significance in the biological interactions within the membrane.

## 4 Conclusions

We used chemical synthesis to obtain a set of glycosylphosphatidylinositol fragments and evaluated the biophysical properties of these molecules in molecularly thin films at the air‐liquid interface. We showed the need of deacetylation of *N*‐acetylglucosamine for the interaction between GPI head groups by hydrogen bonding and its contribution to the formation of higly‐ordered rigid domains in the membrane model. This study also shows the role of the lipid composition in the localization of the glycolipids in membrane domains and the effect of lipids with saturated and unsaturated alkyl chains to be in the boundary of rigid domains that is an important requirement for interactions with other molecules at the cell membrane. These results demonstrated the need for such modifications as a fine‐tuning parameter affecting the behavior of the GPI‐APs on membranes. Our results strongly support the idea of functional lipid domains in cell membranes as an important general mechanism for the generation of nanoclusters of GPI‐anchored proteins.^[7b]^

## Experimental Section

### Synthesis of the GPI fragments

The pseudodisaccharide **1** was synthesized following previous reports. The experiments for the synthesis of the glycolipids **2**–**4** are described in the supporting information.

### Solution of the Glycolipids

For the monolayer experiments, 1 mM solutions of all the fragments were prepared in a mixture of chloroform (Merck, Germany; purity >99.8 %), methanol (Merck, Germany; purity >99.9 %) and ultrapure water (Millipore, resistivity of 18 MΩ cm) in a 6 : 2 : 0.2 volume ratios.

### Surface pressure‐area Isotherms

The pressure/area (π/A) isotherms were recorded during compression of the monolayer on a computer‐interfaced Langmuir trough (R&K, Potsdam, Germany) including a surface pressure microbalance with filter paper Wilhelmy plate. The results were plotted as surface pressure (π) versus the area per molecule. The bare water surface was checked for purity by compression before each measurement. The temperature of the Milli‐Q Millipore water subphase was maintained at 20 °C by an external thermostat. The Langmuir layers were prepared by spreading the chloroform: methanol: water solutions of the fragments at the air/water interface. Before compression, the monolayers were left to equilibrate for 15 minutes in order to allow the evaporation of the spreading solvents. Each measurement was repeated at least two times to prove the reproducibility of results. To avoid dust contamination of the interface and to ensure a constant humidity, the Langmuir trough was placed in a sealed box.

### Grazing incidence X‐ray diffraction (GIXD)

The grazing incidence X‐ray diffraction measurements were carried out at the undulator beamline P08 using the Langmuir trough GID setup at PETRA III, DESY (Hamburg, Germany). The setup is equipped with a temperature‐controlled Langmuir trough (R&K, Potsdam, Germany), which is enclosed in a sealed, helium‐filled container. The synchrotron X‐ray beam is monochromated to an energy of 15 keV (wavelength of 0.827 Å) and is adjusted to strike the helium/water interface at a grazing incidence angle α_i_=0.07° illuminating approximately 1×50 mm^2^ of the monolayer surface. A MYTHEN detector (DECTRIS Ltd., Switzerland) measures the diffracted signal and is rotated to scan the in‐plane Q_xy_ component values of the scattering vector. A Soller collimator in front of the MYTHEN restricted the in‐plane divergence of the diffracted beam to 0.09°. The vertical strips of the MYTHEN measure the out‐of‐plane Q_z_ component of the scattering vector between 0.0 and 1.4 Å^−1^. The diffraction data consist of Bragg peaks at diagnostic Q_xy_ values obtained by summing the diffracted intensity over a defined vertical angle or Q_z_‐window. The in‐plane lattice repeat distances d of the ordered structures in the monolayer are calculated from the Bragg peak positions: d=2π/Q_xy_. To estimate the extent of the crystalline order in the monolayer, the in‐plane coherence length L_xy_ is approximated from the full‐width at half‐maximum (fwhm) of the Bragg peaks by L_xy_∼0.9(2π)/fwhm (Q_xy_) using the measured fwhm(Q_xy_) corrected for the instrumental resolution. Integrating the diffracted intensity normal to the interface over the Q_xy_ window of the diffraction peak yields the corresponding Bragg rod. The thickness of the scattering unit is estimated from the fwhm of the Bragg rod using 0.9(2π)/fwhm (Q_z_).

### Infrared reflection absorption spectroscopy (IRRAS)

Infrared reflection absorption spectra were recorded using the Vertex 70 FT‐IR spectrometer (Bruker, Germany), equipped with a liquid‐nitrogen cooled MCT detector and coupled to a Langmuir film balance, which was placed in a sealed container (an external air/water reflection unit (XA‐511, Bruker)) to guarantee a constant vapor atmosphere. Using a KRS‐5 (thallium bromide and iodide mixed crystal) wire grid polarizer, the IR‐beam was polarized parallel (p) or vertical (s) and focused on the fluid subphase at an angle of incidence of 40°. A computer controlled ‘trough shuttle system’ enables us to choose between the compartment with the sample (subphase with spread layer) and a reference compartment (pure subphase). The single‐beam reflectance spectrum from the reference trough was taken as background for the single‐beam reflectance spectrum of the monolayer in the sample trough to calculate the reflection absorption spectrum as ‐log(R/R_0_) in order to eliminate the water vapor signal. FTIR spectra were collected at a resolution of 8 cm^−1^ using 200 scans for s‐polarized light and 400 scans for p‐polarized light.

## Conflict of interest

The authors declare no conflict of interest.

## Supporting information

As a service to our authors and readers, this journal provides supporting information supplied by the authors. Such materials are peer reviewed and may be re‐organized for online delivery, but are not copy‐edited or typeset. Technical support issues arising from supporting information (other than missing files) should be addressed to the authors.

Supplementary
