# Supplementary material for: Zwitterionic Character and Lipid Composition Determine the Behaviour of Glycosylphosphatidylinositol Fragments in Monolayers
Source: Chemphyschem. 2021 Mar 15;22(8):757–63. doi: 10.1002/cphc.202100002 (PMC8251720; doi:10.1002/cphc.202100002)
Supplement: Supplementary file 1 — Supplementary [file CPHC-22-757-s001.pdf]

ChemPhysChem

Supporting Information

Zwitterionic Character and Lipid Composition Determine the Behaviour of Glycosylphosphatidylinositol Fragments in Monolayers

Ankita Malik, Peter H. Seeberger, Gerald Brezesinski, and Daniel Varón Silva*

Supporting Information

General Methods for Synthetic Chemistry	2
Synthesis of H-phosphonate 8	2
Synthesis of H-Phosphonate 9	4
Synthesis of Glycolipid 1	7
Synthesis of Glycolipid 2	9
Synthesis of Glycolipid 3	10
Synthesis of glycolipid 4	13
Biophysical Studies	14
NMR Spectra	17
References	38

General Methods for Synthetic Chemistry

All chemicals were of reagent grade and all anhydrous solvents were of high-purity grade and used as supplied except noted otherwise. Reactions were performed in oven-dried glassware under an inert argon atmosphere unless noted otherwise. Reagent grade thiophene was dried over activated molecular sieves prior to use. Pyridine was distilled over CaH_2 prior to use. Sodium hydride suspension was washed with hexane and THF and stored in an anhydrous environment. Benzyl bromide was passed through activated basic aluminum oxide prior to use. Analytical thin layer chromatography (TLC) was performed on Merck silica gel 60 F_{254} plates (0.25 mm). Compounds were visualized by UV irradiation or heating the plate after dipping in staining solution. The staining solutions were cerium sulfate-ammonium molybdate (CAM) solution, basic potassium permanganate solution, acidic ninhydrin-acetone solution, or 3-methoxyphenol-sulfuric acid solution (sugar stain). Flash column chromatography was carried out using a forced flow of the indicated solvent on Sigma Aldrich silica gel high purity grade 60 Å (230-400 mesh particle size, for preparative column chromatography).

^{1}H, ^{13}C and ^{31}P-NMR as well as all 2D-spectra (COSY, TOCSY, HSQC and HMBC) were recorded on a Varian 400 (400 MHz), a Varian 600 (600 MHz), a Bruker 400 (400 MHz) or a Bruker Ascend 400 (400 MHz) spectrometer dissolving the sample in $CDCl_3$ (7.26 ppm ^{1}H, 77.1 ppm ^{13}C), D_2O (4.79 ppm ^{1}H), MeOD (4.87 ppm and 3.31 ppm ^{1}H, 49.00 ppm ^{13}C), Acetone-d_6 (2.05 ppm and 2.84 ppm ^{1}H, 206.26 ppm and 29.84 ppm ^{13}C) unless otherwise stated. The coupling constants (J) are reported in Hertz (Hz). Splitting patterns for ^{1}H NMR data are indicated as s, singlet; d, doublet; t, triplet; q, quartet; br, broad singlet; dd, doublet of doublets; m, multiplet; dt, doublet of triplets and h, hextet. Signals were assigned by means of COSY, TOCSY, HSQC, HMBC spectra and version thereof. ESI-MS analyses were performed on a Waters Xevo G2-XS Q-TOF spectrometer with an Acquity H-class UPLC. MALDI-MS were recorded on a Bruker Autoflex-speed MALDI-TOF spectrometer. Infrared (FTIR) spectra were recorded as thin films on a Perkin Elmer Spectrum 100 FTIR spectrophotometer equipped with an ATR unit. Optical rotations were measured with a Schmidt & Haensch UniPol L 1000 polarimeter at a concentration (c) expressed in g/100 mL. HPLC supported purifications were conducted using Agilent 1100 and Agilent 1200 systems.

Synthesis of H-phosphonate 8

(R)-3-((4-methoxybenzyl)oxy)propane-1,2-diol **(12)**

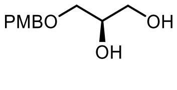

To a stirred solution of commercially available (S)-2,3-O-isopropylideneglycerol **10** (0.50 g, 3.81 mmol) in anhydrous DMF (10 mL) was added NaH (0.18 g, 7.60 mmol) at 0 °C. After 20 min, PMBCl (0.77 mL, 5.72 mmol) was added. The reaction mixture was warmed to room temperature and was stirred for 12 h. The reaction was quenched with MeOH, and diluted with Et_2O. The organic layer was extracted with water. The combined organic layers were further washed with brine, dried over Na_2SO_4 and concentrated to obtain **4-14**. The crude product was dissolved in CH_2Cl_2 (12 mL), para-toluenesulfonic acid (pTSA.5H_2O, 0.06 g, 0.3 mmol) was added and the reaction mixture was stirred at room temperature for 12 h. The reaction was

quenched with triethylamine and concentrated. Flash chromatography was performed to obtain the diol **12** (0.60 g, 2.83 mmol, 75%) as colorless oil. R_f = 0.30 (EtOAc/hexane 2:1); ^{1}H NMR (400 MHz, $CDCl_3$) δ 7.27 (d, *J* = 7.4 Hz, 2H, Ar-H), 6.91 (d, *J* = 8.3 Hz, 2H, Ar-H), 4.51 (s, 2H), 3.90 (td, *J* = 5.8, 2.9 Hz, 1H), 3.83 (s, 3H, CH_3-O), 3.73 (dd, *J* = 11.4, 3.9 Hz, 1H, CH-O), 3.65 (dd, *J* = 11.4, 5.3 Hz, 1H), 3.56 (qd, *J* = 9.6, 5.0 Hz, 2H). ^{13}C NMR (101 MHz, $CDCl_3$) δ 159.41(Ar-O), 129.71 (Ar), 129.51(2C Ar), 113.91(2C Ar), 73.29 (Ar-O-CH_2-), 71.57 (CH-O), 70.52 (O-CH_2-), 64.15, 55.32 (O-CH_3).

(S)-1-((4-methoxybenzyl)oxy)-3-(stearoyloxy)propan-2-yl octadec-9-enoate **(13)**

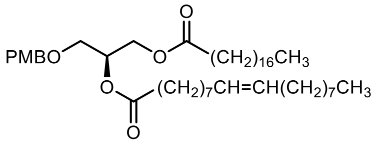

To a stirred solution of diol **12** (0.50 g, 2.36 mmol) in CH_2Cl_2 (15 mL) were added N,N-dimethylaminopyridine (DMAP) (0.28 g, 2.36 mmol), stearic acid (0.67 g, 2.36 mmol) and diisopropylcarbodiimide (DIC) (0.37 mL, 2.36 mmol). The mixture was stirred at room temperature for 5 h. The reaction was extracted with CH_2Cl_2, washed with brine, dried over Na_2SO_4 and concentrated. The residue was purified by flash column chromatography to obtain monoacylated glycerol and was subjected to second coupling. The intermediate alcohol (0.67 g, 1.43 mmol) was dissolved in CH_2Cl_2 (10 mL), DMAP (0.17 g, 1.41 mmol), oleic acid (0.48 mL, 1.54 mmol) and DIC (0.24 mL, 1.54 mmol) were added and reaction mixture was stirred at room temperature for 12 h. The reaction was extracted with CH_2Cl_2, washed with brine, dried over Na_2SO_4 and concentrated. The crude product was purified by flash column chromatography to obtain **13** (0.78 g, 1.05 mmol, 75%) as white solid. R_f = 0.40 (EtOAc/hexane 1:4); ^{1}H NMR (400 MHz, $CDCl_3$) δ 7.26 (d, *J* = 8.2 Hz, 2H), 6.90 (d, *J* = 8.4 Hz, 2H), 5.37 (qq, *J* = 8.7, 5.3, 4.5 Hz, 2H), 5.25 (dd, *J* = 6.2, 4.0 Hz, 1H), 4.55 – 4.43 (m, 2H), 4.35 (dd, *J* = 11.9, 3.8 Hz, 1H), 4.19 (dd, *J* = 11.9, 6.4 Hz, 1H), 3.83 (s, 3H), 3.58 (d, *J* = 5.5 Hz, 2H), 2.32 (dt, *J* = 17.1, 7.5 Hz, 4H), 2.03 (q, *J* = 6.6 Hz, 4H), 1.62 (h, *J* = 7.3 Hz, 4H), 1.39 – 1.25 (m, 49H), 0.90 (t, *J* = 6.6 Hz, 6H). ^{13}C NMR (101 MHz, $CDCl_3$) δ 173.47, 173.14, 159.29, 130.02, 129.73, 129.33(2C), 113.80(2C), 72.96, 70.01, 67.87, 62.70, 55.27, 34.34, 34.14, 31.96, 31.94, 29.80, 29.74, 29.70, 29.67, 29.56, 29.53, 29.40, 29.36, 29.33, 29.24, 29.16, 29.13, 29.09, 27.25, 27.20, 24.97, 24.91, 22.73, 14.17. ESI-MS (*m/z*): $[M+Na]^+$ cald 765.600, obsd 765.459.

(S)-1-hydroxy-3-(stearoyloxy)propan-2-yl octadec-9-enoate **(14)**

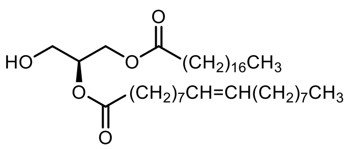

To a stirred solution of bilipid **13** (0.60 g, 0.81 mmol) in 10:1 mixture of CH_2Cl_2 and water was added DDQ (0.18 g, 1.61 mmol) at room temperature. After 12 h, sat. $NaHCO_3$ solution was added. The mixture was extracted with CH_2Cl_2, dried over Na_2SO_4 and concentrated. The crude product was purified by flash column chromatography to obtain **14** (0.30 g, 0.48 mmol, 60%).

R_f = 0.35 (EtOAc/hexane 2:3); ^{1}H NMR (400 MHz, $CDCl_3$) 5.42 – 5.31 (m, 2H), 5.11 (p, *J* = 5.0 Hz, 1H), 4.43 – 4.22 (m, 2H), 3.75 (t, *J* = 5.8 Hz, 2H), 2.36 (q, *J* = 8.0 Hz, 4H), 2.12 – 1.94 (m, 4H), 1.73 – 1.61 (m, 2H), 1.30 (d, *J* = 20.0 Hz, 51H), 0.94 – 0.87 (m, 6H). ESI-MS (*m/z*): $[M+Na]^+$ cald 645.543, obsd 645.14.

(2R)-1-((hydroxyhydrophosphoryl)oxy)-3-(stearoyloxy)propan-2-yl octadec-9-enoate **(8)**

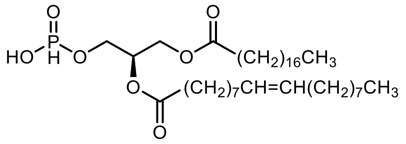

Diacylglycerol **14** (0.20 g, 0.32 mmol) and phosphonic acid (0.13 g, 1.61 mmol) were dissolved and co-evaporated with anhydrous pyridine three times and dried under high vacuum for 2 h. The mixture was dissolved in anhydrous pyridine (5 mL) and a solution of pivaloyl chloride (0.20 mL, 1.61 mmol) in pyridine (2 mL) was added. The solution was stirred for 16 h at room temperature. After the completion of reaction, the reaction mixture was concentrated and purified by Et_3N deactivated silica gel column chromatography to obtain *H*-phosphonate **8** (0.15 g, 0.22 mmol, 70%) as a white solid. R_f = 0.7 (MeOH/ CH_2Cl_2 = 1:9) ; ^{1}H NMR (400 MHz, $CDCl_3$) δ 12.38 (s, 1H), 5.47 – 5.31 (m, 2H), 5.24 (s, 1H), 4.48 – 3.96 (m, 3H), 3.10 (s, 5H), 2.32 (q, *J* = 7.6 Hz, 4H), 2.03 (q, *J* = 6.4 Hz, 3H), 1.33 – 1.21 (m, 44H), 0.90 (t, *J* = 6.7 Hz, 6H). ^{31}P NMR (162 MHz, $CDCl_3$) δ 4.66. ESI-MS (*m/z*): $[M-H]^-$ cald 685.52, obsd 685.0.

Synthesis of H-Phosphonate 9

Ethyl-(7-Triphenylphosphonium bromide)-heptanoate **16**

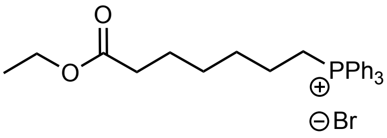

Triphenylphosphine 5.53 g (21.09 mmol) was dissolved in 10 ml of ethyl acetate and 4.7 mL (5 g, 21.09 mmol) of ethyl 7-bromoheptanoate **15** were added. The reaction mixture was heated using microwaves for 2 hours at 160°C (pressure 5-6 bar) under stirring. The resulting dark reaction mixture was dissolved in dichloromethane and concentrated in the rotary evaporator. The remaining resin was separated on silica gel chromatography using dichloromethane/methanol 9:1 to 5:1 to give 10.4 g (20.8 mmol, 98%) of phosphonium salt **16**[1]. R_f= 0.35 (n-Hex/AcOEt 3:1), ^{1}H NMR (600 MHz, $CDCl_3$) δ 7.90 – 7.84 (m, 6H, 6 x Ph-H), 7.80 – 7.76 (m, 3H, 3 x Ph-H), 7.69 (td, *J* = 7.8, 3.4 Hz, 6H, 6 x Ph-H), 4.08 (q, *J* = 7.1 Hz, 2H, P-C$\underline{H_2}$-), 3.93 – 3.87 (m, 2H, CO-CH_2-C$\underline{H_2}$-), 2.24 (t, *J* = 7.4 Hz, 2H, O-C$\underline{H_2}$-), 1.69 (q, *J* = 7.6 Hz, 2H, -CH_2-), 1.62 (td, *J* = 9.1, 8.6, 4.3 Hz, 2H), 1.58 – 1.51 (m, 3H), 1.33 (tt, *J* = 10.0, 6.4 Hz, 2H), 1.22 (t, *J* = 7.1 Hz, 3H, -CH_3). ESI- HRMS for $C_{27}H_{32}BrO_2P$: M_{calc} 498.1323, M_{found}: 499.151 $(M+H)^+$.

Ethyl 8-methylhexadec-7-enoate **17**

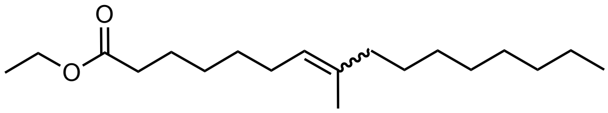

Phosphonium salt **16** (9 g, 18.02 mmol) was dissolved in 150 mL of anhydrous THF, cooled to -78 °C and a solution of t-BuOK (2.022 g, 18.02 mmol) in 50 mL of THF was added dropwise. The reaction mixture was stirred at -78 °C for 10 minutes and then allowed to warm to -30°C over 1 hour (color changes from colorless to yellow to orange and then red-orange). The mixture was cooled to -78 °C and 2-decanone (2.82, 18.02 mmol) was added dropwise. Then the mixture was warmed up to -20 °C over 2 hours and then removed from the cooling bath and stirred at room temperature for 3 hours. After complete conversion of the ketone by TLC (color changed from light orange to yellow), the reaction was quenched by addition of solid ammonium chloride and 300 mL of DCM. The reaction was dried with Na_2SO4, filtered, and evaporated under reduced pressure. The remaining was separated on a silica gel column (elute with Hexane, 2% and then 5% EtOAc in Hexane) to afford 3.73 g (12.61 mmol) of the Z-/E-isomers mixture of **17** that was directly used in the next step without further separation. R*f* = 0.44 (n-Hex/AcOEt 4:1), ^{1}H NMR (400 MHz, $CDCl_3$) δ 5.09 (t, *J* = 7.0, 1.3 Hz, 1H, -CH=), 4.12 (q, J = 7.1 Hz, 2H, -CH_2-CH=), 2.33 – 2.25 (dd, *J*= 7.6 Hz, 2H, O-CH_2-), 2.02 – 1.91 (m, 4H, -CH_2-), 1.67 – 1.56 (m, 5H), 1.40 – 1.20 (m, 18H), 0.91 – 0.85 (m, 3H, CH_3 (Et)). ESI HRMS for $C_{19}H_{36}O_2$ M_{calcd}: 296.2715, M_{found}: 319.290 $(M+Na)^+$.

8-Methylhexadecanoic acid **18**

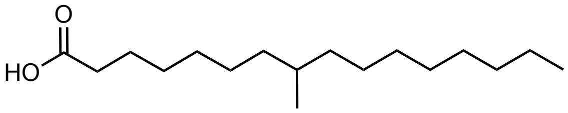

The isomer-mixture of ester **17** (2.65 g, 8.9 mmol) was dissolved in 60 mL of a 3:1 chloroform/methanol mixture. Palladium (10% on carbon, 0.95 g, 0.9 mmol) was added and hydrogen was bubbled through the solution under stirring. After 10 minutes the bubbling was stopped and the reaction mixture was stirred under a hydrogen atmosphere for 36 hours. The mixture was filtered through a pad of celite, volatiles were removed under reduced pressure and the crude product was immediately used in the next step. (2.15 g, 8.01 mmol, 96 %).

The reduced ester was dissolved in 15 mL of a THF/MeOH mixture (2:1). NaOH (495 mg, 12.38 mmol) was added as a solution in 4 mL of water. The resulting mixture was heated at 60°C for 3 hours. The mixture was neutralized with 1M HCl and extracted four times with DCM. The organic extracts were dried over Na_2SO4 and concentrated. Purification on a silica gel column deliver 2.04 g (7.56 mmol, 83%) of the 8-methyl-hexadecanoic acid **18**. R*f* = 0.62 (CH_2Cl_2/MeOH, 10:1) ^{1}H NMR (400 MHz, $CDCl_3$) δ: 2.35 (t, *J* = 7.5 Hz, 2H, C=O-CH_2-), 1.63 (p, *J* = 7.4 Hz, 2H,(C=O-CH_2-CH_2-), 1.40 – 1.16 (m, 21H), 1.14 – 1.00 (m, 2H), 0.91 – 0.85 (t, *J* = 6.7 Hz, 3H, CH_3), 0.83 (d, *J* = 6.5 Hz, 3H, CH_3-branch); ^{13}C NMR (101 MHz, cdcl3) δ 180.6 (C=O, acid), 37.1 (C=O-CH_2-), 37.0 (C=O-CH_2-CH_2-), 34.1, 32.7, 32.7, 31.9, 31.9, 30.0, 29.7, 29.7, 29.6, 29.4, 29.1, 27.1, 26.8, 24.7, 22.7, 22.7, 19.7 (CH_3), 14.1 (CH_3- branch), ATR-IR2ν (cm^{-1}), 2954.5, 2922.7, 2853.7, 2672.2, 2211.6, 2141.2, 2067.2, 2036.2, 2019.7, 1709.6 (C=O), 1463.9, 1412.5, 1377.3; ESI HRMS for $C_{17}H_{34}O_2$ M_{calc} 270.2559, M_{found} 269.2481 $(M-H)^-$.

(S)-3-((4-methoxybenzyl)oxy)propane-1,2-diyl bis(8-methylhexadecanoate) **13**

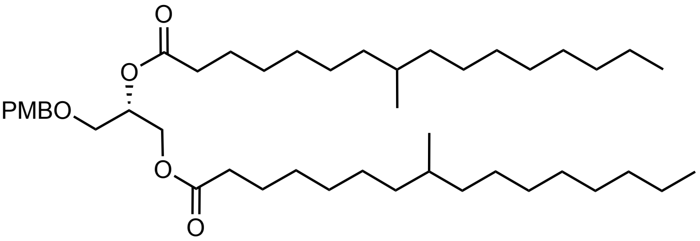

Dicyclohexylcarbodiimide (DCC, 0.763 g, 3.70 mmol) was added at 5°C to a stirred solution of (R)-3-((4-methoxybenzyl)oxy)propane-1,2-diol (0.341g, 1.608 mmol), N,N-dimethylpyridin-4-amine (0.059 g 0.482 mmol) and 8-methylhexadecanoic acid (1.0g 3.7 mmol) in DCM (8.0ml). The reaction was stirred for overnight at room temperature. The precipitate was filtered and the solvent was removed under vacuum. The product was purified by silica gel column chromatography using 5% EtOAc in n-hexane to obtain the diacylglycerol **13** (1.06 g, 1.478 mmol) in 92% yield. ^{1}H NMR (400 MHz, $CDCl_3$) δ : 7.25 ‐ 7.20 (m, 2H, Ar-H), 6.89 ‐ 6.84 (m, 2H, Ar-H), 5.22 (ddt, *J* = 6.6, 5.2, 2.6 Hz, 1H, -CH- glycerol)), 4.52 ‐ 4.41 (m, 2H, -CH_2- glycerol), 4.33 (dd, *J* = 11.9, 3.7 Hz, 1H, -CH_{2a}- glycerol), 4.17 (dd, *J* = 11.9, 6.5 Hz, 1H, -CH_{2b}- glycerol), 3.79 (s, 3H, Ar-O-CH_3), 3.55 (dd, *J* = 5.1, 1.2 Hz, 2H, Ar-CH_2-), 2.29 (dt, *J* = 16.7, 7.5 Hz, 4H, 2x CO-CH_2-), 1.59 (dq, *J* = 10.3, 7.4 Hz, 5H), 1.41 – 1.16 (m, 46H), 1.14 – 1.00 (m, 4H), 0.91 – 0.80 (m, 12H). ^{13}C NMR (101 MHz, $CDCl_3$) δ 173.4 (C=O ester), 173.3 (C=O ester), 173.1, 173.1, 159.3, 129.7 (Ar), 129.3 (Ar), 113.8 (Ar), 72.9 (-CH_2-Ar), 70.0 Ar-CH_2-O glycerol), 67.8 (-CH- glycerol), 62.7 (CH_2-O glycerol), 55.2 (CO-CH_2-), 55.2 (CO-CH_2-), 37.1 (-CH2-), 37.0 (-CH2-), 34.3 (-CH_2-), 34.1 (-CH2-), 32.7, 31.9, 31.9, 30.0, 29.7, 29.6, 29.6, 29.4, 29.2, 29.1, 27.1, 26.9, 25.0, 24.9, 22.7, 19.7 (CH_3), 14.1 (CH_3-branch). ESI HRMS calculated for $C_{45}H_{80}O_6$, M_{calc} 716,5955, M_{found} 739.5893 $(M+Na)^+$.

Triethylammonium (2R)-2,3-bis((8-methylhexadecanoyl)oxy)propyl phosphonate **9**

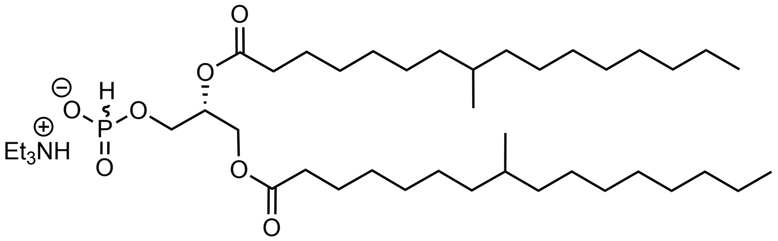

Step 1: Palladium on carbon (0.208 g, 0.195 mmol) was added to a solution of the protected diacylglycerol **13** (0.8g 1.16mmol) in EtOAc (5 ml) and $CHCl_3$ (5 ml). Hydrogen gas was passed through a solution for 10 mins and the reaction mixture was stirred for 45 min under hydrogen atmosphere. The mixture was filter over celite, concentrated under vacuum and the remaining containing the free alcohol (0.66 g 1,106 mmol, 99%) was used in the further reaction without purification.

Step 2: The diacylglycerol (0.667 g 1.116 mmol) and phosphonic acid (0.274 g 3.35mmol) were dissolved in pyridine anhydrous (10 ml) under argon atmosphere. Pivaloyl chloride (0.165 ml, 0.161 g 1.339 mmol) was added dropwise to the stirred solution. The reaction mixture was stirred for 16h. The solvent was evaporated and the reaction remaining was dissolved in CH2Cl2 and purified on a silica gel column deactivated with triethylamine to yield 0.790 g

(1.04 mmol) of the H-phosphonate **9** in 93% yield. *Rf* = 0.27 (CH_2Cl_2/MeOH, 10:1); : ^{1}H NMR (400 MHz, cdcl3) δ 11.95 (s, 1H), 7.49 (s, 0.5 H, P-H), 5.88 (s, 0.5H, P-H), 5.09 (t, J = 5.4 Hz, 1H), 4.25 (dd, J = 12.0, 3.5 Hz, 1H), 4.04 (dd, J = 12.0, 6.5 Hz, 1H), 3.91 (d, *J* = 6.7 Hz, 2H), 2.97 (p, *J* = 6.8 Hz, 5H), 2.17 (q, *J* = 7.7 Hz, 4H), 1.47 (q, J = 7.6, 7.2 Hz, 5H), 1.29 – 1.02 (m, 53H), 1.02 – 0.91 (m, 4H), 0.80 – 0.68 (m, 12H); ^{13}C NMR (101 MHz, $CDCl_3$) δ 173.2 (C=O Ester), 172.8 (C=O Ester), 70.1 (CH_2-O glycerol), 70.1 (CH- glycerol), 62.3 (CH_2-CO), 62.1 (CH_2-CO), 45.5, 37.0, 36.9, 36.9, 34.1, 33.9, 32.6, 31.8, 29.9, 29.6, 29.6, 29.3, 29.1, 29.1, 27.0, 26.8, 24.8, 24.8, 22.6, 19.6, 14.0 (CH_3-), 8.4 (CH_3 branch). ^{31}P NMR: (162 MHz, $CDCl_3$) δ: 4.07; ATR IR (cm^{-1}) ν = 2954.4 (C-H), 2923.5 (C-H) 2853.7 (C-H), 1739.1 (C=O), 1464.4, 1418.1, 1377.1, 1215.4, 1162.9, 1090.8, 1058.7, 1022.4, 987.0; ESI HRMS calculated for $C_{37}H_{73}O_7P$, M_{calc} 659,5021, M_{found} 658.5012 $(M-H)^-$.

Synthesis of Glycolipid 1

3,4,6–Tri-O-benzyl-2-azido-2-deoxy-α-D-glucopyranosyl-(1→6)-2,3,4,5-tetra-O-benzyl-D-myo-inositol **(24)**

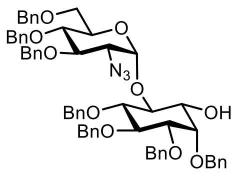

To a stirred solution of **5** (0.28 g, 0.27 mmol) in MeOH/CH_2Cl_2 (3:1, 7 mL) was added $PdCl_2$ (0.01 g, 0.054 mmol) at room temperature. The reaction was stirred for 12 h and quenched with Et_3N and concentrated. The crude residue was purified by flash column chromatography to obtain **24** (0.22 g, 0.22 mmol, 82%). R_f = 0.4 (EtOAc/hexane = 1:1) ^{1}H NMR (400 MHz, Chloroform-*d*) δ 7.48 – 7.02 (m, 51H), 5.42 (d, *J* = 3.6 Hz, 1H), 5.07 – 4.31 (m, 20H), 4.16 – 3.84 (m, 8H), 3.76 – 3.31 (m, 9H), 3.25 – 3.14 (m, 2H), 3.05 – 2.97 (m, 1H).^{13}C NMR (101 MHz, $CDCl_3$) δ 138.55, 138.45, 138.41, 138.19, 138.07, 137.77, 128.44, 128.43, 128.40, 128.33, 128.26, 128.24, 128.21, 128.20, 128.04, 128.00, 127.94, 127.90, 127.83, 127.75, 127.70, 127.65, 127.61, 127.59, 127.43, 127.31, 98.37, 81.97, 81.10, 80.89, 80.72, 80.43, 78.05, 77.32, 77.20, 77.00, 76.68, 75.82, 75.44, 75.23, 74.74, 73.52, 73.33, 72.88, 72.39, 70.79, 67.37, 64.07. ESI-MS (*m*/*z*): $[M+Na]^+$ cald 1020.441 obsd 1020.2.

3,4,6–Tri-O-benzyl-2-azido-2-deoxy-α-D-glucopyranosyl-(1→6)-1-O-(1,2-O-distearoyl-sn-glycerol)-phosphate-2,3,4,5-tetra-O-benzyl-myo-inositol **(26)**

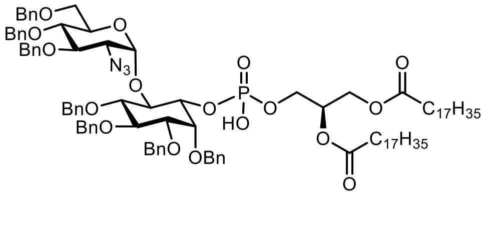

Disaccharide **24** (0.05 g, 0.05 mmol) and *H*-phosphonate **7** (0.05 g, .08 mmol) were co-evaporated with pyridine for three times and dried under high vacuum for 2 h. The mixture was dissolved in anhydrous pyridine (5 mL) and a solution of pivolyl chloride (0.01 mL, 0.08 mmol) in pyridine (1 mL) was added. The solution was stirred for 48 h at room temperature. After 48 h, iodine (0.06 g, 0.25 mmol) and water (0.2 mL) were added and reaction was stirred for 4 h. The reaction mixture was quenched with $Na_2S_2O_3$ and extracted with CH_2Cl_2. The organic layer were concentrated and purified by Et_3N deactivated silica gel flash column chromatography to obtain bilipidated disaccharide **26** (0.05 g, 0.03 mmol, 60%). R_f = 0.7 ($MeOH/CH_2Cl_2$ = 1:9) ^1H NMR (400 MHz, $CDCl_3$) δ 7.45 (d, *J* = 7.1 Hz, 2H), 7.40 – 7.29 (m, 23H), 7.29 – 7.18 (m, 20H), 7.18 – 6.97 (m, 6H), 5.87 (d, *J* = 3.7 Hz, 1H), 5.26 (dt, *J* = 8.8, 4.4 Hz, 1H), 5.00 (dd, *J* = 32.9, 11.3 Hz, 4H), 4.88 (s, 2H), 4.83 – 4.70 (m, 6H), 4.60 (dd, *J* = 35.6, 11.8 Hz, 2H), 4.49 – 4.24 (m, 6H), 4.19 – 3.99 (m, 8H), 3.78 – 3.71 (m, 1H), 3.59 (dd, *J* = 9.8, 2.1 Hz, 1H), 3.55 – 3.46 (m, 3H), 3.43 – 3.34 (m, 3H), 3.23 (dd, *J* = 10.3, 3.7 Hz, 1H), 3.03 (dt, *J* = 11.1, 5.5 Hz, 10H), 2.27 (dp, *J* = 10.2, 5.7, 5.1 Hz, 6H), 1.58 (s, 8H), 1.27 (d, *J* = 4.7 Hz, 73H), 0.90 (t, *J* = 6.8 Hz, 6H). ^{13}C NMR (101 MHz, $CDCl_3$) δ 173.46, 173.09, 139.80, 138.77, 138.48, 138.45, 138.13, 138.08, 138.00, 128.41, 128.29, 128.27, 128.25, 128.20, 128.17, 128.09, 128.07, 127.96, 127.86, 127.76, 127.67, 127.57, 127.52, 127.44, 127.40, 127.29, 127.15, 127.00, 96.77, 81.88, 80.98, 79.77, 78.35, 76.09, 75.65, 75.16, 74.78, 74.71, 73.29, 72.26, 69.95, 67.96, 63.80, 63.17, 62.75, 52.91, 45.57, 34.30, 34.09, 31.96, 29.76, 29.71, 29.59, 29.58, 29.41, 29.38, 29.19, 24.93, 24.89, 22.73, 14.18, 8.53, 8.00. ^{31}P NMR (162 MHz, $CDCl_3$) δ -1.83. ESI-MS (*m*/*z*): $[M+NH_3]^+$ cald 1702.981 obsd 1702.997.

2-amino-2-deoxy-α-D-glucopyranosyl-(1→6)-1-O-(1,2-O-distearoyl-sn-glycerol)-phosphate-D-myo-inositol **(1)**[2]

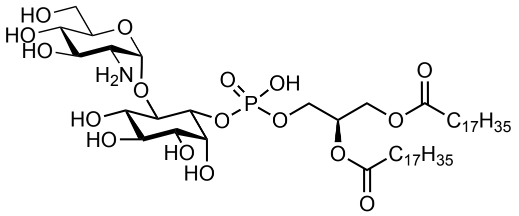

To the stirred solution of *pseudo*-disaccharide **26** (20.0 mg, 0.012 mmol) in mixture of $CH_2Cl_2/MeOH/H_2O$ (3:3:1, 5 mL) at room temperature was added $Pd(OH)_2$ on C (17 mg, 0.024 mmol, 20% Pd content). Hydrogen gas was bubbled through the solution for 15 min and the reaction mixture was stirred under an atmosphere of hydrogen gas for additional 24 h. The $Pd(OH)_2$ was removed by filtration through a pad of celite and the solution was concentrated. The crude product was purified by size exclusion chromatography on a LH20 column using $CH_2Cl_2/MeOH/H_2O$ (3:3:1) as the solvent mixture to obtain the bilipidated disaccharide **1** (6.0 mg, 0.006 mmol, 50%), ^1H NMR (400 MHz, MeOD) δ 5.13 (s, 1H), 4.82 (d, *J* = 7.4 Hz, 1H), 4.06 (dd, *J* = 9.5 Hz, 1H), 3.92 – 3.78 (m, 1H), 3.57 (dt, *J* = 24.1, 6.3 Hz, 1H), 3.51 – 3.28 (m, 6H), 3.17 (q, J = 7.4 Hz, 4H), 2.95 (m, 1H), 2.32 (q, *J* = 7.0, 6.4 Hz, 4Hf), 1.71 – 1.51 (m, 4H), 1.30 (d, J = 26.7 Hz, 60H), 0.89 (d, J = 6.2 Hz, 6H); ^{31}P NMR (162 MHz, MeOD) δ 0.15. ESI-MS (*m*/*z*) for $C_{51}H_{98}NO_{17}P$: M_{calc} 1027.657, M_{obsd} 1026.751 $[M-H]^-$.

Synthesis of Glycolipid 2

3,4,6–Tri-O-benzyl-2-acetamide-2-deoxy-α-D-glucopyranosyl-(1→6)-1-O-allyl-2,3,4,5-tetra-O-benzyl-D-myo-inositol **(20)**

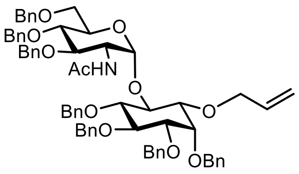

Activated zinc (0.11 g, 1.73 mmol) was suspended in THF (10 mL) and transferred to the pseudodisaccharide **5** (0.12 g, 0.12 mmol). Ac_2O (0.033 mL, 0.35 mmol) and AcOH (0.007 mL, 0.115 mmol) were added and reaction mixture was stirred at room temperature for 4 h. The reaction mixture was filtered through celite and washed with saturated $NaHCO_3$ twice. The organic phase was dried over Na_2SO_4 and concentrated. The crude product was purified by flash column chromatography to obtain **20** (0.087 mg, 0.082 mmol, 71%). R $_f$ = 0.3 (EtOAc/hexane = 1:1) ^{1}H NMR (400 MHz, $CDCl_3$) δ 7.33 (dd, J = 14.5, 7.4 Hz, 4H), 7.27 – 7.00 (m, 60H), 5.66 (ddt, J = 16.4, 10.3, 6.1 Hz, 1H), 5.18 (d, J = 3.3 Hz, 1H), 5.16 – 5.15 (m, 1H), 5.13 – 5.05 (m, 3H), 4.94 – 4.75 (m, 7H), 4.76 – 4.63 (m, 10H), 4.63 – 4.60 (m, 3H), 4.60 – 4.53 (m, 4H), 4.42 (dd, J = 24.7, 11.4 Hz, 3H), 4.35 – 4.18 (m, 2H), 4.19 – 3.98 (m, 3H), 3.99 – 3.90 (m, 11H), 3.82 (dd, J = 12.0, 6.0 Hz, 2H), 3.75 – 3.64 (m, 4H), 3.64 – 3.48 (m, 4H), 3.47 – 3.18 (m, 5H), 3.11 (dd, J = 10.0, 2.0 Hz, 2H), 1.76 (s, 3H). ^{13}C NMR (101 MHz, $CDCl_3$) δ 169.91, 138.75, 138.61, 138.43, 138.29, 138.22, 138.13, 133.52, 128.49, 128.38, 128.35, 128.28, 128.24, 128.09, 128.03, 127.90, 127.82, 127.78, 127.68, 127.60, 127.54, 127.40, 119.11, 100.30, 83.12, 81.99, 81.20, 80.84, 79.73, 79.19, 78.23, 75.82, 75.54, 74.99, 74.79, 74.25, 73.41, 73.04, 72.83, 72.09, 71.13, 68.75, 53.49, 23.29. ESI-MS (*m*/*z*): $[M+H]^+$ cald 1054.5 obsd 1054.3, $[M+Na]^+$ cald 1076.5 obsd 1076.4

3,4,6–Tri-O-benzyl-2-acetamide-2-deoxy-α-D-glucopyranosyl-(1→6)- 1-O-(1,2-O-distearoyl-sn-glycerol)-phosphate -2,3,4,5-tetra-O-benzyl-D-myo-inositol **(22)**

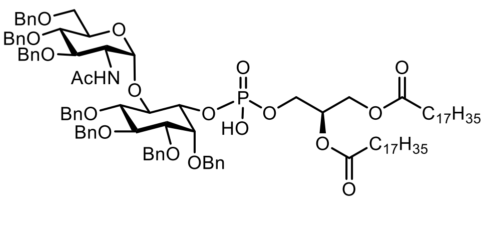

To a stirred solution of **20** (0.07 g, 0.062mmol) in MeOH/ CH_2Cl_2 (3:1) was added $PdCl_2$ (4 mg, 0.019 mmol) at room temperature. The reaction was stirred for 12 h, quenched with TEA and concentrated. The crude product was purified by flash column chromatography to obtain **20a** (0.04 g, 0.04 mmol, 72%) R_f = 0.35 (EtOAc/hexane = 3:2). Disaccharide **20a** (0.04 g, 0.04 mmol) and H-phosphonate **7**[3] (0.09 g, 0.13 mmol) were co-evaporated with pyridine for three times and dried under high vacuum for 2 h. The mixture was dissolved in anhydrous pyridine (7 mL) and a solution of pivolyl chloride (0.016 mL, 0.13 mmol) in pyridine (1 mL) was added.

The solution was stirred for 48 h at room tmeperature. After 48 h, iodine (0.11 g, 0.44 mmol) and water (0.3 mL) were added and the reaction was stirred for 4 h. The reaction mixture was quenched with $Na_2S_2O_3$ and extracted with CH_2Cl_2. The organic layers were concentrated and the crude product was purified with Et_3N deactivated silica gel flash column chromatography to obtain bilipidated disaccharide **22** (0.045 g, 0.027 mmol, 60%). R_f = 0.6 (MeOH/ CH_2Cl_2 = 1:9) ^{1}H NMR (400 MHz, $CDCl_3$) δ 7.49 – 7.09 (m, 40H), 5.50 (d, *J* = 2.4 Hz, 2H), 5.32 (d, *J* = 3.4 Hz, 1H), 5.21 (dd, *J* = 6.6, 3.0 Hz, 1H), 5.05 – 4.89 (m, 2H), 4.88 – 4.61 (m, 7H), 4.47 (dd, *J* = 23.7, 11.5 Hz, 1H), 4.42 – 4.24 (m, 4H), 4.22 – 3.88 (m, 8H), 3.83 – 3.66 (m, 1H), 3.47 – 3.34 (m, 3H), 2.33 – 2.15 (m, 4H), 2.07 (s, 3H), 1.53 (s, 2H), 1.25 (d, *J* = 9.5 Hz, 51H), 0.89 (t, *J* = 6.8 Hz, 6H). ^{31}P NMR (162 MHz, $CDCl_3$) δ -1.12. ESI-MS (*m*/*z*): $[M+H]^+$ cald 1702.0 obsd 1702.6 $[M-H]^-$ cald 1699.99 obsd 1699.8.

2-acetamide-2-deoxy-α-D-glucopyranosyl-(1→6)-1-O-(1,2-O-distearoyl-sn-glycerol)-phosphate-D-myo-inositol **(2)**

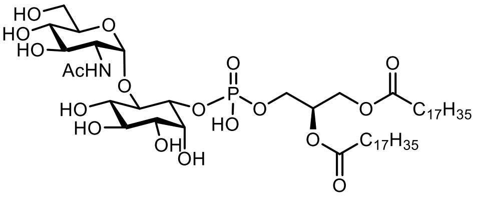

To a stirred solution of pseudodisaccharide **22** (30.0 mg, 0.018 mmol) in mixture of CH_2Cl_2/MeOH/H_2O (3:3:1, 5 mL) at room temperature was added $Pd(OH)_2$ on C (17 mg, 0.024 mmol, 20% Pd content). Hydrogen gas was bubbled through the solution for 15 min and the reaction mixture was stirred under an atmosphere of hydrogen gas for additional 24 h. The $Pd(OH)_2$ was removed by filtration through a pad of celite and the solution was concentrated. The crude product was purified by size exclusion chromatography on a LH20 column using CH_2Cl_2/MeOH/H_2O (3:3:1) as the solvent mixture to obtain the bilipidated disaccharide **2** (9.0 mg, 0.009 mmol, 50%). ^{1}H NMR (400 MHz, Methanol-d_4) δ 7.27 (s, 1H), 5.04 (d, *J* = 5.6 Hz, 2H), 4.01 (ddd, *J* = 23.1, 11.1, 5.5 Hz, 2H), 3.94 – 3.61 (m, 5H), 3.65 – 3.41 (m, 8H), 3.41 – 3.25 (m, 3H), 3.09 (q, *J* = 7.3 Hz, 5H), 2.96 – 2.73 (m, 0H), 2.32 – 2.22 (m, 3H), 2.21 (dd, *J* = 13.4, 7.5 Hz, 1H), 2.00 – 1.91 (m, 1H), 1.53 (s, 2H), 1.17 (s, 44H), 1.03 (d, *J* = 6.5 Hz, 4H), 0.83 – 0.71 (m, 6H). ^{31}P NMR (162 MHz, MeOD) δ 0.44. MALDI-MS (*m*/*z*): $[M+Na]^+$ cald 1092.657 obsd 1092.904 $[M-H]^-$ cald 1069.664 obsd 1069.374

Synthesis of Glycolipid 3

3,4,6–Tri-O-(2-naphthyl)methyl -2-acetamide-2-deoxy-α-D-glucopyranosyl-(1→6)- 1-O-allyl-2,3,4,5-tetra-O-(2-naphthyl)methyl -D-myo-inositol **(21)**

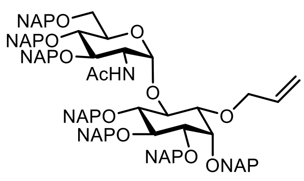

Activated zinc (0.1 g, 0.1 mmol) was suspended in THF and transferred to a/b mixture of pseudodisaccharide **6**[4]. Ac_2O (0.03 mL, 0.33 mmol) and AcOH (0.006 mL, 0.011mmol) were added and reaction mixture was stirred at room temperature for 4 h. The reaction mixture is filtered through celite and washed with saturated $NaHCO_3$ twice. Organic phases were dried over Na_2SO_4 and concentrated. The crude residue was purified with flash column chromatography to obtain **21** (0.12 g, 0.086 mmol, 79%). R_f = 0.2 (EtOAc/hexane = 1:1) ^{1}H NMR (400 MHz, $CDCl_3$) δ 7.90 – 7.31 (m, 109H), 7.21 (ttd, *J* = 16.6, 8.3, 1.4 Hz, 4H), 5.74 – 5.60 (m, 1H), 5.44 (d, *J* = 3.4 Hz, 1H), 5.36 – 5.23 (m, 1H), 5.23 – 5.10 (m, 5H), 5.10 – 4.95 (m, 11H), 4.91 (dd, *J* = 23.6, 12.3 Hz, 5H), 4.88 – 4.57 (m, 5H), 4.50 (td, *J* = 10.1, 3.4 Hz, 1H), 4.45 – 4.20 (m, 5H), 4.23 – 4.01 (m, 4H), 3.98 – 3.78 (m, 5H), 3.77 – 3.55 (m, 7H), 3.54 – 3.44 (m, 3H), 3.26 (dd, *J* = 10.0, 2.0 Hz, 1H), 1.88 (s, 2H). ^{13}C NMR (101 MHz, $CDCl_3$) δ 169.95 (C=O), 136.27, 136.18, 136.07, 135.87, 135.68, 135.58, 135.46, 133.32, 133.23, 133.18, 133.12, 133.00, 132.95, 132.91, 132.89, 132.83, 128.30, 128.13, 128.09, 128.04, 127.98, 127.94, 127.92, 127.86, 127.75, 127.68, 127.62, 126.65, 126.57, 126.44, 126.40, 126.27, 126.24, 126.19, 126.13, 126.10, 126.08, 126.03, 125.98, 125.90, 125.84, 125.81, 125.76, 125.66, 118.90, 116.02, 100.37 (C-1), 83.16, 82.03, 81.10, 80.63, 79.95, 78.91, 78.45, 77.28, 75.90, 75.65, 75.05, 74.79, 74.30, 73.55, 73.17, 72.72, 72.12, 71.18, 68.81, 60.47, 53.47, 29.76, 23.47, 21.14, 14.26.

3,4,6–Tri-O-(2-naphthyl)methyl-2-acetamide-2-deoxy-α-D-glucopyranosyl-(1→6)-2,3,4,5-tetra-O-(2-naphthyl)methyl -D-myo-inositol **(21a)**

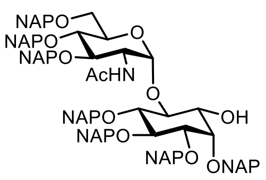

To a stirred solution of **21** (0.12 g, 0.086 mmol) in MeOH/ CH_2Cl_2 (3:1) was added $PdCl_2$ (6 mg, 0.034 mmol) at room temperature. The reaction was stirred for 12 h and quenched with Et_3N and concentrated. The crude residue was purified with flash column chromatography to obtain **21a** (0.07 g, 0.05 mmol, 59%). R_f = 0.25 (EtOAc/hexane = 3:2) ^{1}H NMR (400 MHz, $CDCl_3$) δ 7.80 – 7.68 (m, 17H), 7.70 – 7.63 (m, 13H), 7.61 (d, *J* = 8.6 Hz, 13H), 7.61 – 7.36 (m, 43H), 7.40 – 7.32 (m, 13H), 7.34 – 7.21 (m, 12H), 7.19 (s, 9H), 6.94 (dd, *J* = 8.4, 1.3 Hz, 2H), 6.04 (d, *J* = 9.1 Hz, 1H), 5.27 (d, *J* = 3.4 Hz, 1H), 5.16 (d, *J* = 11.8 Hz, 1H), 5.07 (d, *J* = 11.0 Hz, 2H), 5.05 – 4.60 (m, 17H), 4.44 (t, *J* = 12.4 Hz, 3H), 4.23 (td, *J* = 9.7, 3.4 Hz, 2H), 4.14 (t, *J* = 9.6 Hz, 2H), 4.05 (d, *J* = 12.3 Hz, 2H), 3.91 (d, *J* = 5.2 Hz, 3H), 3.74 (dt, *J* = 25.5, 9.1 Hz, 3H), 3.48 (d, *J* = 9.9 Hz, 1H), 3.37 – 3.30 (m, 6H), 3.26 (d, *J* = 9.1 Hz, 1H), 1.61 (s, 4H) 2.34 (s, 1H, OH); ^{13}C NMR (101 MHz, $CDCl_3$) δ 170.20 (C=O), 135.98, 135.88, 135.66, 135.42, 135.35, 133.21, 133.09, 132.87, 132.82, 128.68, 128.43, 128.10, 127.94, 127.91, 127.81, 127.76, 127.64, 127.14, 126.77, 126.61, 126.55, 126.49, 126.38, 126.32, 126.22, 126.11, 125.99, 125.96, 125.93, 125.85, 125.79, 125.73, 98.99 (C-1), 81.45, 80.76, 80.31, 78.01, 77.25, 75.88, 75.04, 74.82, 74.63, 73.35, 72.57, 71.55, 68.16, 53.10, 23.35. ESI-MS (*m*/*z*): $[M+Na]^+$ cald 1385.6 obsd 1385.4, $[M+K]^+$ cald 1402.5 obsd 1402.4.

3,4,6–Tri-O-(2-naphthyl)methyl-2-acetamide-2-deoxy-α-D-glucopyranosyl-(1→6)- 1-O-(1-O-stearoyl-2-O-oleoyl-sn-glycerol)-phosphate-2,3,4,5-tetra-O-(2-naphthyl)methyl -D-myo-inositol **(23)**

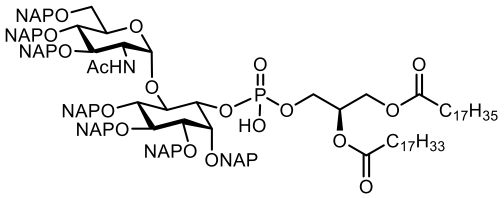

Disaccharide **21a** (0.03 g, 0.022 mmol) and *H*-phosphonate **8** (0.045 g, 0.066 mmol) were dissolved in pyridine (5 mL) and co-evaporated for three times and dried under high vacuum for 2 h. The residue was dissolved in anhydrous pyridine (4 mL) and a solution of pivolyl chloride (0.007 mL, 0.064 mmol) in pyridine (0.5 mL) was added. The solution was stirred for 48 h at room temperature. After 48 h, the reaction mixture was concentrated and redissolved in CH_2Cl_2. To the stirred solution of crude phosphonate in CH_2Cl_2 (5 mL) was added $BrCCl_3$ (0.03 g, 0.088 mmol) and Et_3N (0.03 g, 0.18 mmol) and the reaction was stirred for 3 h. Reaction mixture was concentrated and the product was purified using flash column chromatography on silica gel quenched with Et_3N to obtain lipidated disaccharide **23** (0.03 g, 0.013 mmol, 61%). R_f = 0.55 (MeOH/DCM = 2:9). ^{1}H NMR (400 MHz, $CDCl_3$) δ 7.81 (s, 1H), 7.77 – 7.13 (m, 32H), 7.11 – 6.99 (m, 2H), 6.69 (d, *J* = 8.5 Hz, 1H), 5.59 (s, 1H), 5.31 – 5.12 (m, 5H), 5.06 (q, *J* = 11.7 Hz, 2H), 4.93 (s, 1H), 4.79 (dd, *J* = 24.8, 11.9 Hz, 3H), 4.61 (d, *J* = 10.5 Hz, 1H), 4.54 – 4.33 (m, 2H), 4.09 (ddt, *J* = 45.9, 22.2, 9.7 Hz, 7H), 3.74 – 3.59 (m, 1H), 3.53 – 3.34 (m, 1H), 3.23 (d, *J* = 10.8 Hz, 1H), 3.04 (d, *J* = 10.8 Hz, 1H), 2.85 (dd, *J* = 11.6, 5.7 Hz, 1H), 2.14 (q, *J* = 8.4 Hz, 3H), 1.88 (dq, *J* = 13.7, 6.7 Hz, 3H), 1.42 (d, *J* = 9.5 Hz, 5H), 1.14 (d, *J* = 19.5 Hz, 44H), 0.78 (d, *J* = 7.8 Hz, 6H); ^{13}C NMR ^{13}C NMR (101 MHz, $CDCl_3$) δ 173.4 (C=O), 173.1 (C=O), 136.5, 136.1, 135.8, 135.7, 135.5, 135.2, 133.4, 133.3, 133.3, 133.2, 133.2, 133.1, 133.1, 133.0, 133.0, 132.9, 132.9, 132.7, 130.0, 129.9, 128.3, 128.2, 128.1, 128.1, 128.1, 128.0, 127.9, 127.9, 127.9, 127.8, 127.8, 127.7, 127.7, 126.9, 126.7, 126.6, 126.5, 126.3, 126.3, 126.2, 126.1, 126.1, 126.0, 126.0, 125.9, 125.9, 125.8, 125.7, 125.5, 97.7 (C1), 81.9, 81.0, 80.7, 80.2, 78.2, 75.9, 75.6, 75.3, 75.0, 74.8, 73.4, 72.8, 70.5, 69.7, 67.7, 63.5 (C-6), 62.0, 45.6, 34.2, 34.1, 32.1, 32.0, 29.9, 29.9, 29.8, 29.7, 29.5, 29.5, 29.4, 29.3, 29.3, 29.2, 27.3, 27.2, 24.9, 22.8, 14.3, 8.6. ^{31}P NMR (162 MHz, $CDCl_3$) δ -1.02; MALDI-MS (*m/z*): M_{cald} 2048.617 $[M-H]^-$, M_{obsd} 2048.488.

2-acetamide-2-deoxy-α-D-glucopyranosyl-(1→6)- 1-O-(1-O-stearoyl-2-O-oleoyl-sn-glycerol)-phosphate-D-myo-inositol **(3)**

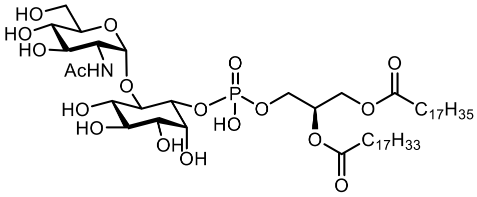

The protected disaccharide **23** (0.02 g, 0.01 mmol) was dissolved in a mixture of TFA/anisole (v/v = 10:2, 3.0 mL) and stirred at 0 ºC for 2 h. The reaction mixture was warmed to room temperature and stirred for additional 3 h. The mixture was diluted with toluene (2 mL) and concentrated. The product was purified by size exclusion chromatography on a Sephadex

LH-20 column (15 x 700 mm) using a mixture of $CHCl_3$/MeOH/H_2O (3:3:1) as eluent to give the pseudodisaccharide **3** (6.3 mg, 0.006 mmol, 60%). ^{1}H NMR (700 MHz, CDC13/MeOD/ D_2O (3:3:1)) δ: 5.30-5.22 (m, 1H, -C<u>H</u>=CH-), 5.11 – 5.00 (m, 1H , -CH=C<u>H</u>-), 4.92 (m, 1H, H-1), 4.22 – 4.08 (m, 1H), 4.04 (m, 1H), 3.94 (d, *J* = 14.7 Hz, 1H), 3.78 (dt, *J* = 20.0, 9.9 Hz, 1H), 3.73 – 3.53 (m, 2H), 3.40 (m, 1H), 3.19 – 2.96 (m, 2H), 2.37 – 2.25 (m, 4H, 2 x CH2), 2.01 (m, 4H, 2x CH_2), 1.74 – 1.48 (m, 7H, 2 x CH_2, -CH_3, Ac), 1.36 – 1.15 (m, 54H (CH_2 lipid)), 0.89 – 0.82 (m, 6H, 2 x CH_3 Lipid). ^{31}P NMR (162 MHz, MeOD) δ 0.09, MALDI-MS (*m*/*z*): M_{cald} 1090.641 [M+Na]$^+$, M_{found} 1090.670.

Synthesis of glycolipid 4

3,4,6–Tri-O-benzyl-2-azido-2-deoxy-α-D-glucopyranosyl-(1→6)-1-O-[1,2-O-(8-methylhexadecanoyl)-sn-glycerol]-phosphate-2,3,4,5-tetra-O-benzyl-D-myo-inositol **(25)**

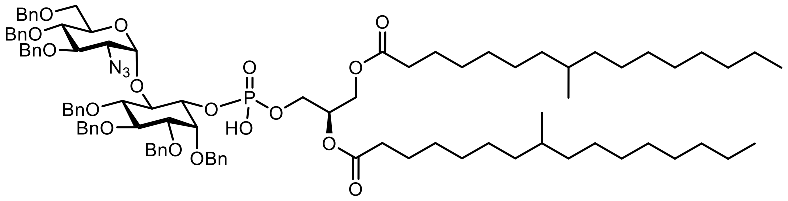

Disaccharide **26**[2] (0.03 g, 0.03 mmol) and *H*-phosphonate **9** (0.06 g, 0.09 mmol) were co-evaporated with pyridine for three times and dried under high vacuum for 2 h. The mixture was dissolved in anhydrous pyridine (5 mL) and a solution of pivolyl chloride (0.01 mL, 0.09 mmol) in pyridine (1 mL) was added. The solution was stirred for 48 h at room temperature. After 48 h, iodine (0.04 g, 0.15 mmol) and water (0.2 mL) were added and reaction was stirred for 4 h. The reaction mixture was quenched with $Na_2S_2O_3$ and extracted with CH_2Cl_2. The organic layer was concentrated and purified with Et_3N quenched flash column chromatography to obtain bilipidated disaccharide **25** (0.03 mg, 0.003 mmol, 55%)**.** R$_f$ = 0.6 (MeOH/DCM = 1:9) ^{1}H NMR (400 MHz, $CDCl_3$) δ 7.43 (dd, *J* = 22.7, 7.9 Hz, 2H), 7.34 (s, 5H), 7.31 (d, *J* = 5.8 Hz, 7H), 7.25 (d, *J* = 12.9 Hz, 8H), 7.06 (s, 2H), 5.25 (dt, *J* = 11.7, 5.8 Hz, 1H), 5.05 – 3.95 (m, 23H), 3.78 – 3.21 (m, 8H), 3.07 – 2.96 (m, 3H), 2.29 (dq, *J* = 12.3, 7.5 Hz, 5H), 1.63 – 1.56 (m, 7H), 1.30 (d, *J* = 7.2 Hz, 43H), 1.12 – 1.06 (m, 7H), 0.91 (t, *J* = 6.6 Hz, 6H), 0.85 (dd, *J* = 6.3, 2.8 Hz, 8H).^{31}P NMR (162 MHz, $CDCl_3$) δ -1.87. MALDI-MS (*m*/*z*): [M+Na]$^+$ cald 1678.9 obsd 1678.4.

2-amino-2-deoxy-α-D-glucopyranosyl-(1→6)-1-O-[1,2-O-(8-methylhexadecanoyl)-sn-glycerol]-phosphate-D-myo-inositol **(4)**

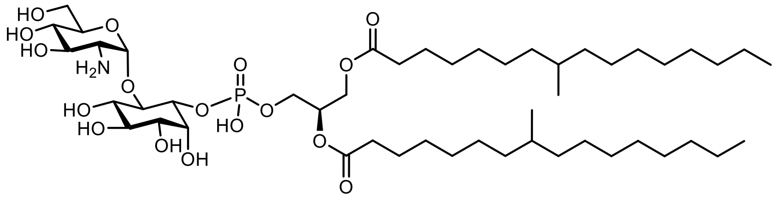

To the stirred solution of pseudodisaccharide **25** (21.0 mg, 0.013 mmol) in mixture of CH_2Cl_2/MeOH/H_2O (3:3:1, 5 mL) at room temperature was added $Pd(OH)_2$ on C (17 mg, 0.024

mmol, 20% Pd content). Hydrogen gas was bubbled through the solution for 15 min and the reaction mixture was stirred under an atmosphere of hydrogen gas for additional 24 h. The $Pd(OH)_2$ was removed by filtration through a pad of celite and the solution was concentrated. The crude product was purified by LH20 size exclusion chromatography with $CH_2Cl_2/MeOH/H_2O$ (3:3:1) as the solvent mixture to obtain the bilipidated disaccharide **4** (6.6 mg, 0.007 mmol, 52%). ^{1}H NMR (400 MHz, MeOD) δ 5.50 (d, *J* = 31.7 Hz, 1H), 5.28 (s, 1H), 4.44 (d, *J* = 11.7 Hz, 3H), 4.26 – 4.16 (m, 3H), 4.09 (s, 3H), 4.04 – 3.91 (m, 2H), 3.84 (d, *J* = 10.6 Hz, 3H), 3.71 (dd, *J* = 14.4, 10.2 Hz, 1H), 3.50 – 3.38 (m, 4H), 3.18 (q, *J* = 7.3 Hz, 2H), 2.35 (dt, *J* = 15.3, 7.5 Hz, 6H), 1.61 (s, 4H), 1.29 (d, *J* = 11.2 Hz, 44H), 1.13 – 1.07 (m, 7H), 0.90 (d, *J* = 6.6 Hz, 6H), 0.85 (d, *J* = 6.5 Hz, 9H). ^{13}C NMR (101 MHz, MeOD) δ 174.30, 174.01, 95.34, 77.84, 77.52, 77.20, 73.05, 72.53, 72.32, 70.97, 70.15, 69.77, 62.79, 54.13, 46.57, 37.02, 34.15, 34.04, 32.69, 31.83, 29.92, 29.68, 29.61, 29.58, 29.26, 29.13, 26.98, 24.90, 24.81, 22.56, 19.48, 19.43, 13.81, 13.77, 8.30, 6.74, -19.42. ^{31}P NMR (162 MHz, MeOD) δ 0.04. ESI-MS (*m/z*): $[M+H]^+$ cald 1000.633 obsd 1000.660.

Biophysical Studies

Table **S1**. Bragg peak and rod positions and the corresponding Full-Widths at Half-Maximum of GPI fragment **2** monolayers on water at different lateral pressures and 20 °C as well as the corresponding lattice parameters.

π, mN/m	Q_{xy}, Å^{-1}	Q_z, Å^{-1}	Q_{xy}, Å^{-1}	Q_z, Å^{-1}
5	1.496<br>0.026	0<br>0.33	1.452<br>0.059	0.508<br>0.33
10	1.502<br>0.032	0<br>0.33	1.466<br>0.050	0.441<br>0.33
15	1.510<br>0.025	0<br>0.31	1.486<br>0.038	0.359<br>0.31
20	1.516<br>0.030	0<br>0.34	1.498<br>0.053	0.273<br>0.34
25	1.522<br>0.022	0<br>0.32	1.514<br>0.034	0.158<br>0.32

π, mN/m	a/b, Å	α/β, °	d	t, °	A_{xy}, Å^2	A_0, Å^2
5	5.049<br>4.900	118.0<br>121.0	0.04019	22.2	21.2	19.6
10	4.990<br>4.871	118.4<br>120.8	0.03260	19.3	20.9	19.7

15	4.909<br>4.831	118.9<br>120.5	0.02148	15.7	20.4	19.7
20	4.863<br>4.805	119.2<br>120.4	0.01599	11.9	20.2	19.7
25	4.801<br>4.775	119.7<br>120.2	0.00704	6.9	19.8	19.7

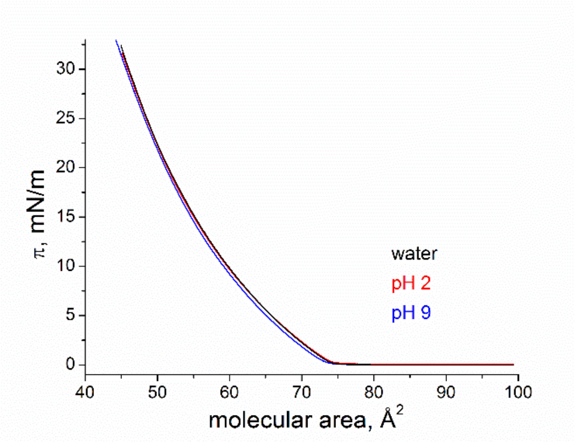

Figure **S1**. Molecular area versus lateral pressure isotherms of GPI fragment 4 monolayers at 20 °C on different subphases (indicated).

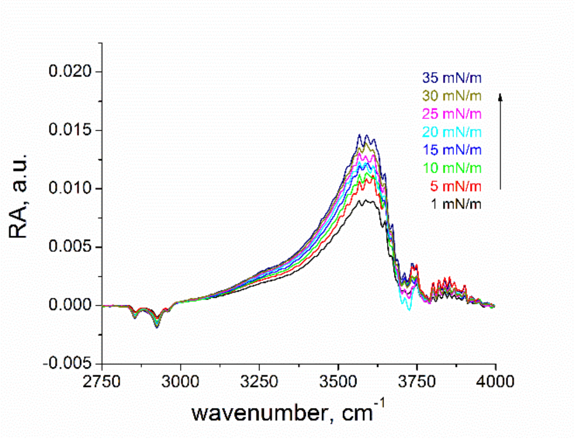

Figure **S2**. Selected part of IRRA spectra along the isotherm (the lateral pressure π is indicated) showing the CH_2 stretching bands and the OH-band of monolayers of GPI fragment **4** on water at 20 °C.

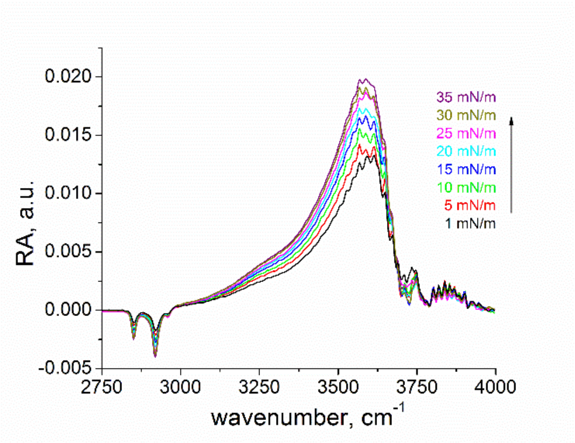

Figure **S3**. Selected part of IRRA spectra along the isotherm (the lateral pressure π is indicated) showing the CH_2 stretching bands and the OH-band of monolayers of GPI fragment **1** on water at 20 °C.

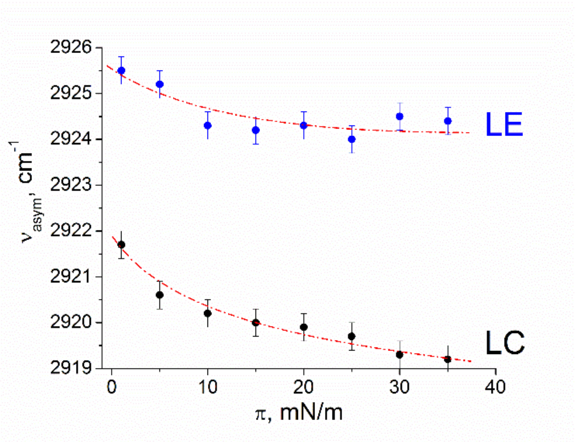

Figure **S4**. Wavenumbers of the asymmetric CH_2 stretching vibration versus the lateral pressure of monolayers of GPI fragments **1** (black) and **4** (blue) on water at 20 °C.

NMR Spectra

^{1}H NMR spectrum of glycerol **12**

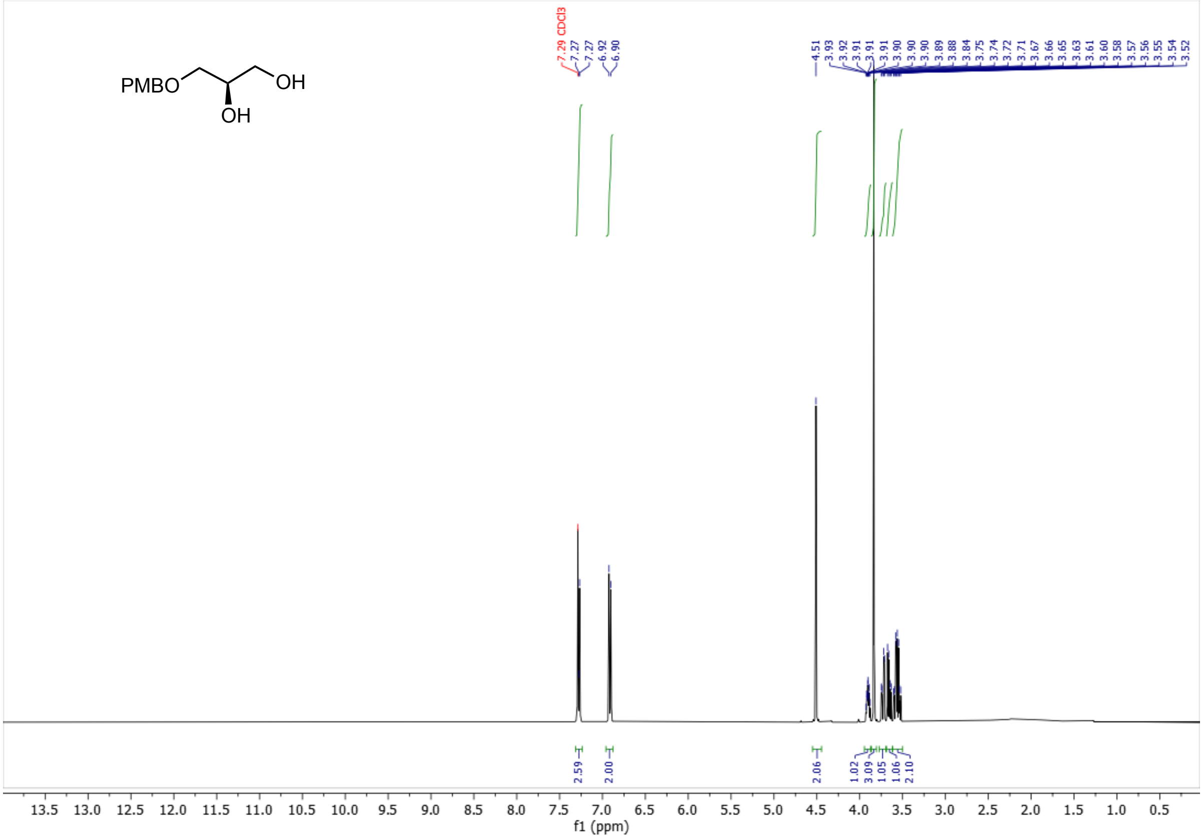

^{13}C NMR spectrum of glycerol **12**

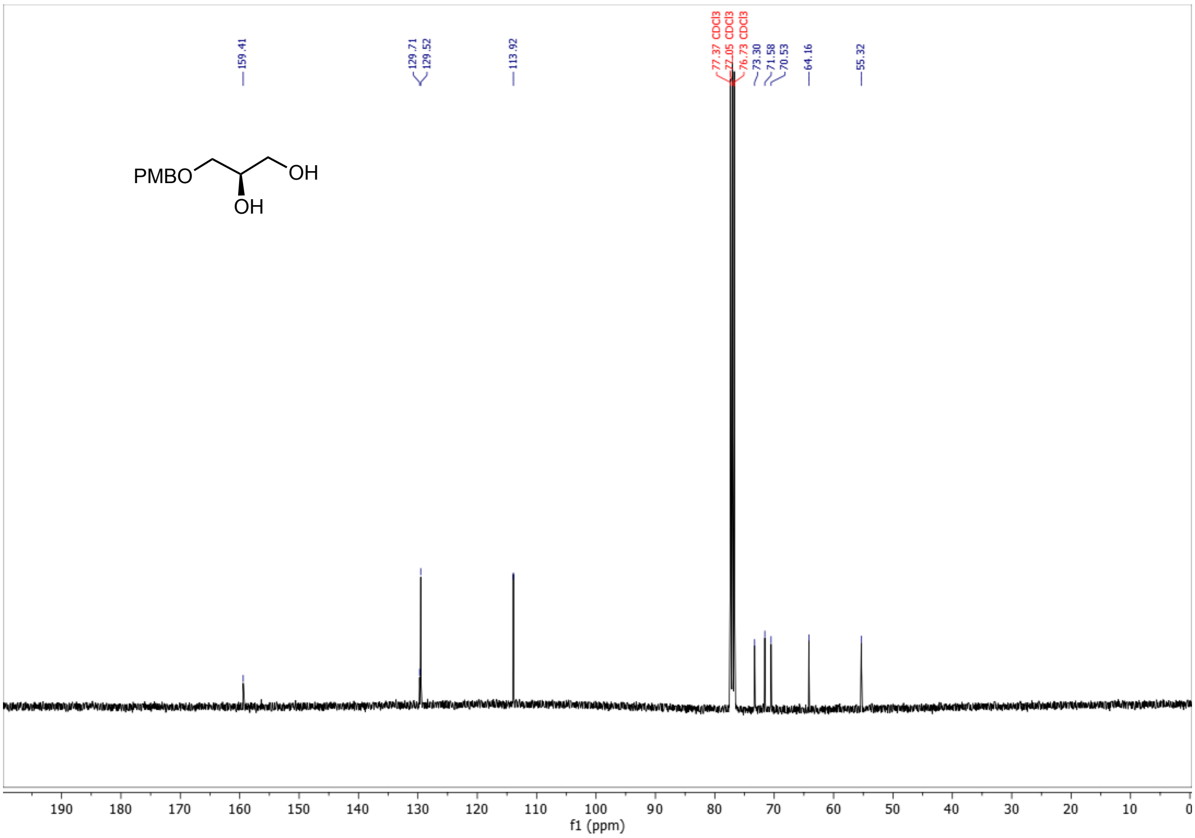

^{1}H NMR spectrum of diacylglycerol **13**

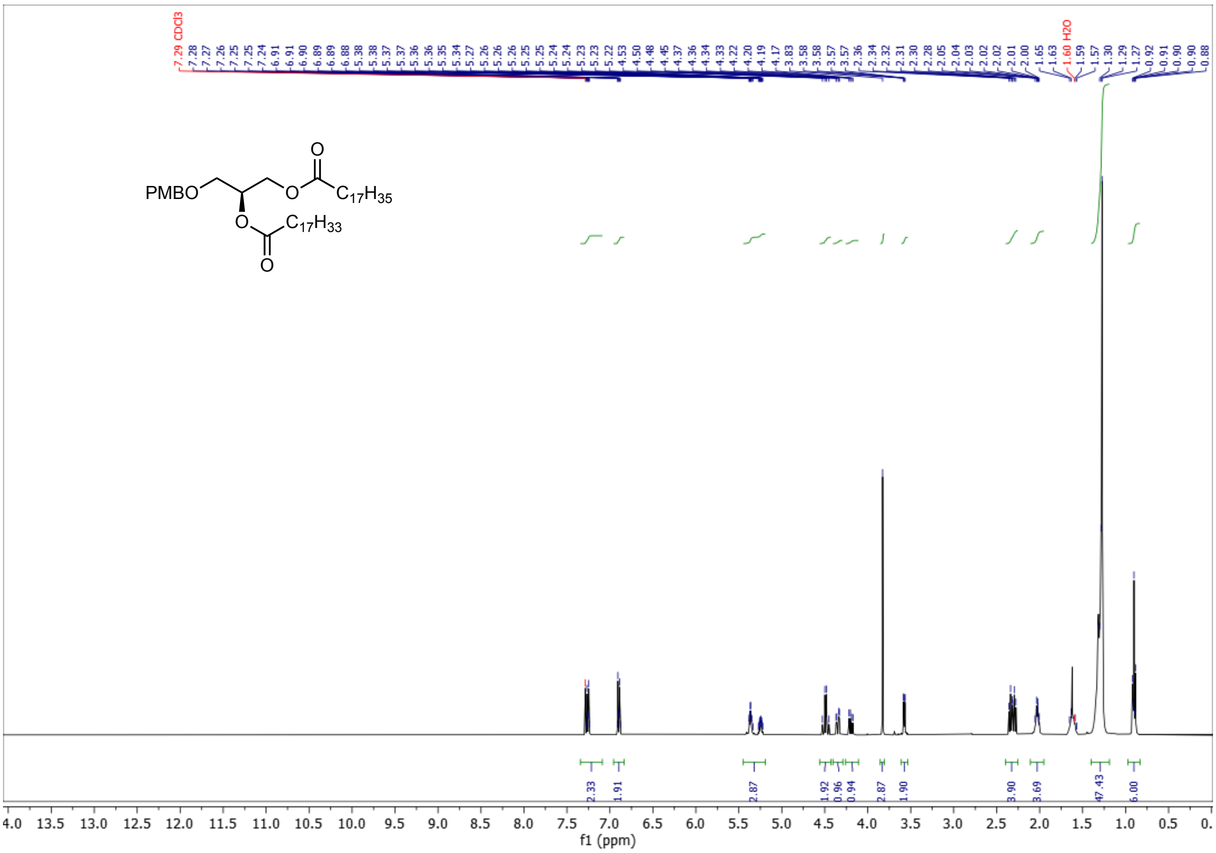

^{13}C NMR spectrum of diacylglycerol **13**

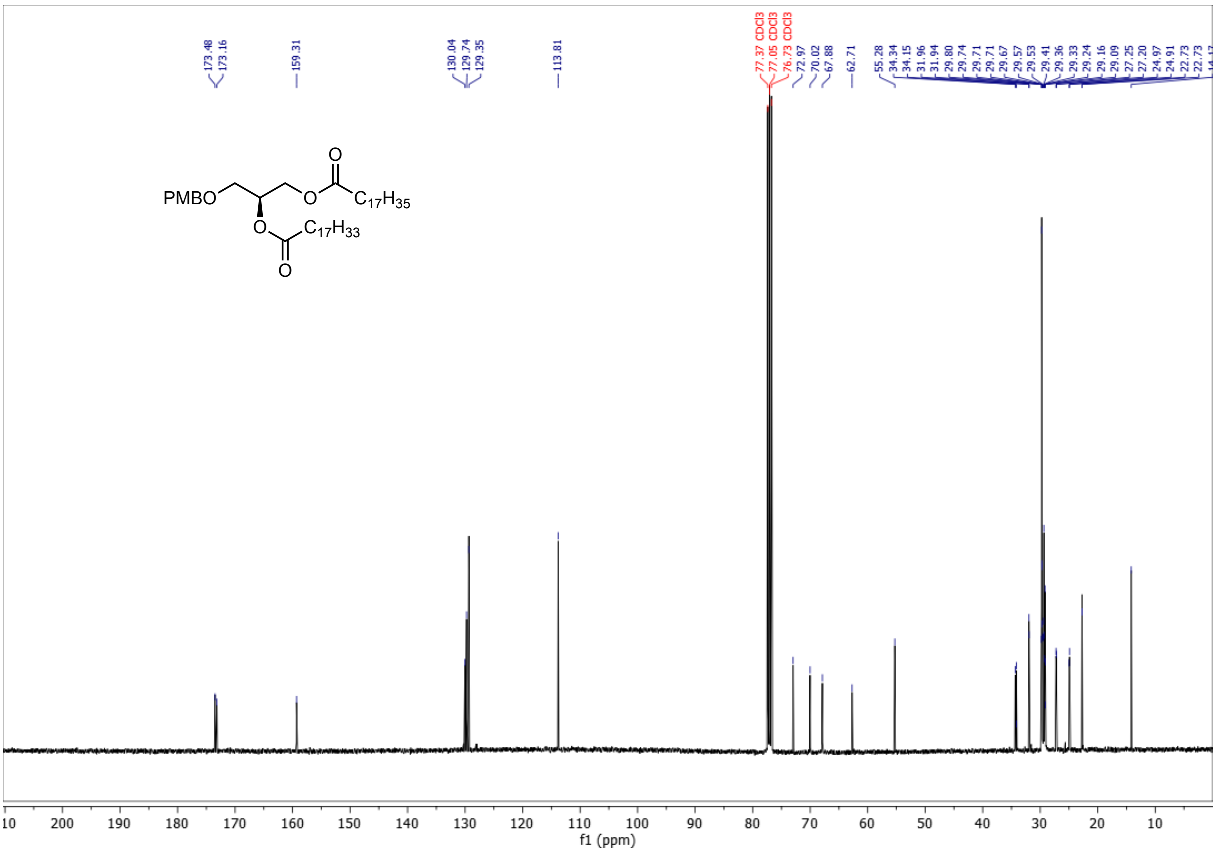

^{1}H NMR spectrum of diacylglycerol **14**

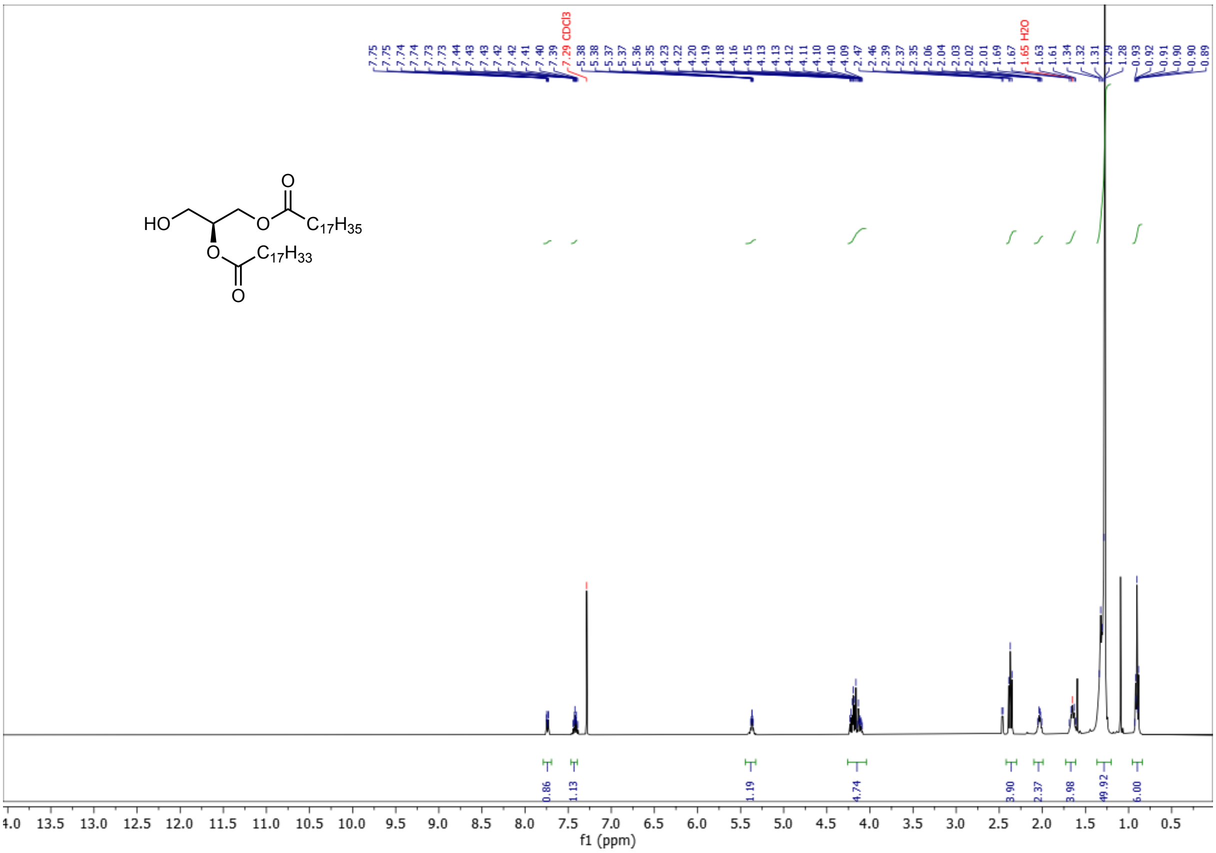

^{13}C NMR spectrum of diacylglycerol **14**

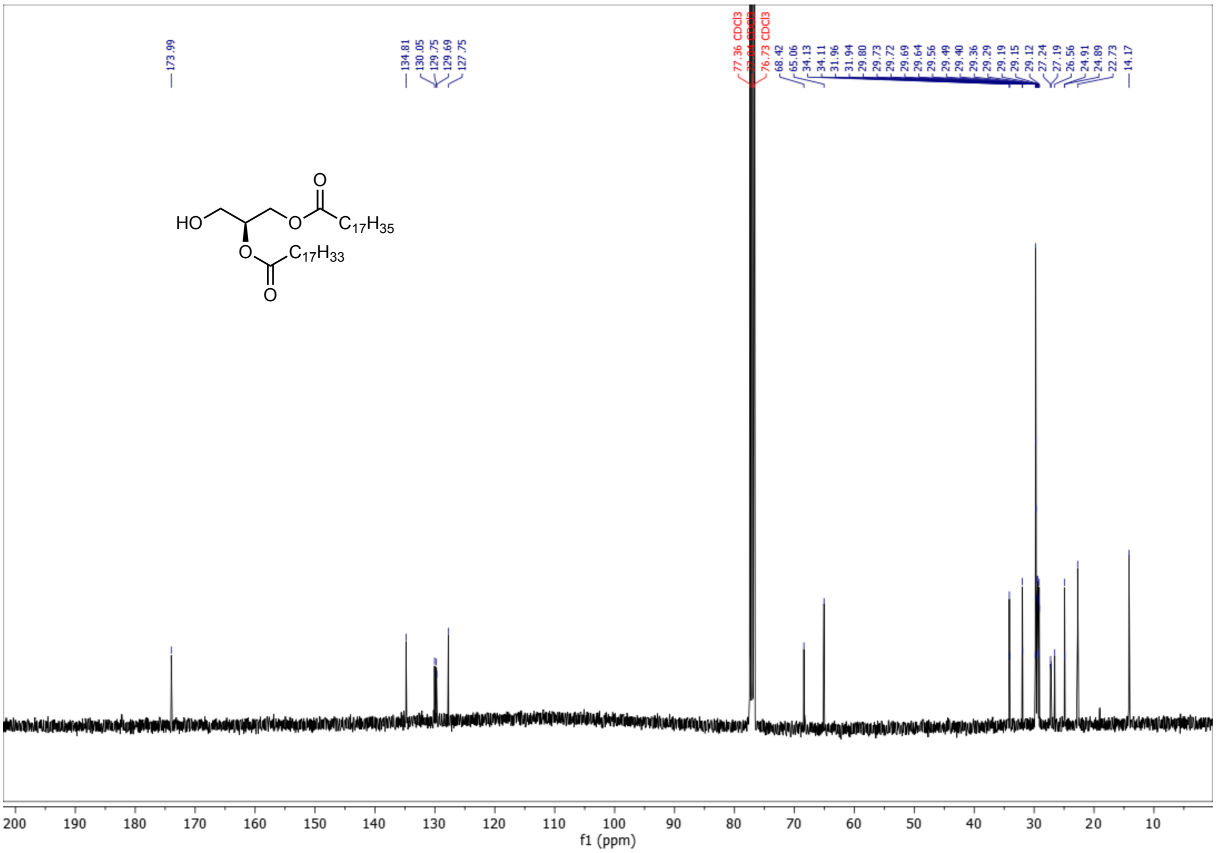

^{1}H NMR spectrum of H-phosphonate **8**

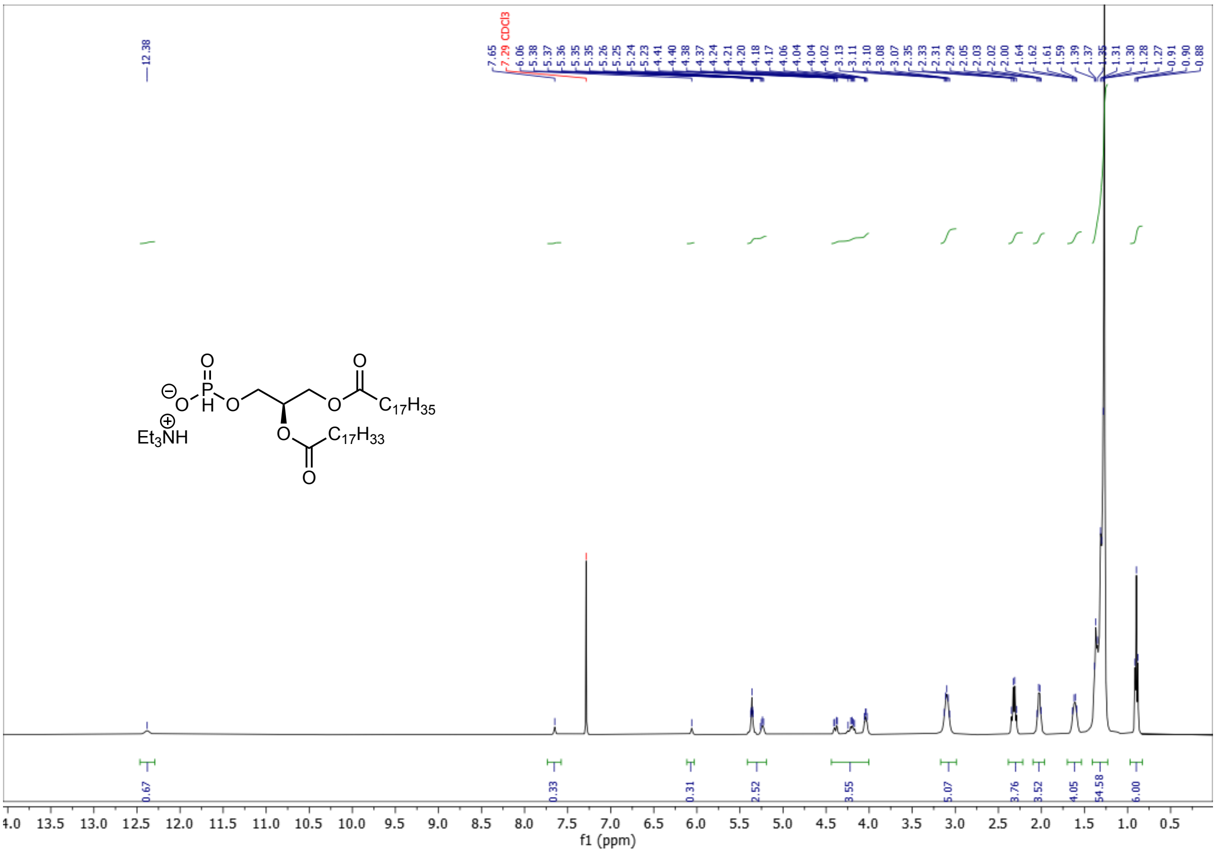

^{13}C NMR spectrum of H-phosphonate **8**

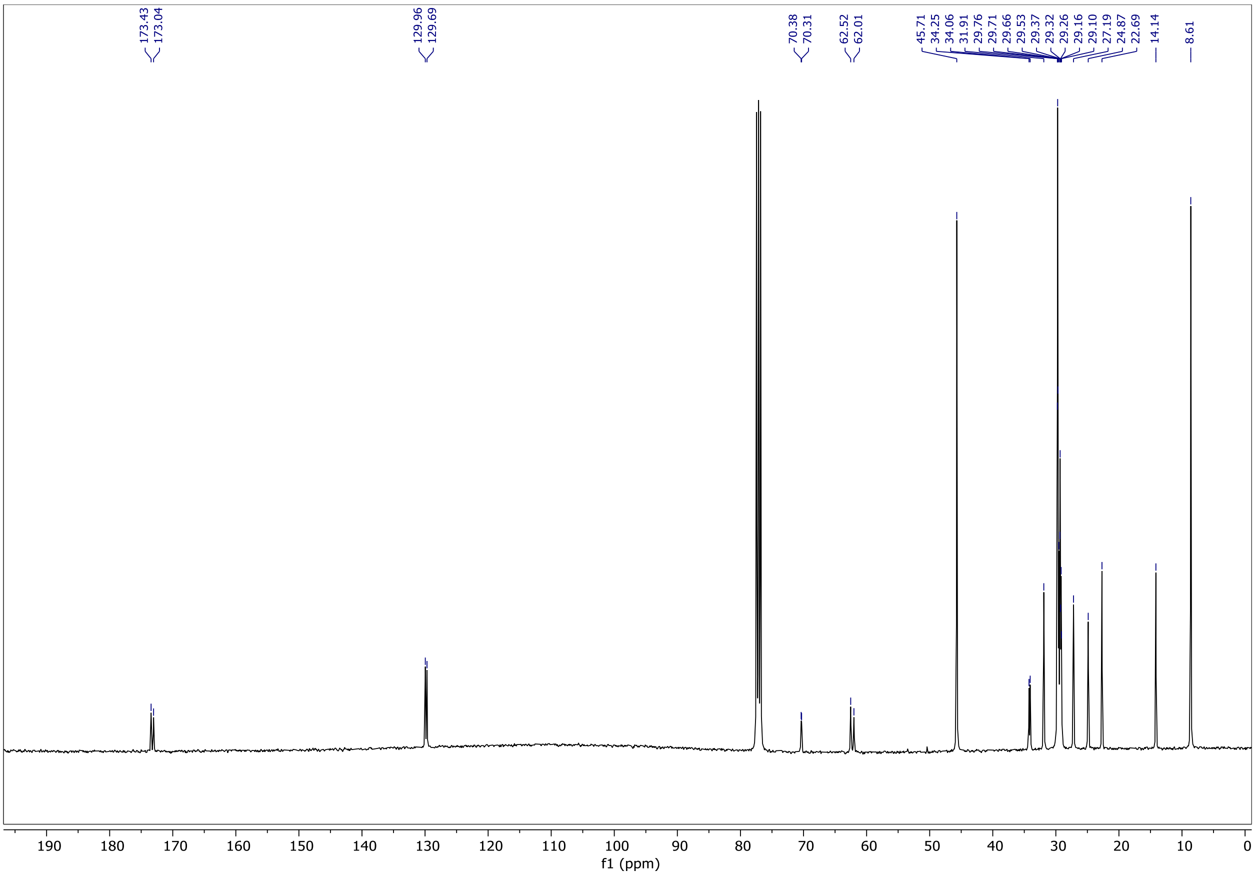

^{31}P NMR spectrum of H-phosphonate **8**

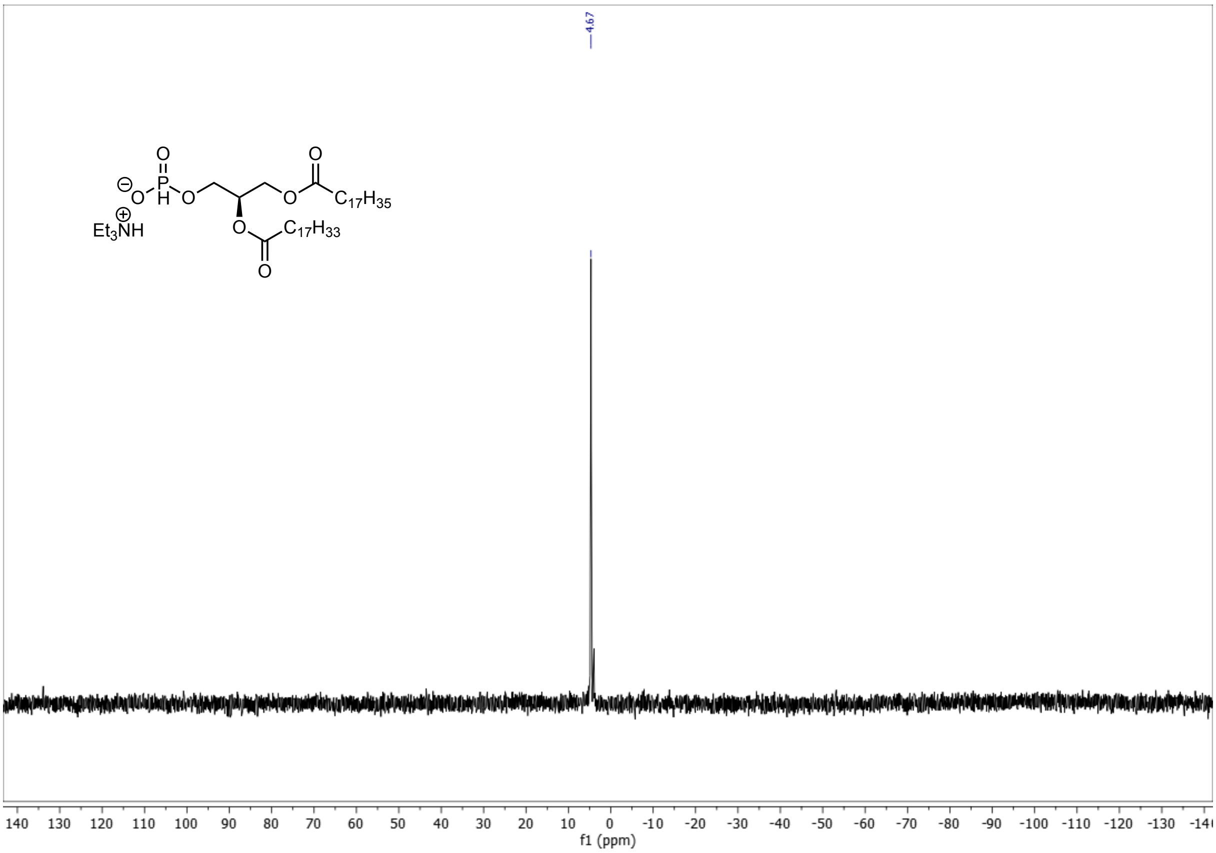

^{1}H NMR spectrum of phosphonium salt **16**

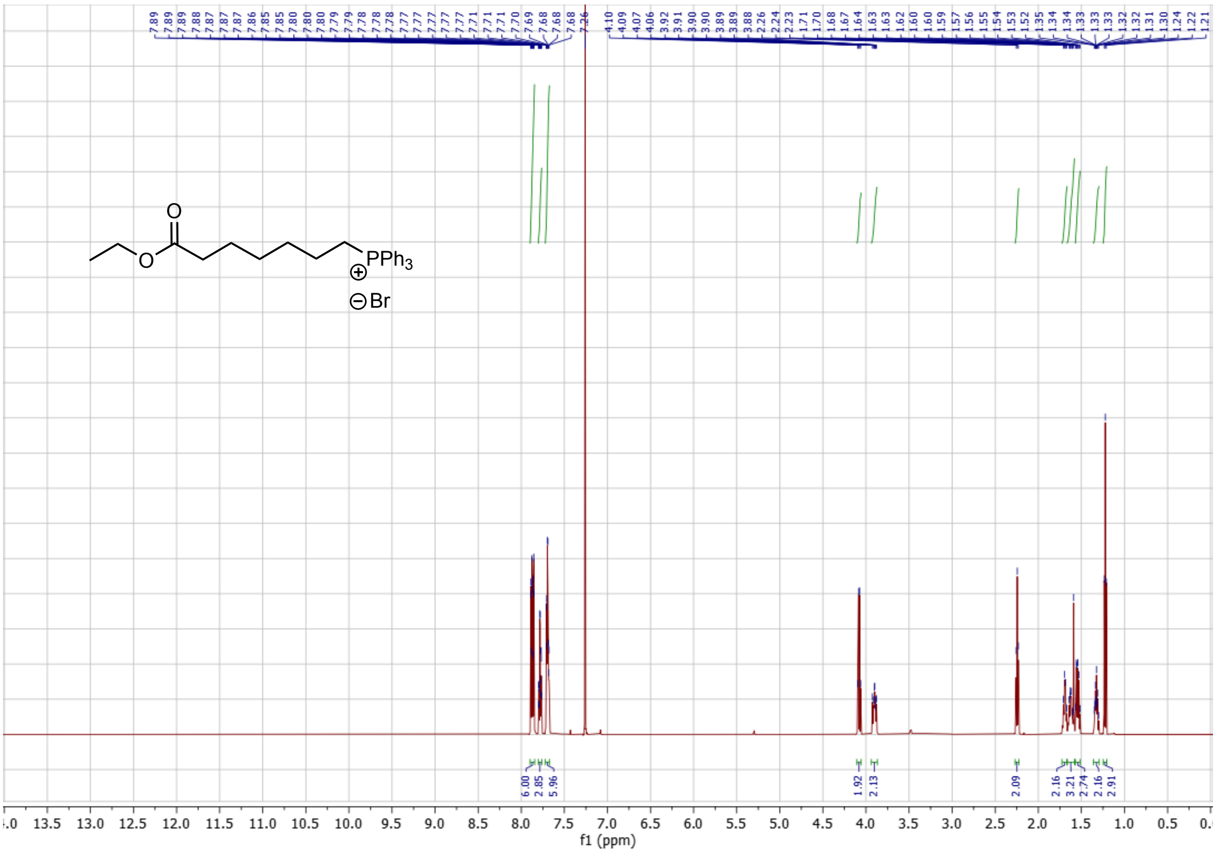

^{1}H NMR spectrum of ethyl 8-methyl-7-en-hexadecanoate **17**

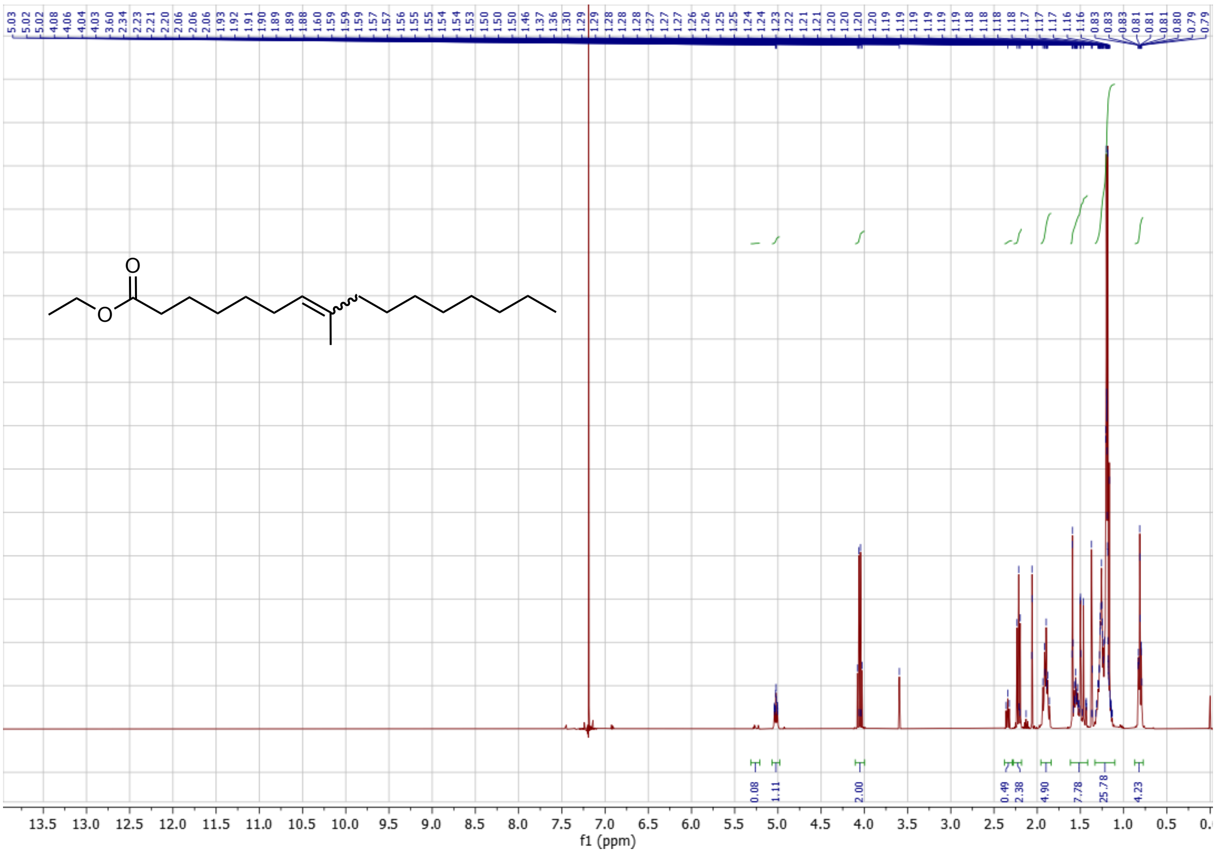

^{1}H NMR spectrum of 8-methyl-hexadecanoic acid **18**

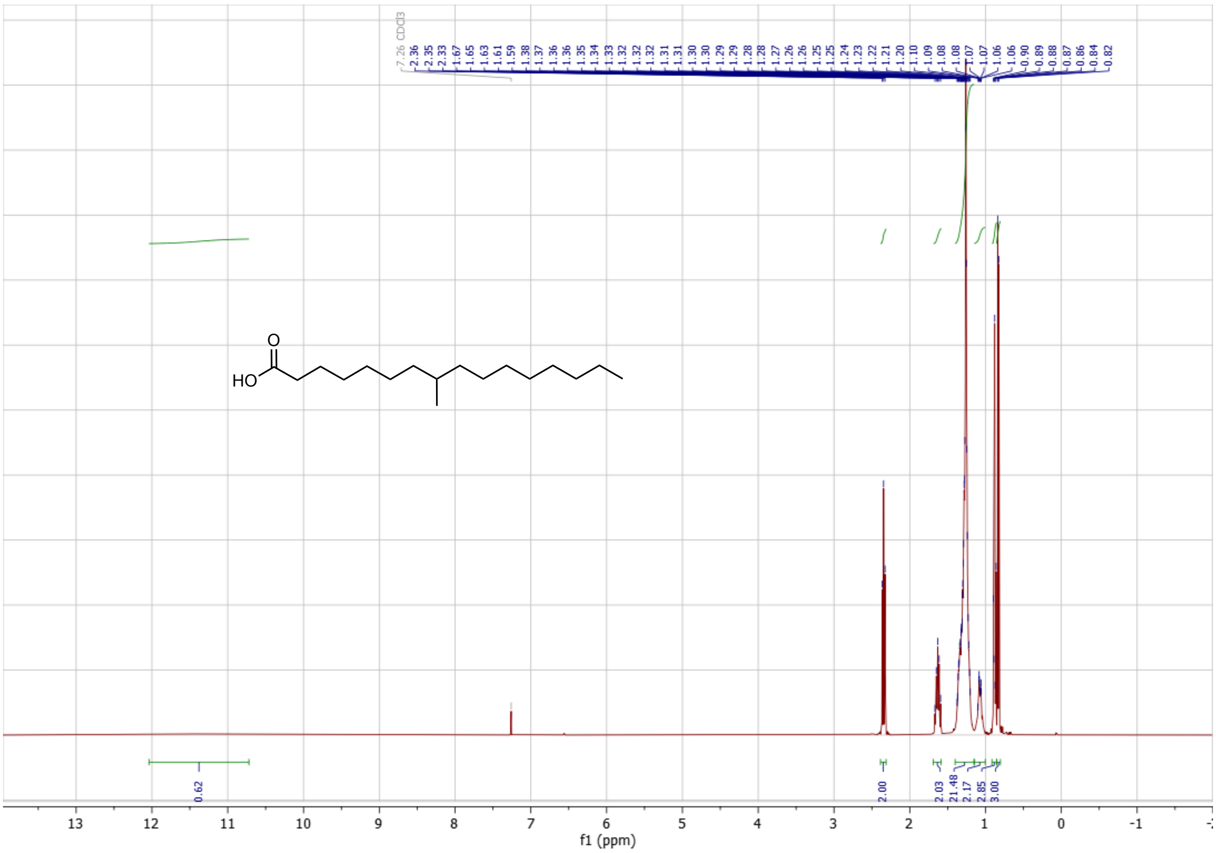

^{13}C NMR spectrum of 8-methyl-hexadecanoic acid **18**

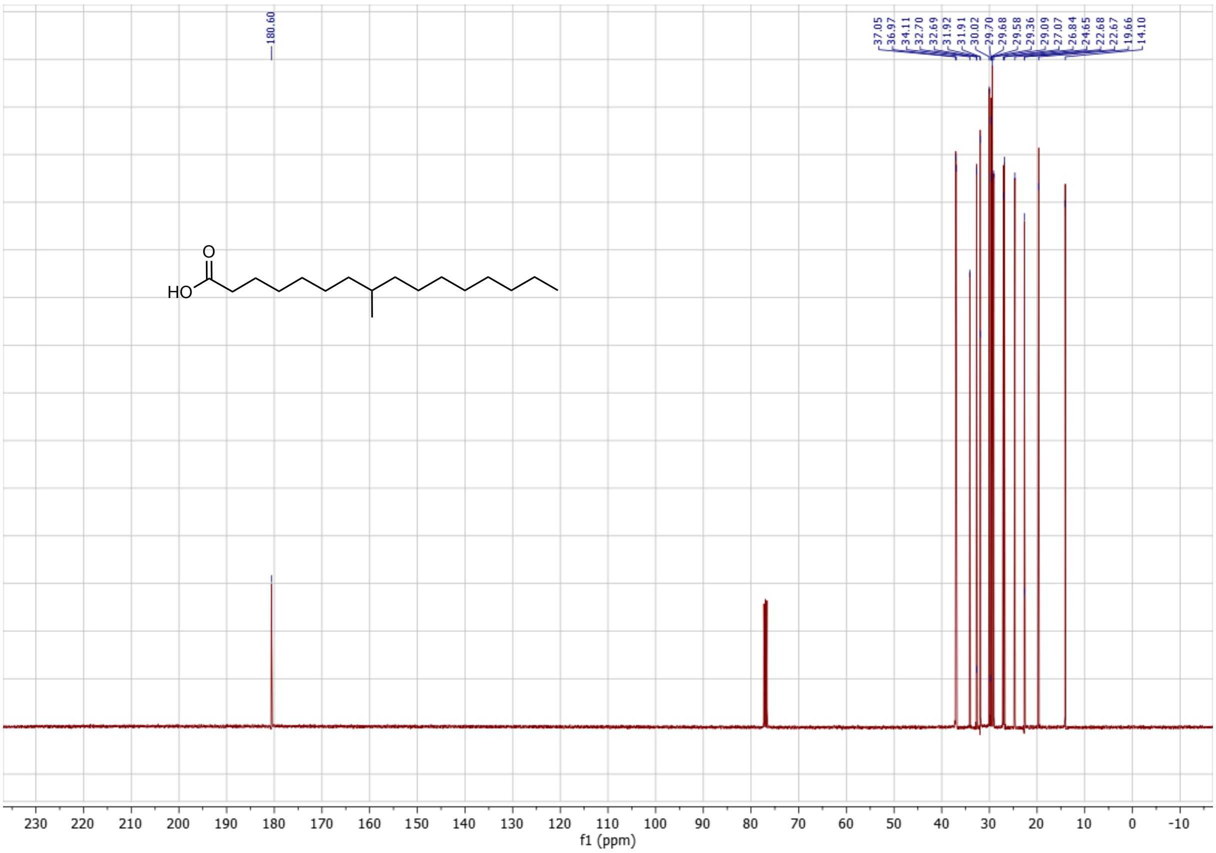

^{1}H NMR spectrum of diacylglycerol **19**

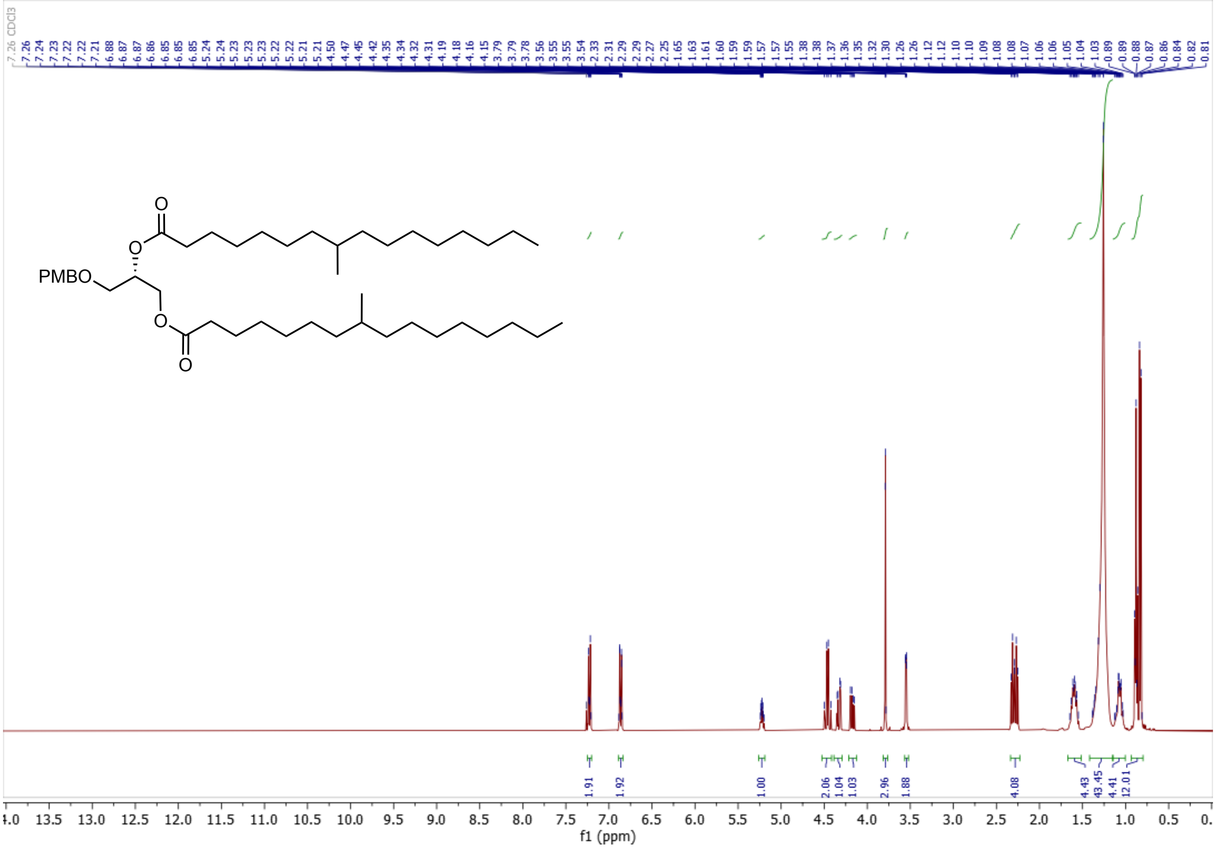

^{13}C NMR spectrum of diacylglycerol **19**

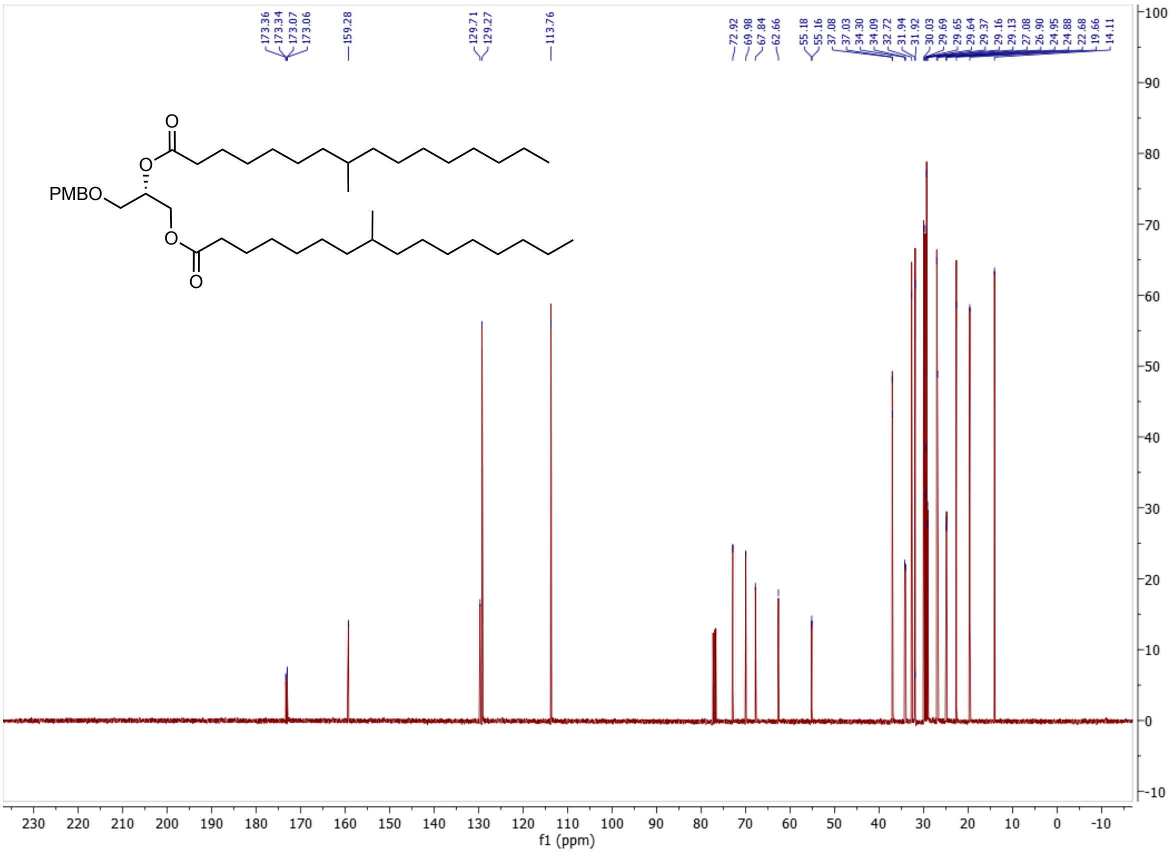

^{1}H NMR spectrum of H-phosphonate **9**

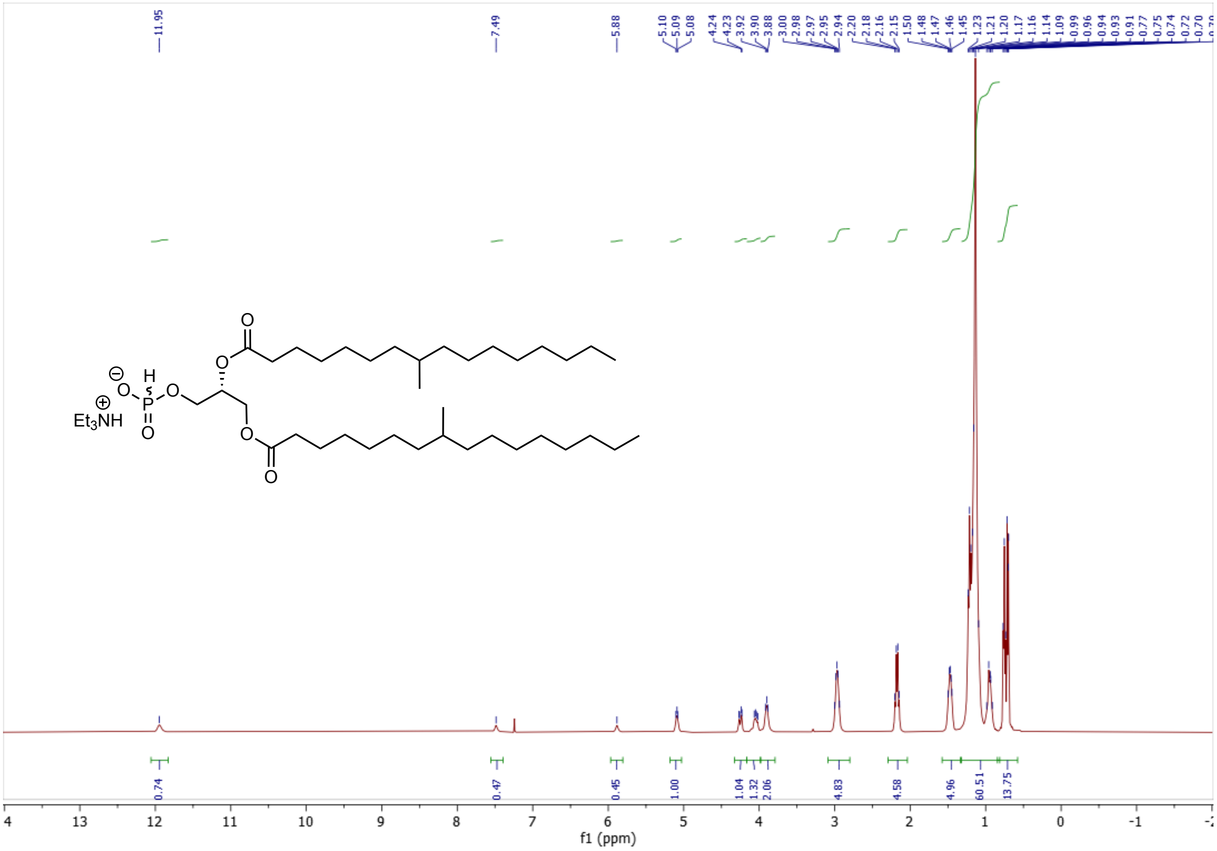

^{13}C NMR spectrum of H-phosphonate **9**

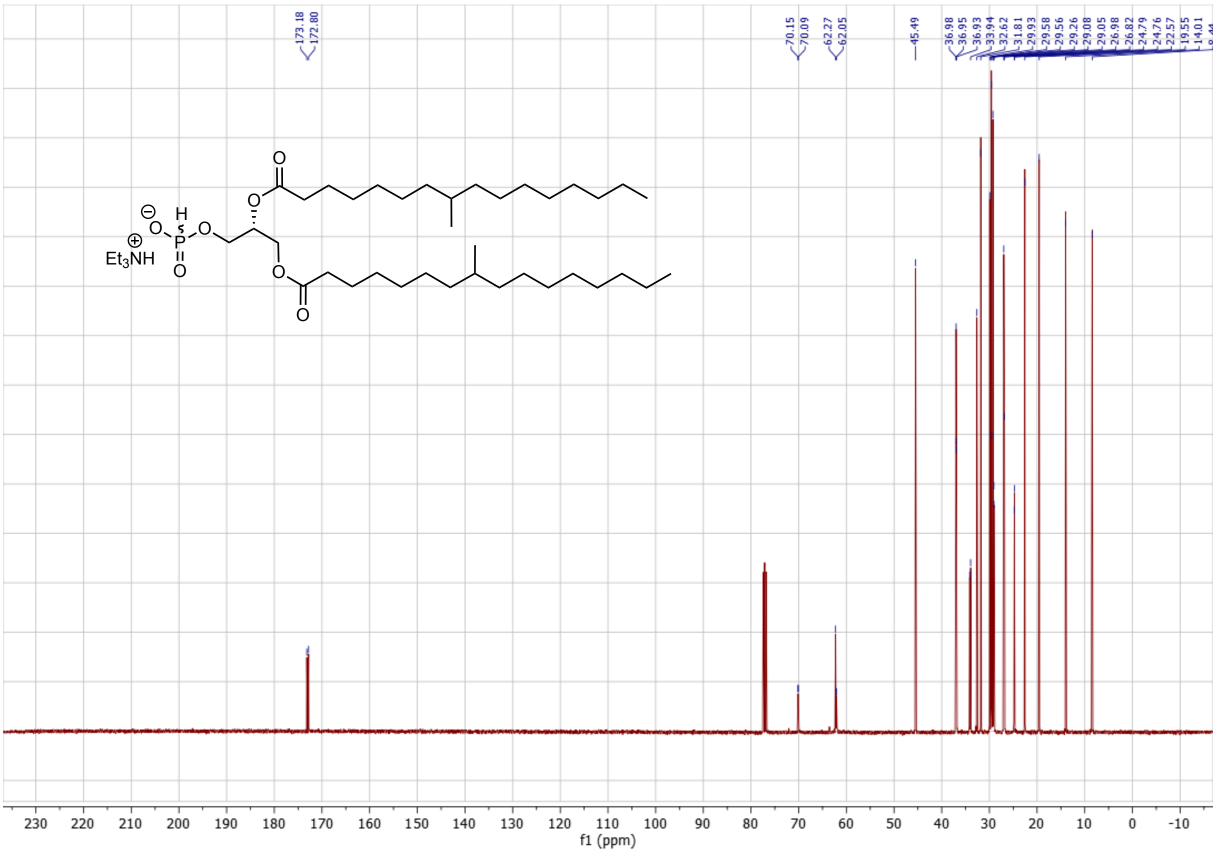

^{31}P NMR spectrum of H-phosphonate **9**

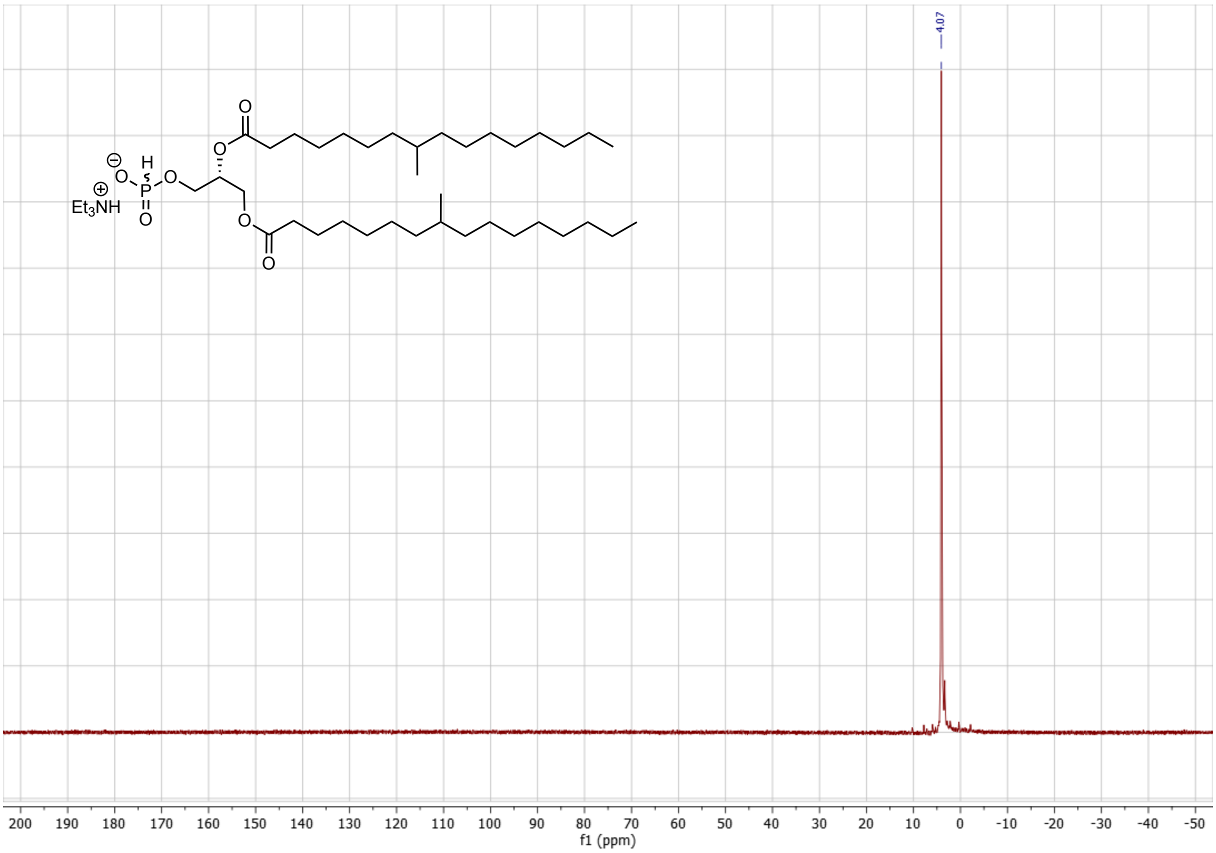

^{1}H NMR spectrum of pseudodisaccharide **20**

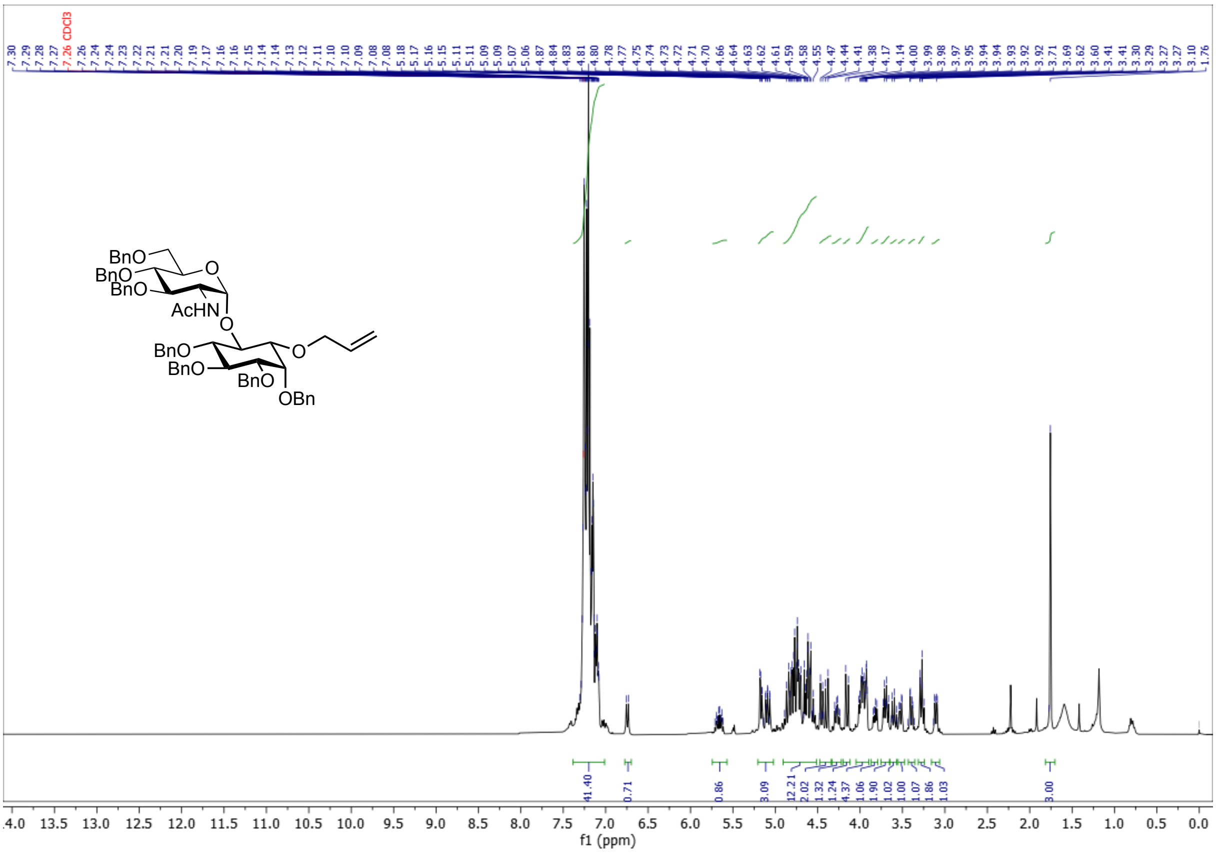

^{13}C NMR spectrum of pseudodisaccharide **20**

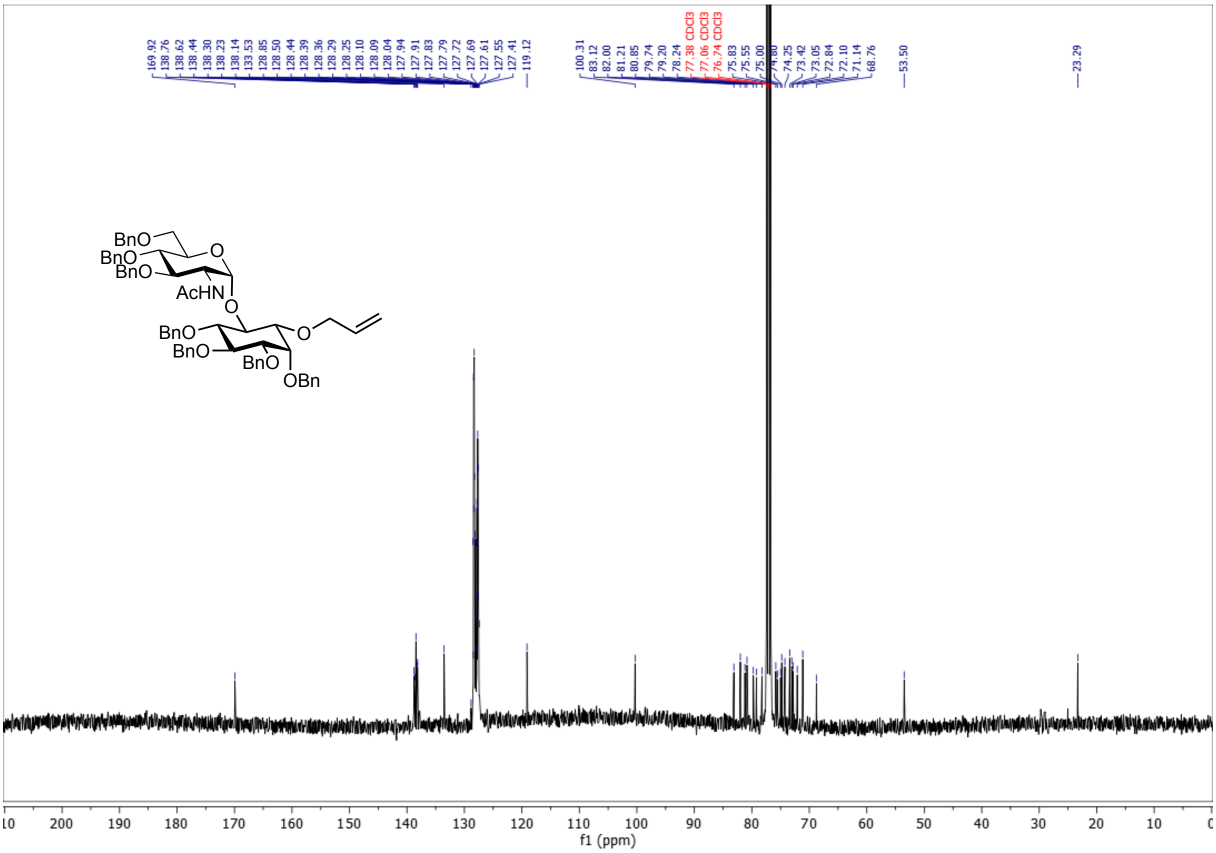

HSQC NMR spectrum of pseudodisaccharide **20**

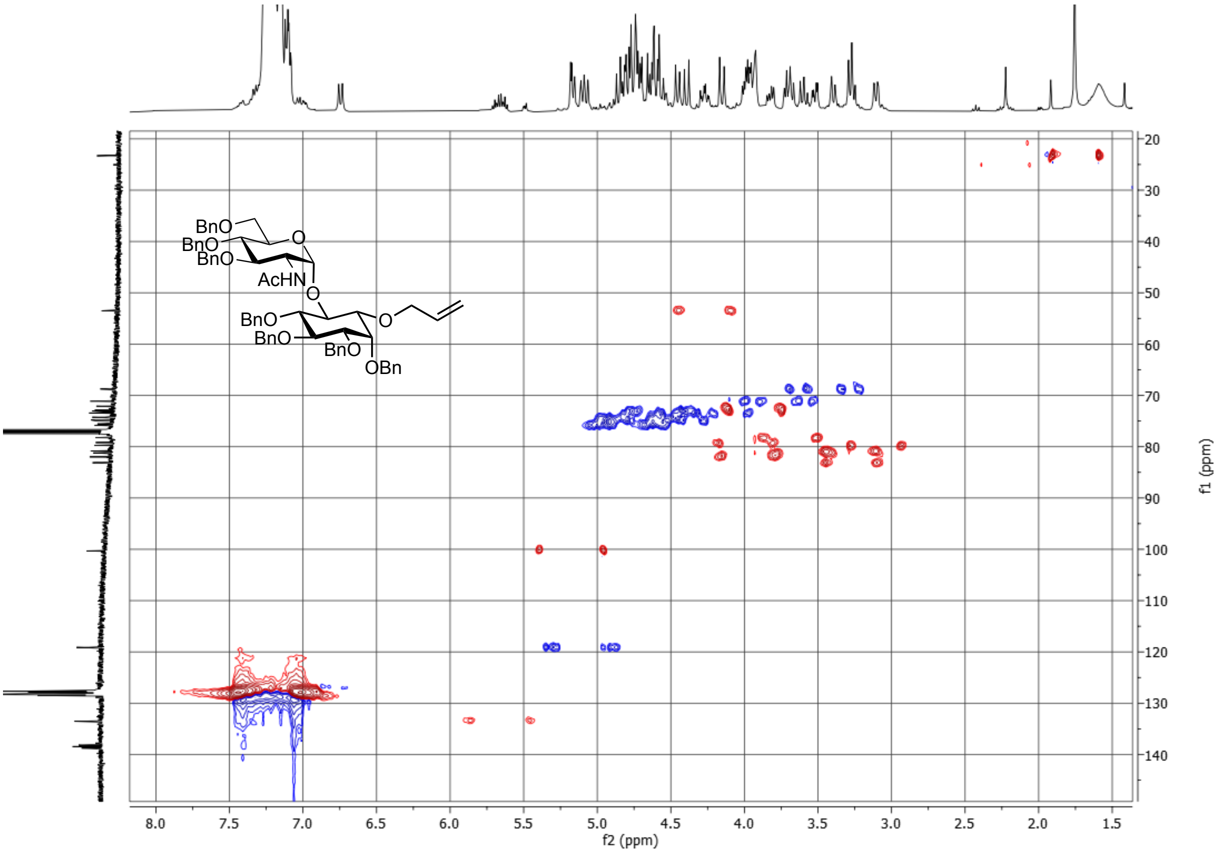

^{1}H NMR spectrum of protected glycolipid **22**

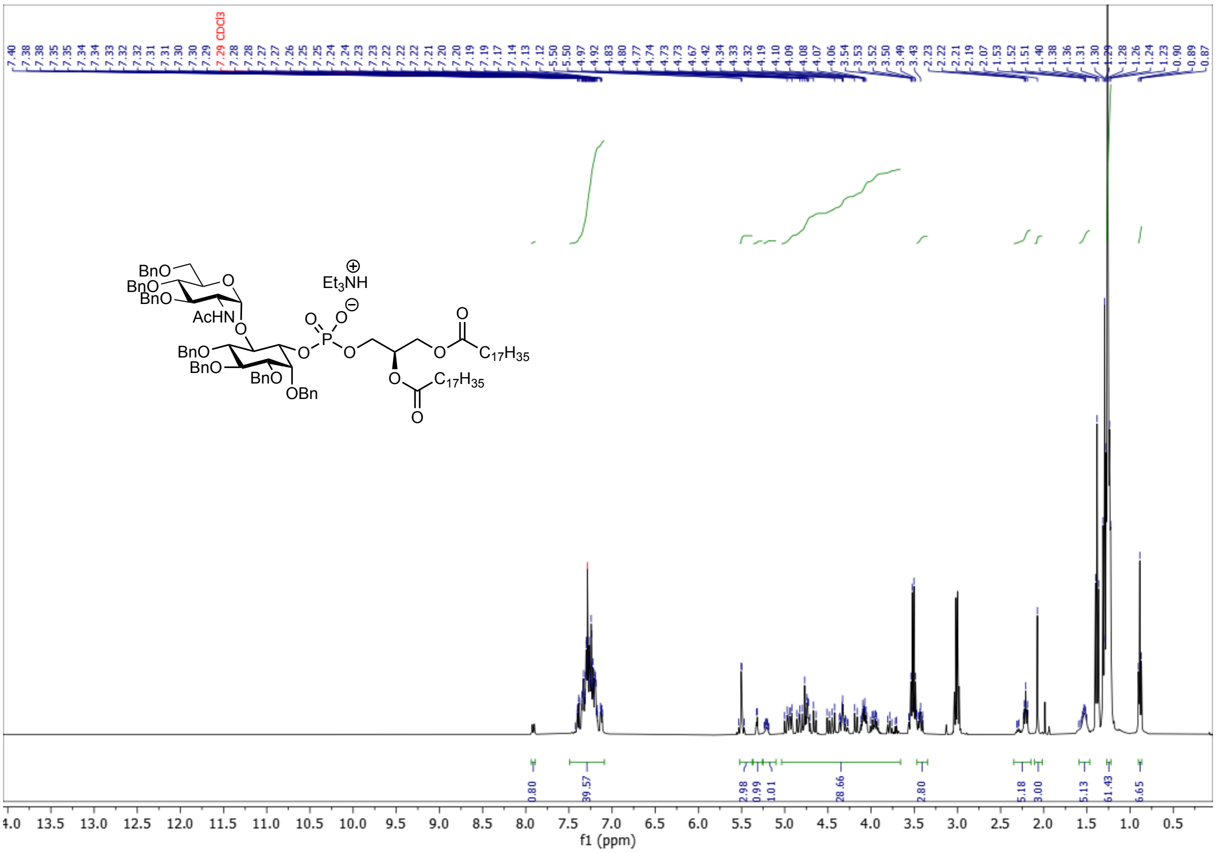

^{13}C-NMR spectrum of protected glycolipid **22**

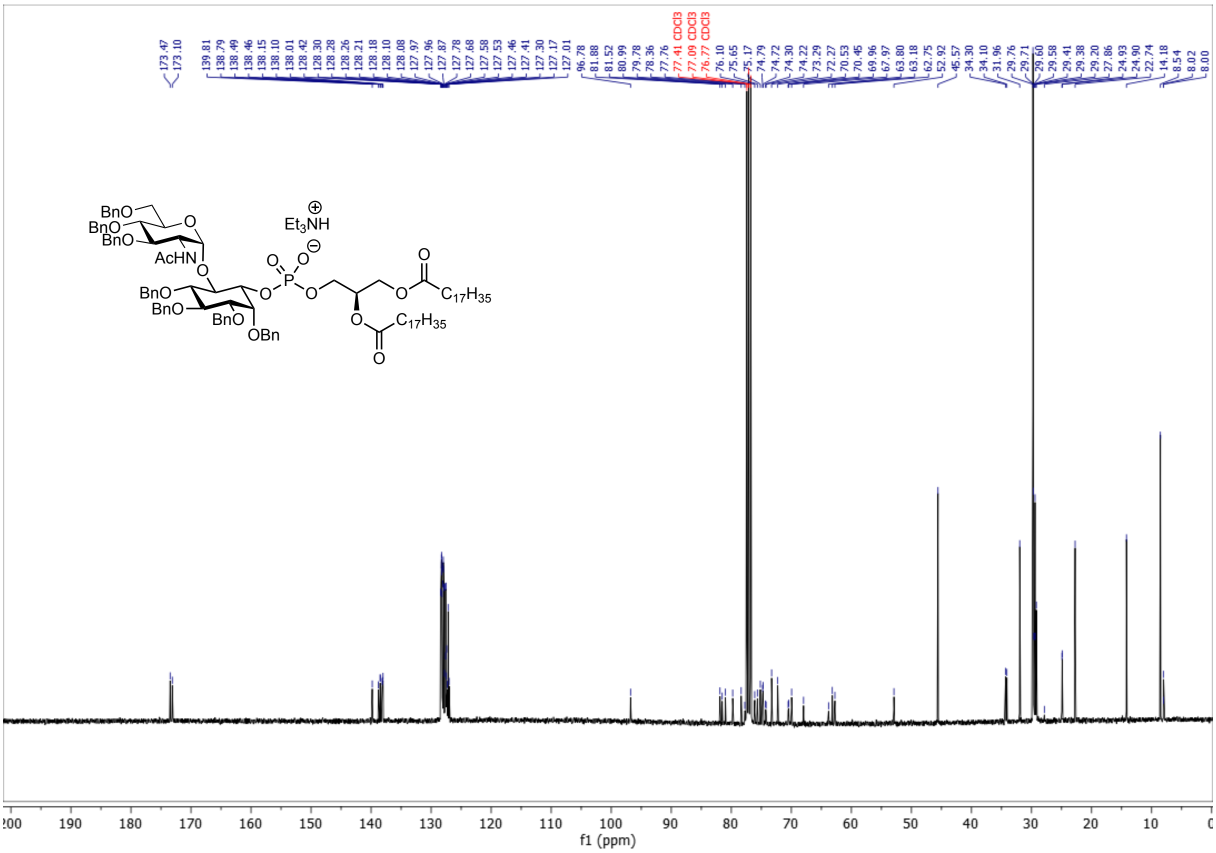

^{31}P-NMR spectrum of protected glycolipid **22**

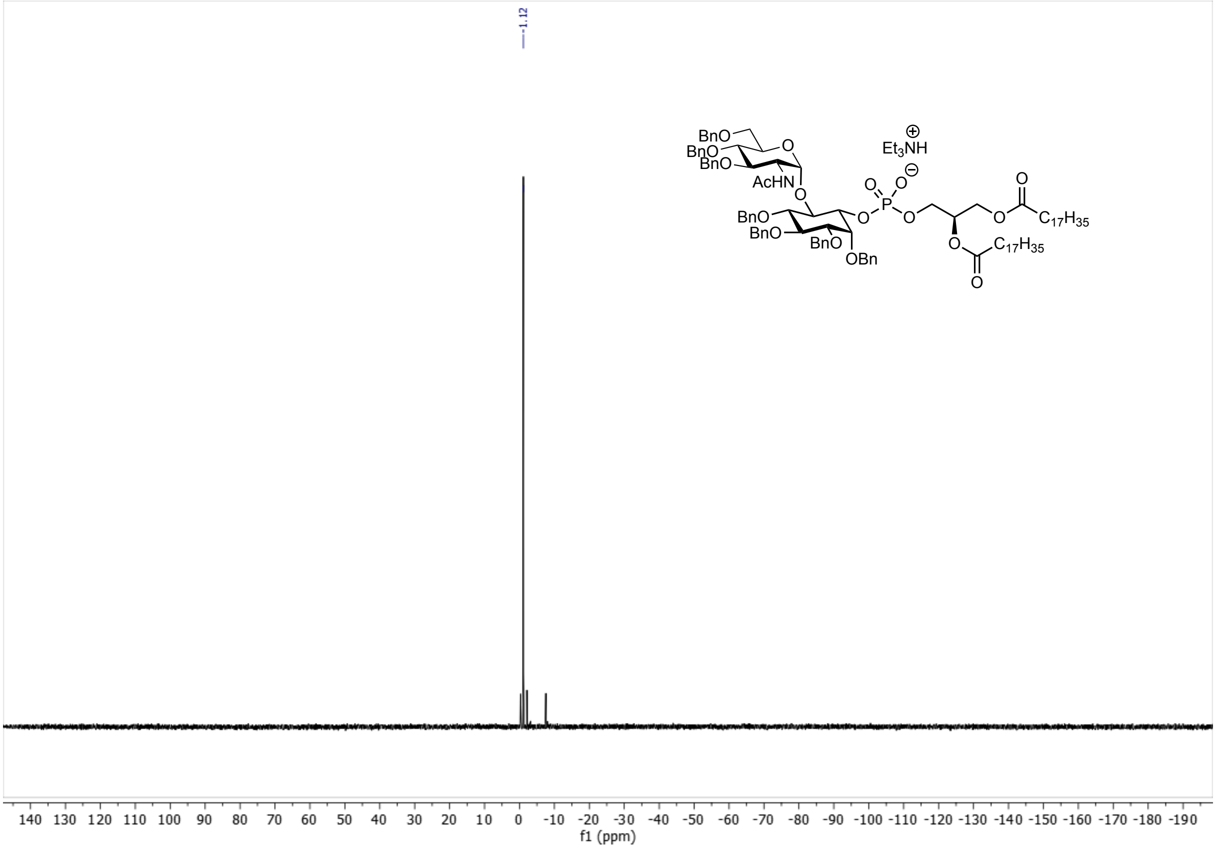

^{1}H-NMR spectrum of glycolipid **2**

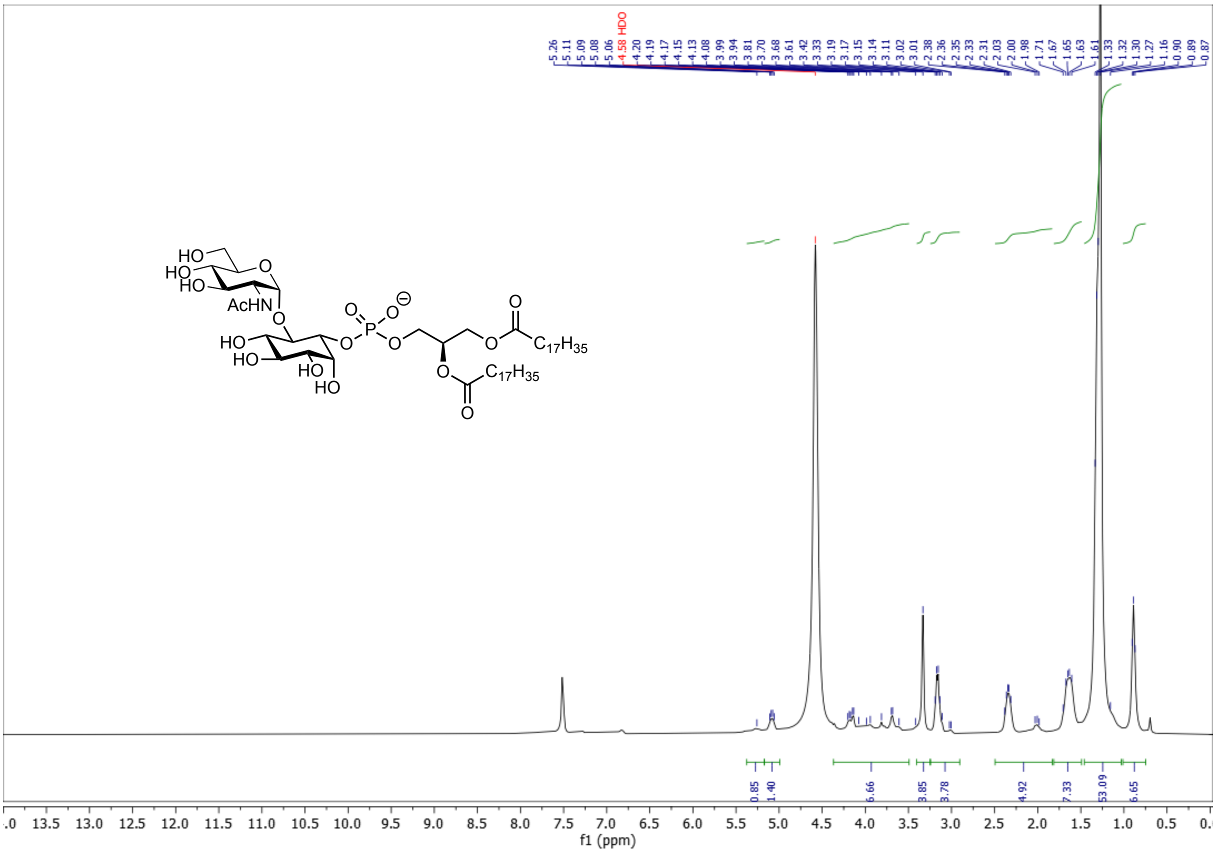

^{31}P-NMR spectrum of glycolipid **2**

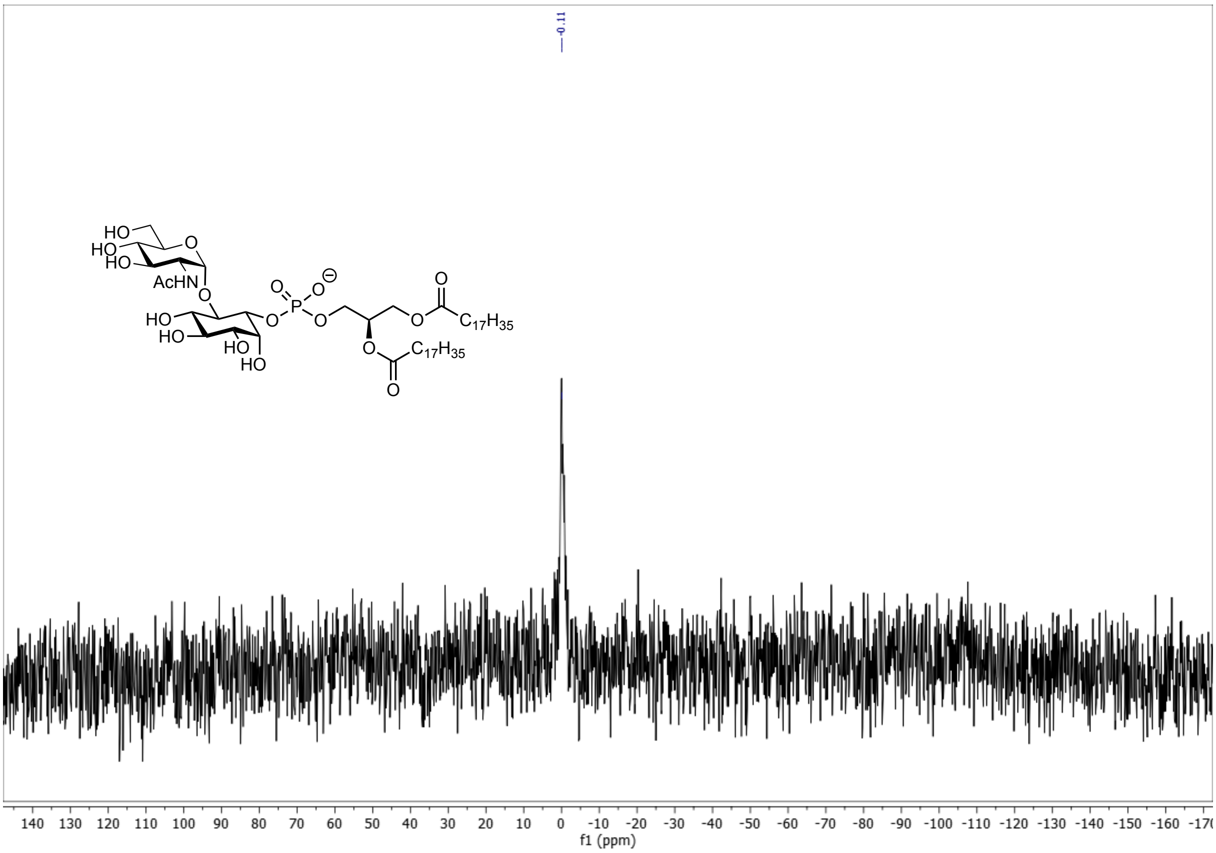

^{1}H-NMR spectrum of pseudodisaccharide **21**

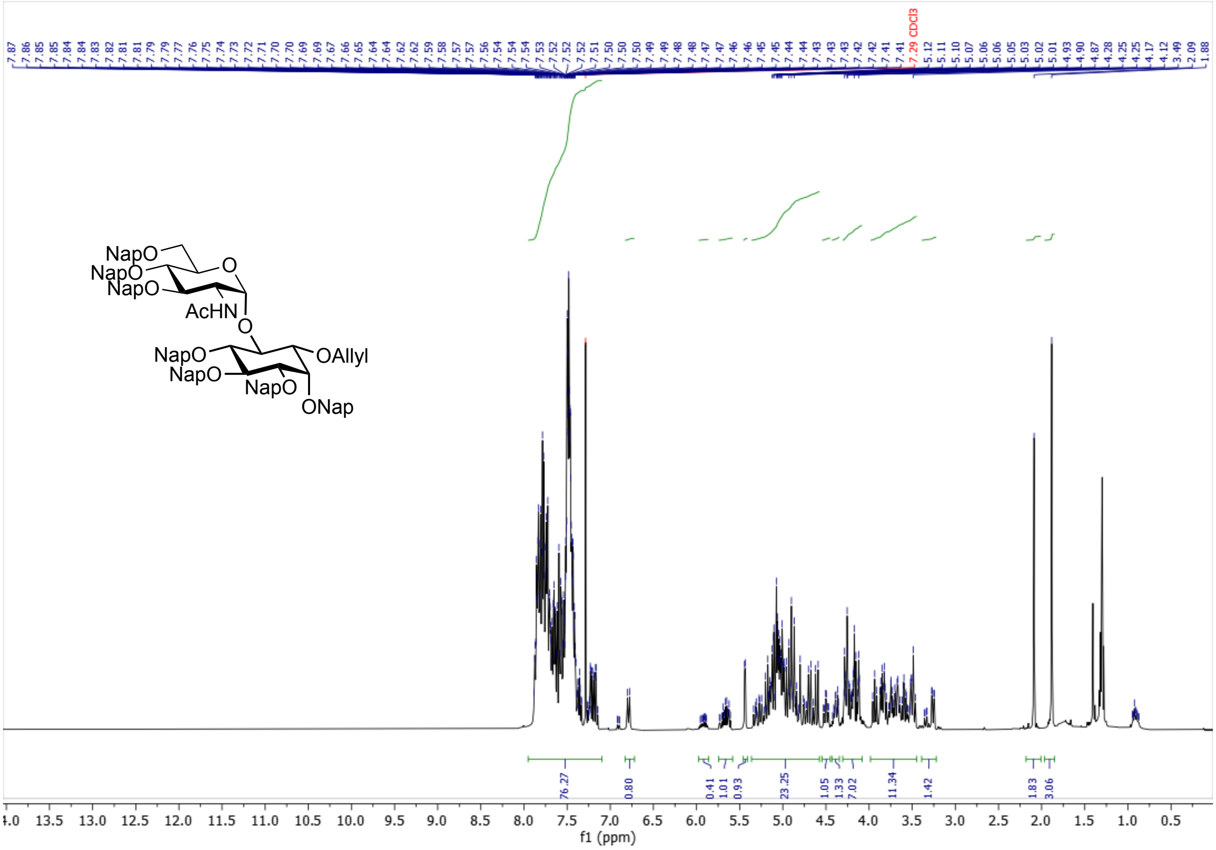

^{13}C-NMR spectrum of pseudodisaccharide **21**

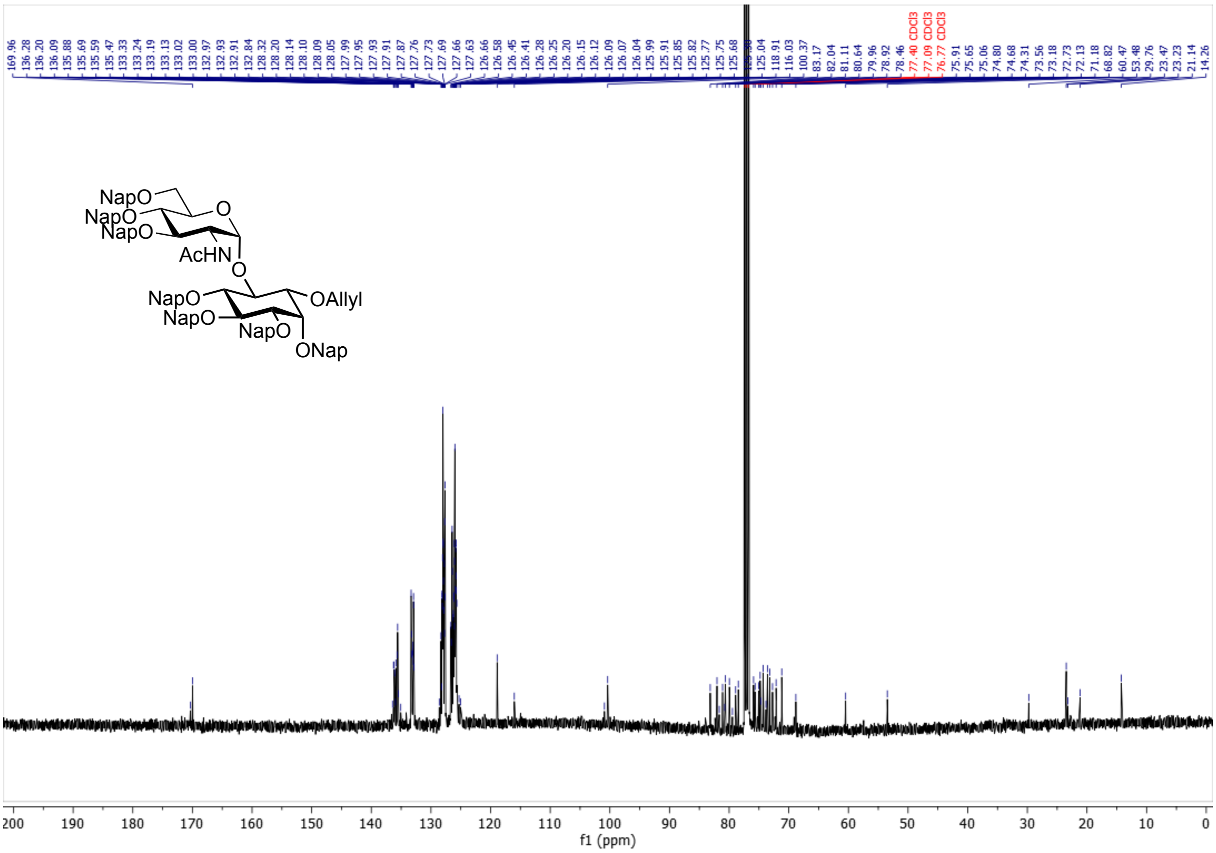

HSQC-NMR pseudodisaccharide **21**

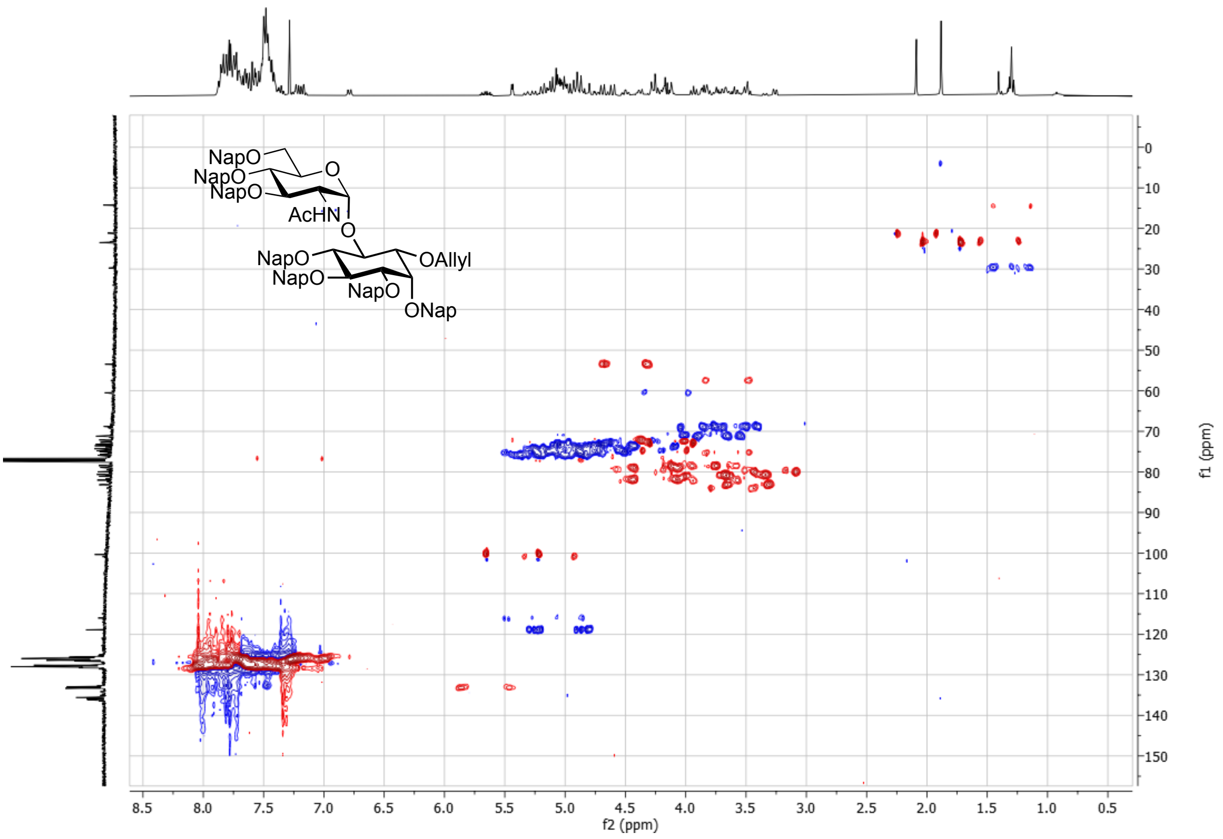
NapO
NapO
NapO
O
AcHN
O
NapO
NapO
NapO
OAllyl
ONap
f2 (ppm)
f1 (ppm)

^{1}H-NMR pseudodisaccharide **21a**

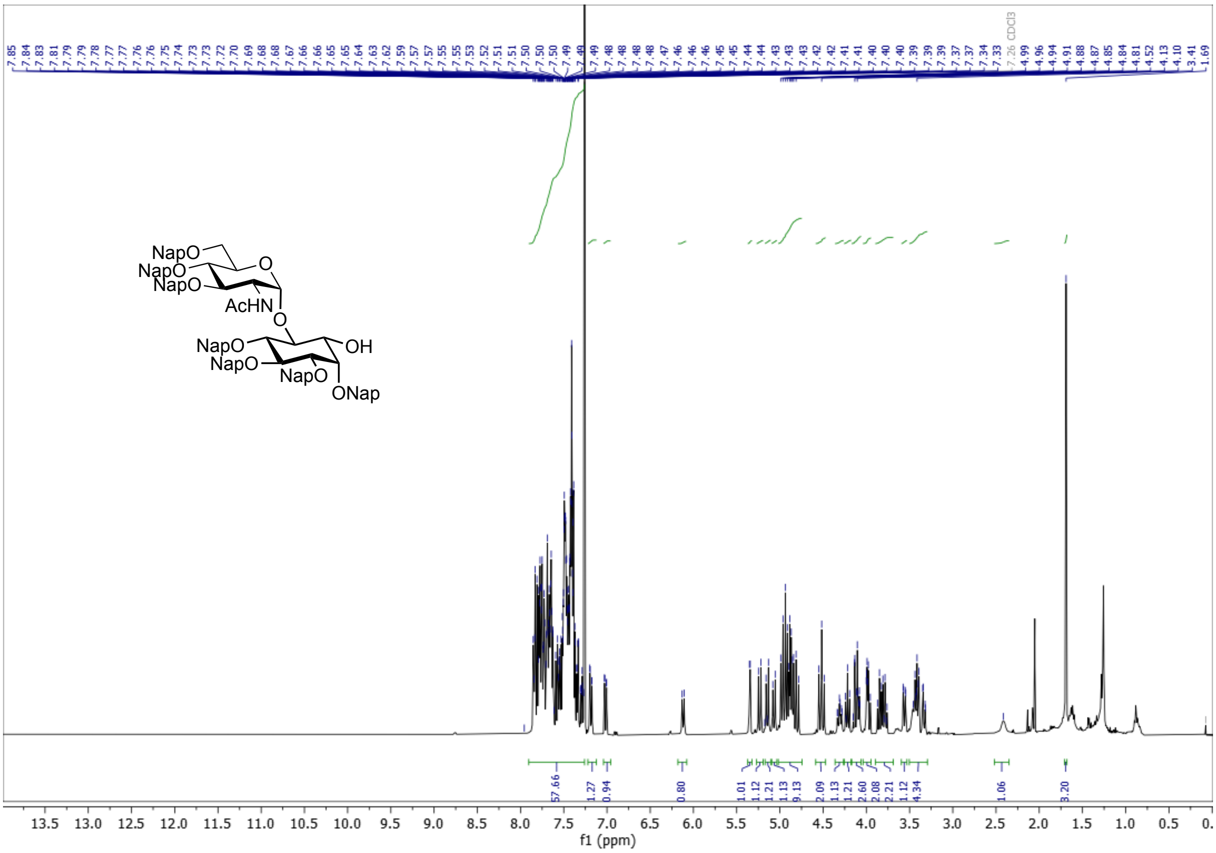

^{13}C-NMR pseudodisaccharide **21a**

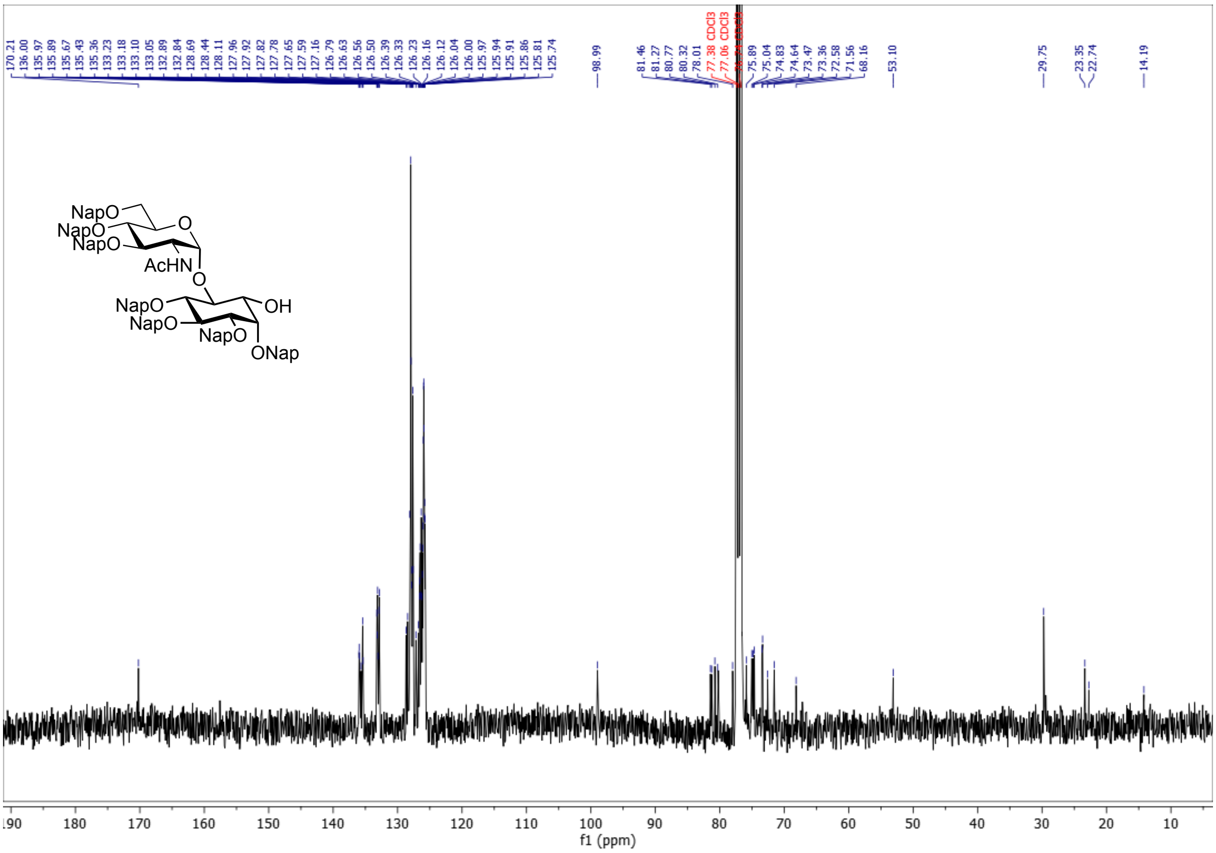

HSQC-NMR spectrum of pseudodisaccharide **21a**

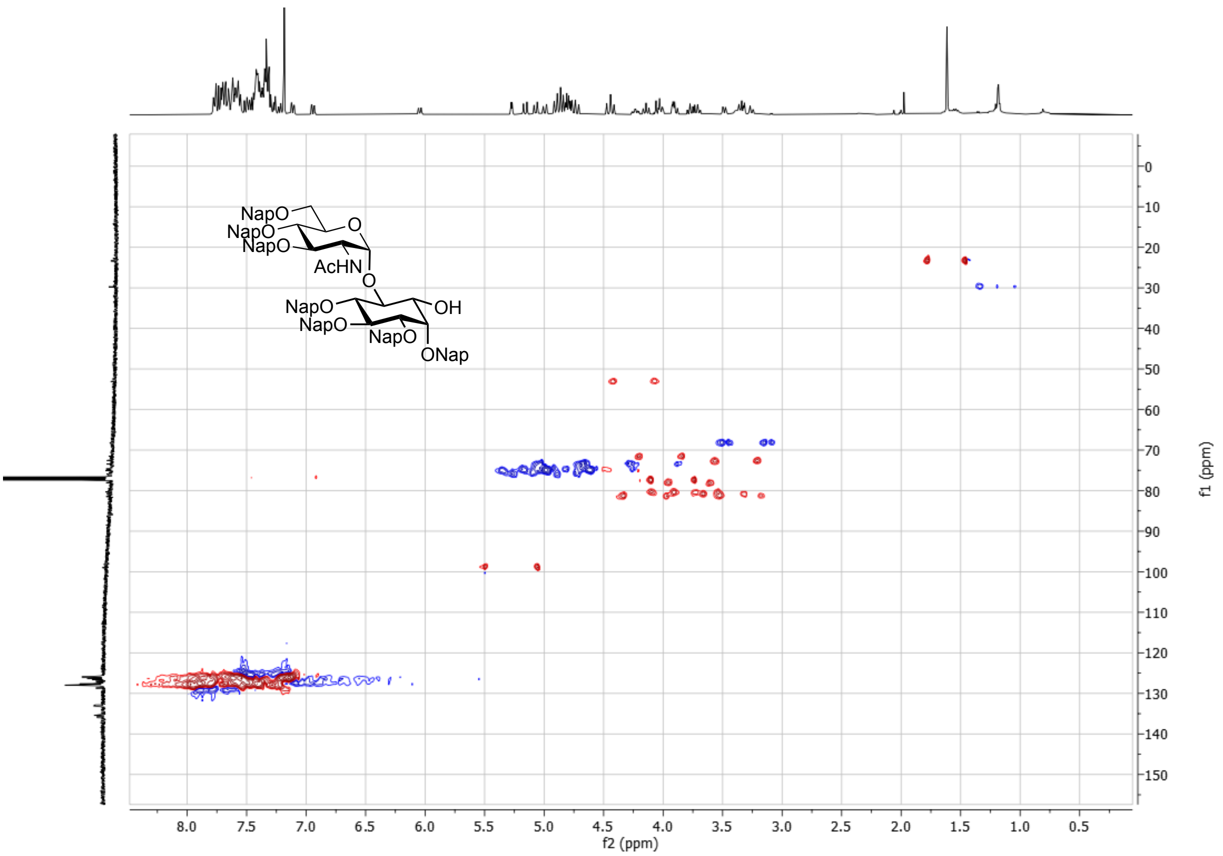

^{1}H-NMR spectrum of protected glycolipid **23**

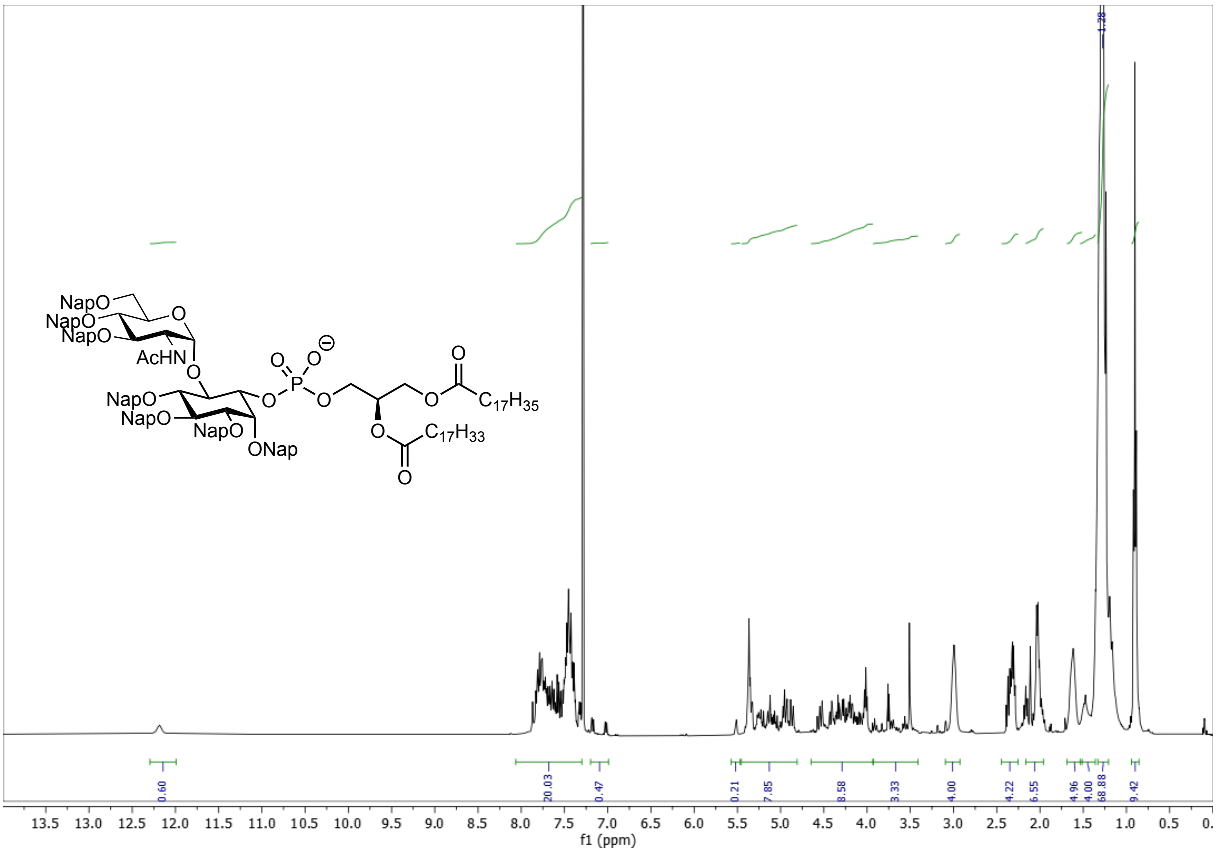

^{13}C-NMR spectrum of protected glycolipid **23**

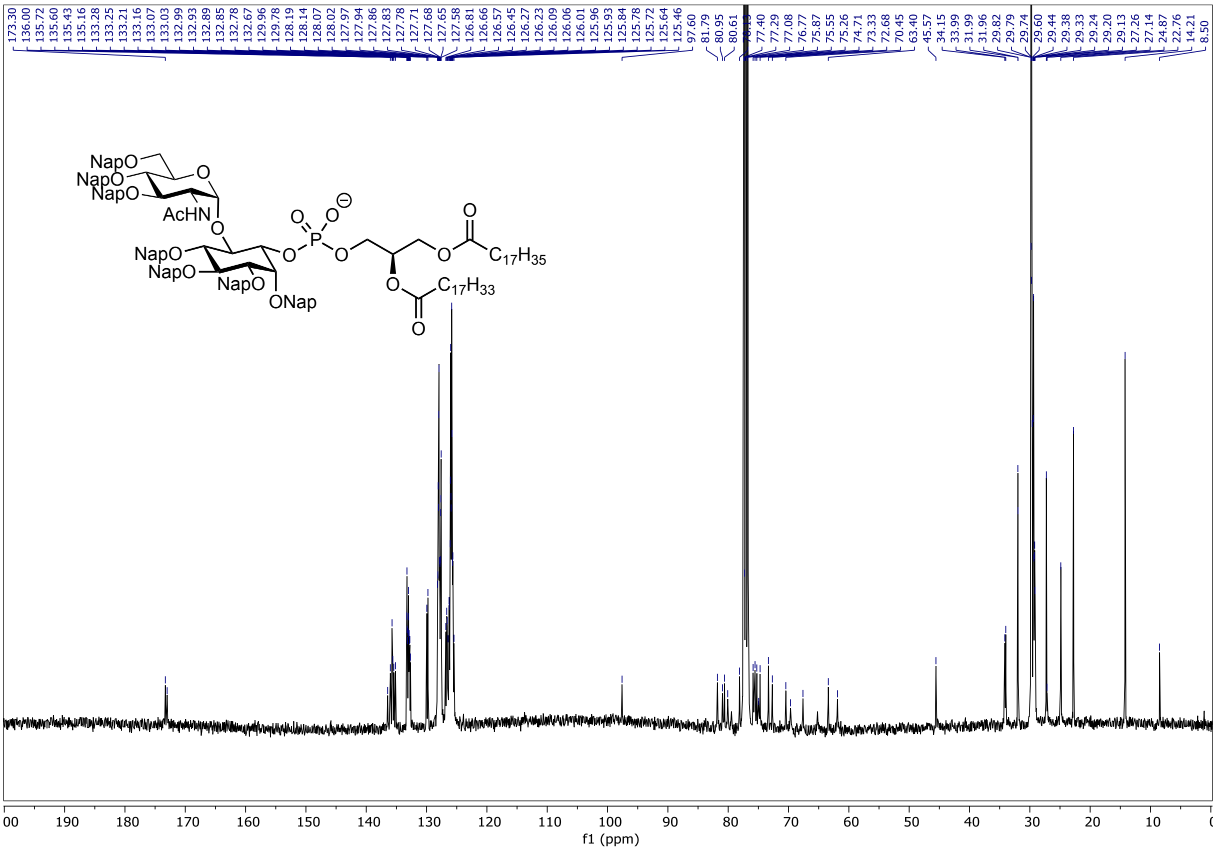

^{31}P-NMR spectrum of protected glycolipid **23**

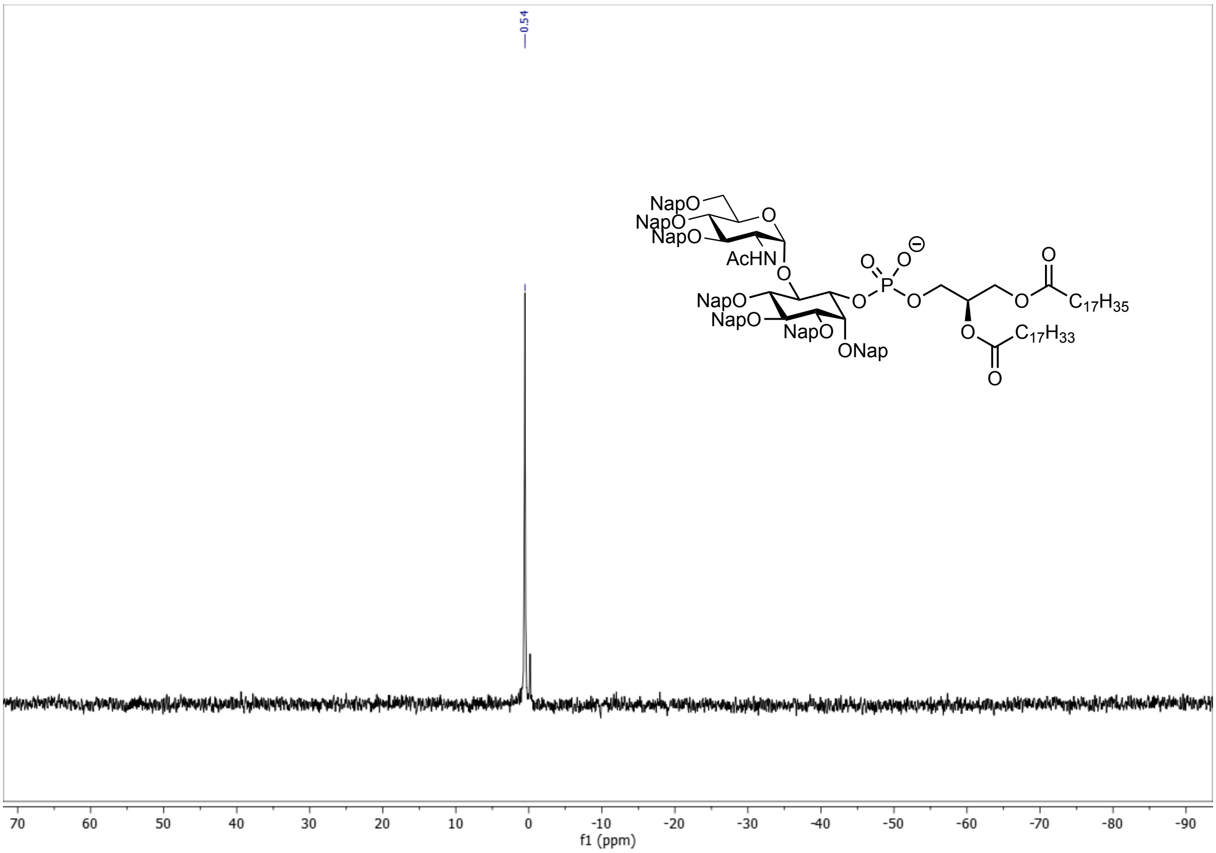

^{1}H-NMR spectrum of glycolipid **3**

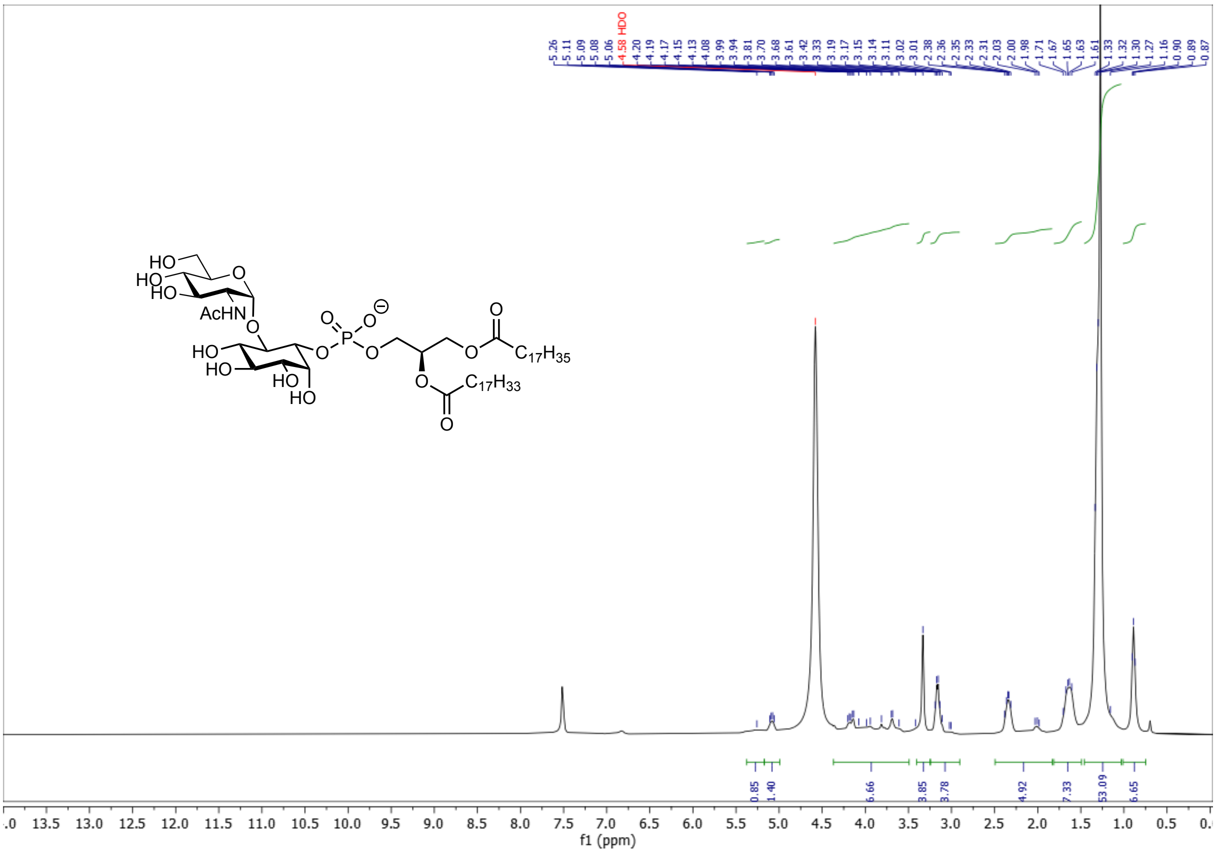

^{31}P-NMR spectrum of glycolipid **3**

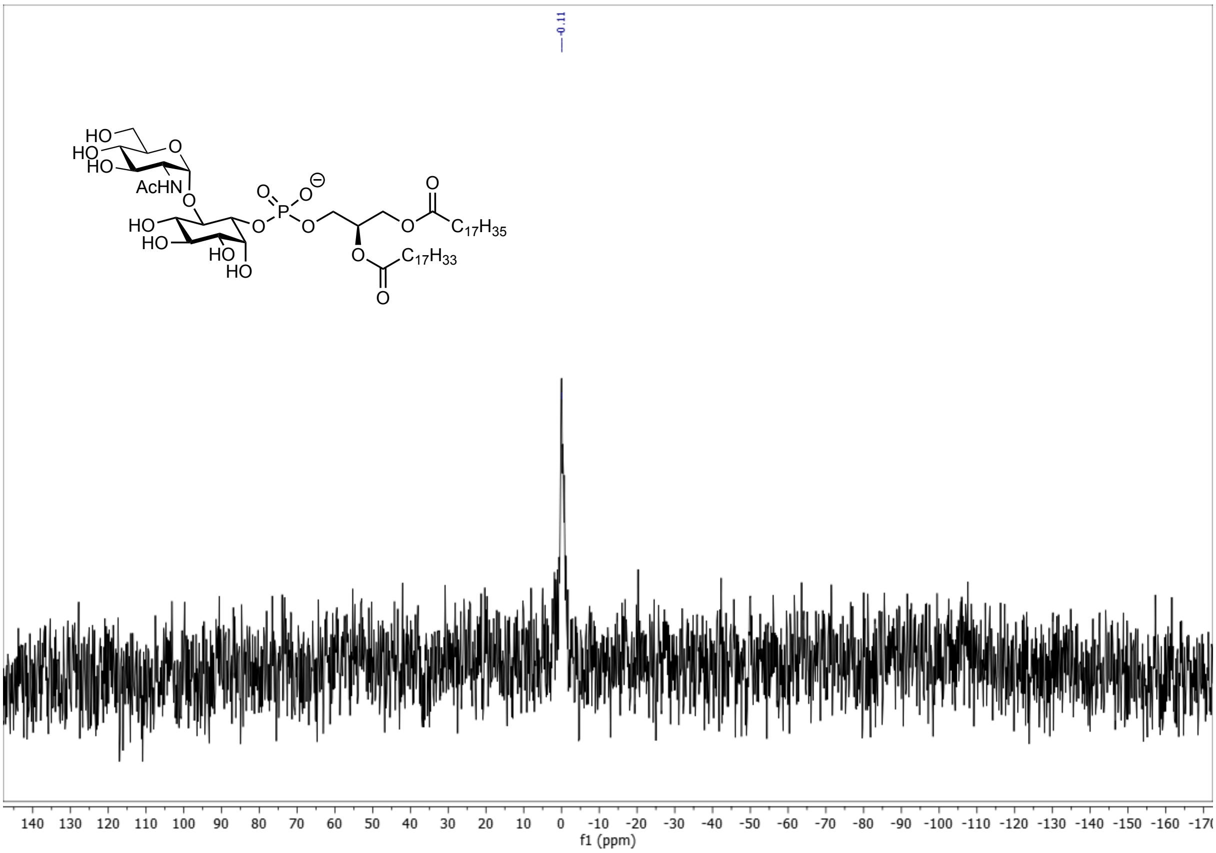

^{1}H-NMR spectrum of protected glycolipid **25**

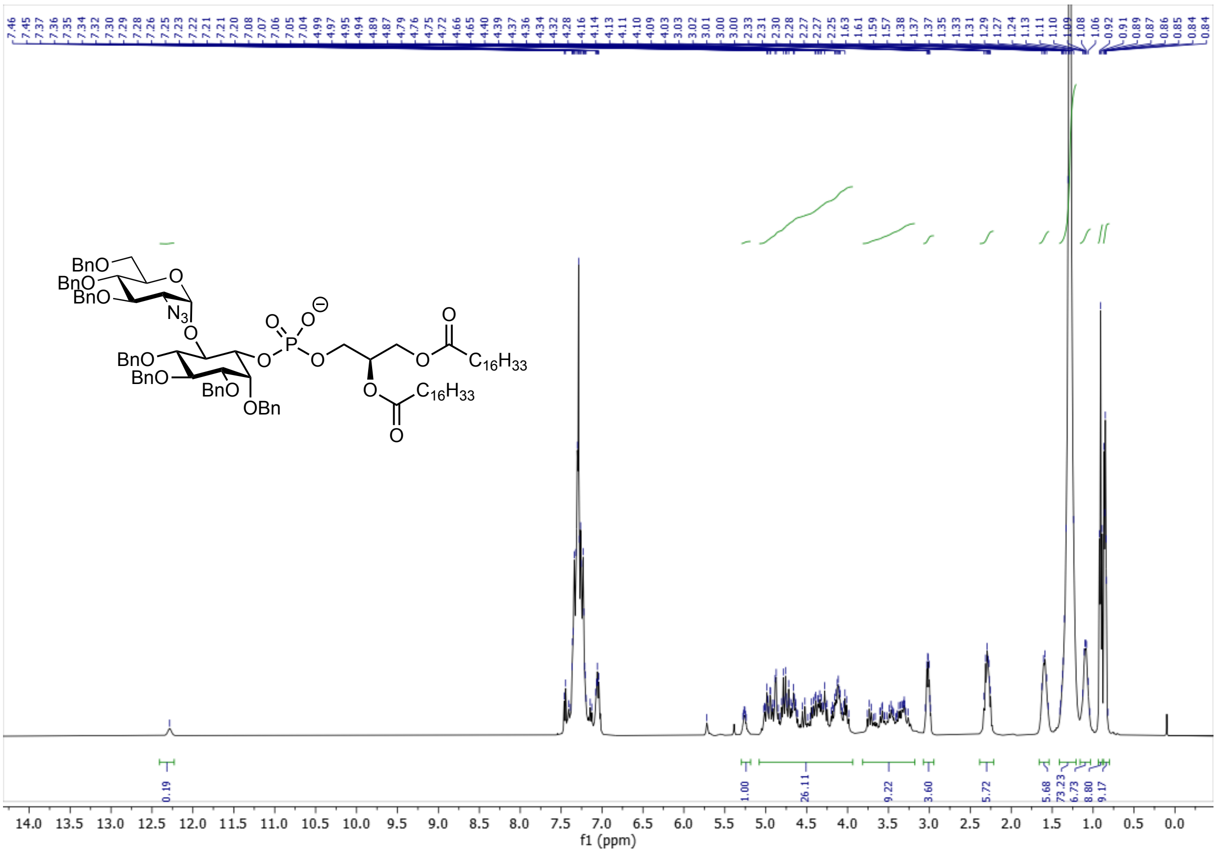

^{31}P-NMR spectrum of protected glycolipid **25**

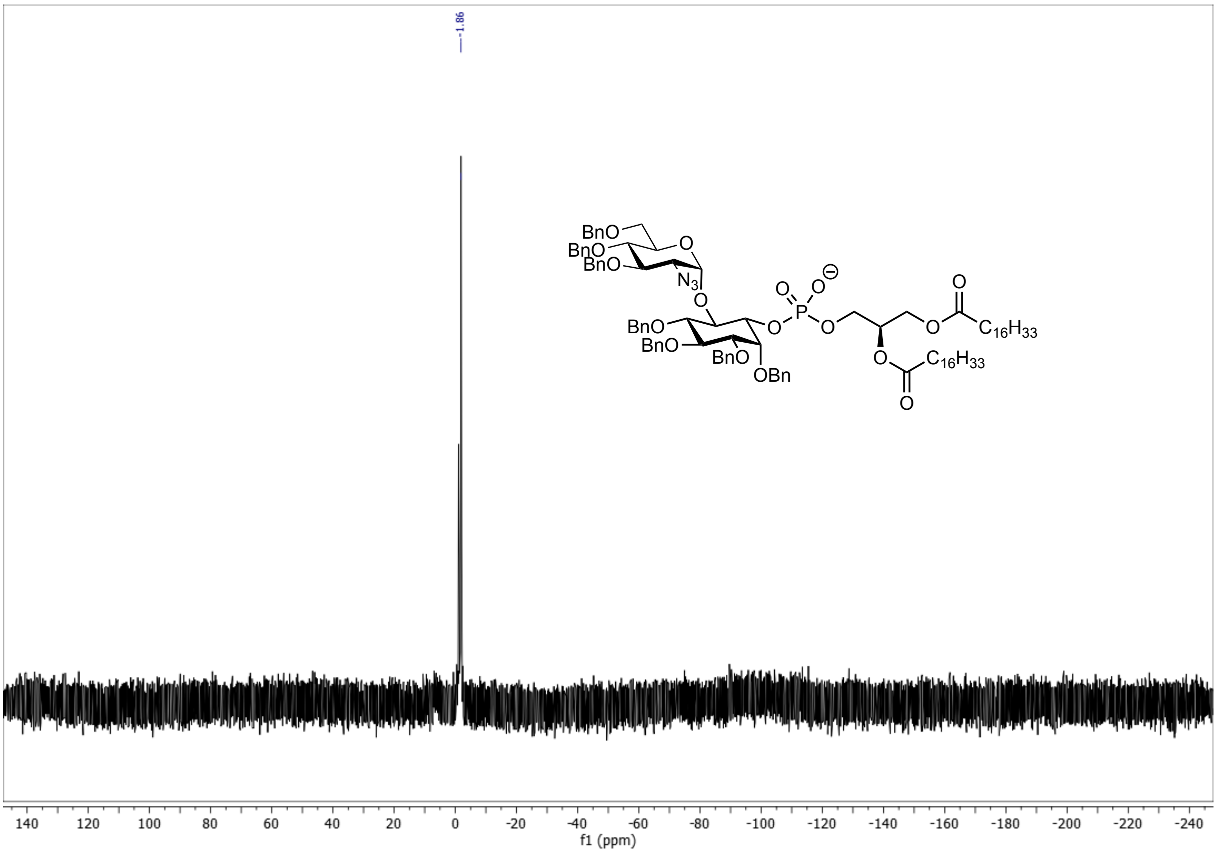

^{1}H-NMR spectrum of glycolipid **4**

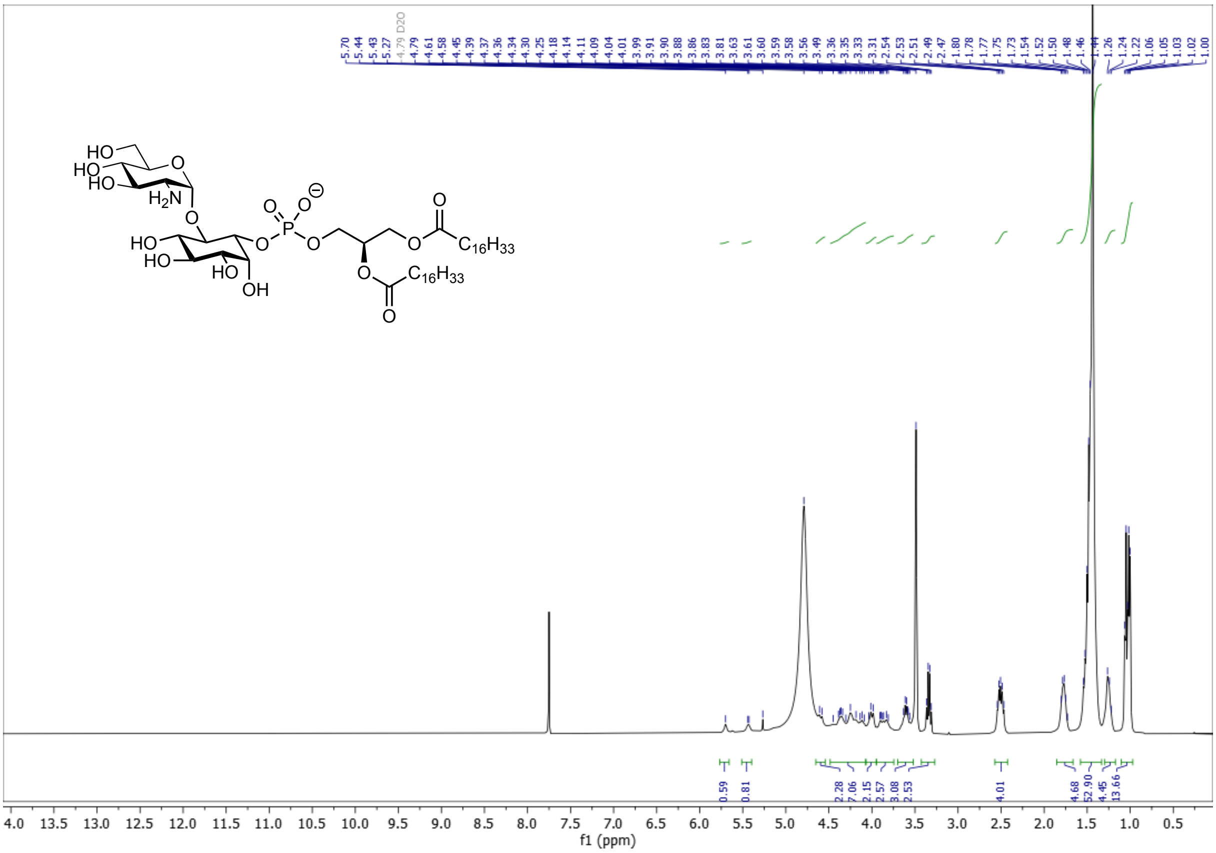

^{13}C-NMR spectrum of glycolipid **4**

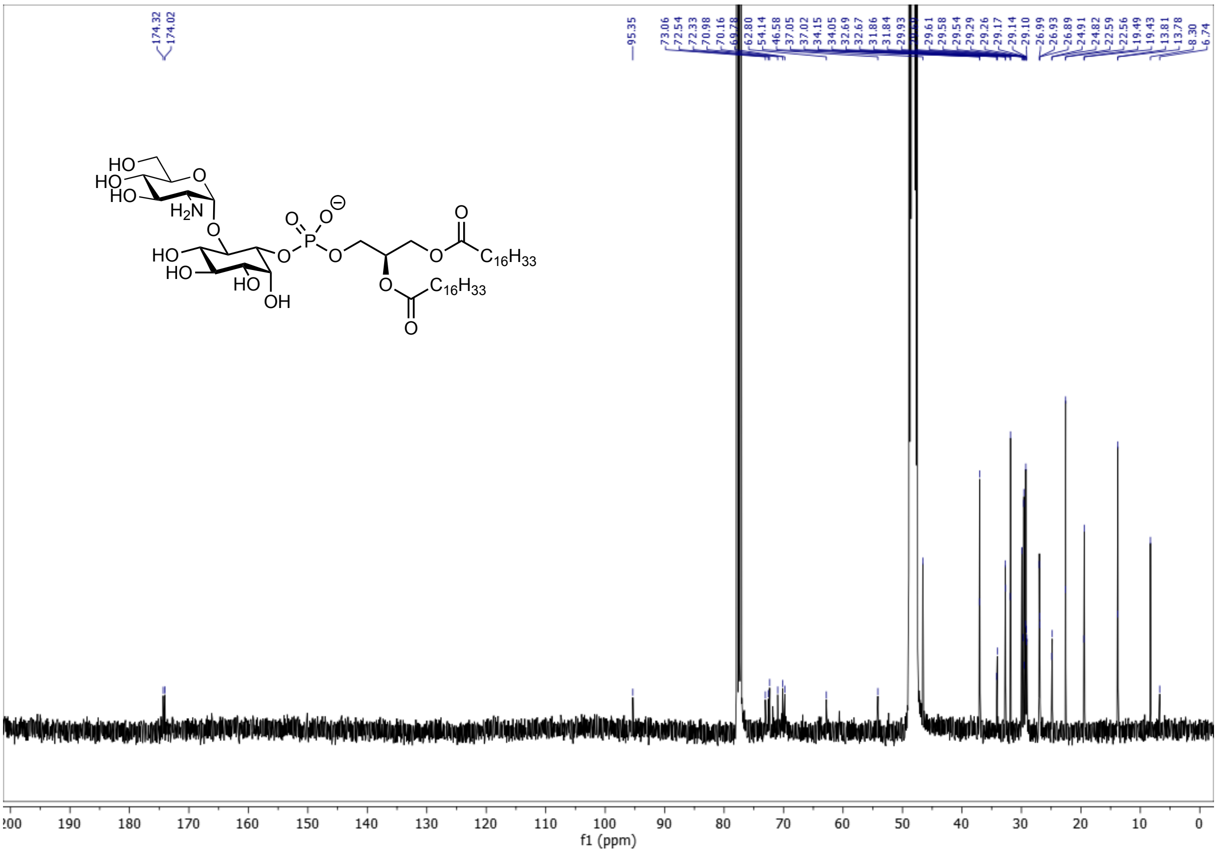

^{31}P-NMR spectrum of glycolipid **4**

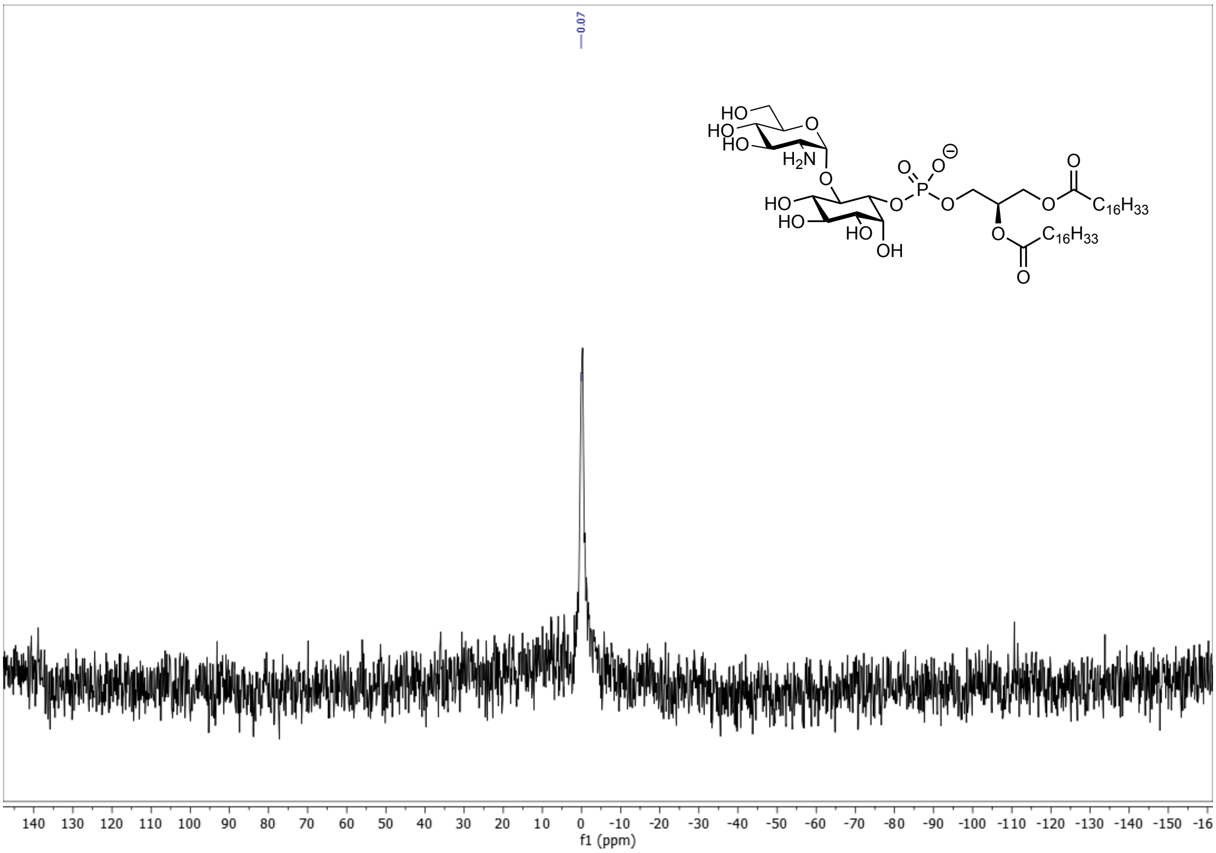

References

[1] Fortunati T., D'Acunto M., Caruso T., Spinella A., *Tetrahedron* **2015**, *71*, 2357-2362.

[2] Stefaniu C., Vilotijevic I., Santer M., Varon Silva D., Brezesinski G., Seeberger P. H., *Angew. Chem. Int. Ed.* **2012**, *51*, 12874-12878.

[3] Jaehne G., Holla W., Krone V., Riedel J., Heuer H., *Vol. DE102010015123A1* (Ed.: D. P.-u. Markenamt), Germany, **2011**, pp. 1-97.

[4] Lee B. Y., Seeberger P. H., Varon Silva D., *Chem. Commun.* **2016**, *52*, 1586-1589.
